# Oral Structural and Functional Impairment in Relation to Depressive Outcomes in Older Adults: A Systematic Review and Meta-Analysis of Observational Studies

**DOI:** 10.3390/diseases14090309

**Published:** 2026-08-26

**Authors:** Lavinia-Alexandra Moroianu, Laurentiu Dragus, Stefan Rosca, Magdalena Rusu Negraia, Valeriu Ardeleanu, Rares Nicolae Vadana, Ionelia State, Georgel Mihu, Madalina Nicoleta Matei, Simona Dana Mitincu Caramfil

**Affiliations:** 1 Faculty of Medicine and Pharmacy, Research Centre in the Medical-Pharmaceutical Field, “Dunărea de Jos” University, 800008 Galați, Romania; lavinia.moroianu@yahoo.com (L.-A.M.); laurentiu.dragus@ugal.ro (L.D.); vadana.rares@yahoo.com (R.N.V.); madalina.matei@ugal.ro (M.N.M.); simona.mitincu@ugal.ro (S.D.M.C.); 2 Faculty of Kinesiotherapy, “Dunărea de Jos” University, 800008 Galați, Romania; valeriu.ardeleanu@gmail.com; 3 Public Health Directorate, 800055 Galați, Romania; ionelia.state@yahoo.com; 4 Cross-Border Faculty, “Dunărea de Jos” University, 800008 Galați, Romania; george.mihu@ugal.ro

**Keywords:** older adults, late-life depression, oral impairment, oral health-related quality of life, gerontopsychiatry, geriatric psychiatry, multidisciplinary assessment, observational studies, systematic review, meta-analysis

## Abstract

Background/Objectives: Oral structural and functional impairment and depressive outcomes frequently coexist in older adults, but the strength and interpretation of their association remain uncertain. This systematic review and meta-analysis evaluated whether poorer oral status or function is associated with depressive outcomes in older populations. Methods: Six databases were searched for observational studies published from 1 January 2016 to 5 January 2026, with an update on 19 April 2026. Eligible studies examined oral impairment as the exposure and depressive outcomes as the endpoint in older adults. Risk of bias was assessed using Newcastle–Ottawa criteria and certainty using GRADE. Comparable adjusted odds ratios (ORs) were pooled using random-effects meta-analysis with restricted maximum likelihood and Hartung–Knapp adjustment; non-comparable metrics were synthesized separately. Results: Thirty studies were included; fifteen contributed to the primary OR meta-analysis. In the primary model, poorer oral status or function was associated with higher odds of adverse depressive outcomes (OR = 1.61, 95% CI: 1.31–1.99), although between-study heterogeneity was substantial (I^2^ = 88.1%). In the trim-and-fill sensitivity analysis, the pooled estimate decreased to OR = 1.29 (95% CI: 0.99–1.69; *p* = 0.0584) and no longer reached conventional statistical significance. The certainty of the primary evidence was very low. Conclusions: Poorer oral structural or functional status is associated with worse depressive outcomes in older adults, but the magnitude is heterogeneous and sensitive to small-study-effect assumptions. The evidence remains observational and of very low certainty and should be interpreted as hypothesis-generating rather than causal or treatment evidence.

## 1. Introduction

### 1.1. Clinical and Public Health Relevance of Late-Life Depression and Oral Impairment

Late-life depression (LLD) is a major public health challenge in aging societies because it is common, frequently under-recognized, and strongly associated with disability, impaired chronic disease management, and increased mortality. Recent evidence shows that depressive syndromes and depressive symptoms remain highly prevalent among older adults worldwide, although reported rates vary across settings, case definitions, and healthcare systems. Burden estimates also indicate that depression in later life continues to contribute substantially to disability-adjusted life years and to rising service demands as populations age. Its clinical management is further complicated by multimorbidity, frailty, cognitive vulnerability, somatic symptom overlap, and treatment resistance, all of which may hinder timely recognition and appropriate care in older populations [1,2,3,4].

Oral impairment in later life is similarly important from both clinical and population-health perspectives. Older adults commonly experience tooth loss, impaired mastication, xerostomia, periodontal disease, dental caries, prosthetic problems, and reduced oral health-related quality of life (OHRQoL). These conditions may compromise nutrition, speech, comfort, autonomy, and social participation. Recent epidemiological and policy-oriented work has increasingly positioned oral health within healthy-aging research and public health planning, emphasizing that oral conditions in older adults should be viewed as functionally important, systemically relevant, and potentially modifiable determinants of well-being rather than as unavoidable consequences of aging [5,6,7,8].

The intersection between LLD and oral impairment is therefore of direct relevance to geriatric medicine and geriatric psychiatry. Oral impairment may reduce food choice, self-confidence, communication, and social engagement, whereas depression may worsen oral self-care, decrease help-seeking, increase symptom burden, and accelerate functional decline. This bidirectional clinical context highlights the need for integrated assessment in older adults. At the population level, it also reveals a persistent service gap: although healthy aging increasingly promotes patient-centered and function-oriented care, oral health is still often separated from mental health pathways, and depression remains insufficiently integrated into geriatric oral-health frameworks. Together, these gaps strengthen the rationale for a focused synthesis of observational evidence examining whether structural and functional oral impairment is consistently associated with depressive outcomes in older adults [9,10,11,12].

### 1.2. Biological, Functional, and Psychosocial Pathways Linking Oral Impairment to Depressive Outcomes

Several biologically plausible pathways may help explain the observed association between oral structural and functional impairment and depressive outcomes in later life. One major pathway involves chronic oral inflammation and dysbiosis, which may amplify systemic inflammatory signaling, endothelial dysfunction, and neuroimmune activation, all of which have been implicated in late-life depression. Recent work on the oral–brain axis has proposed potential links between alterations in oral microbial diversity and composition and depressive states involving inflammatory mediators, microbial metabolites, and disruptions in tryptophan-related pathways relevant to serotonergic regulation. In older adults, who commonly experience low-grade chronic inflammation and multimorbidity, oral inflammatory burden may therefore act not only as a local condition but also as an additional biological stressor interacting with established pathways of affective vulnerability [13,14,15,16,17].

A second pathway is functional and nutritional. Tooth loss, reduced occlusal support, oral frailty, xerostomia, and impaired mastication may restrict food choice, reduce dietary diversity, and increase the risk of malnutrition, sarcopenia, and physical frailty. Contemporary evidence indicates that masticatory dysfunction in older adults is associated with poorer nutritional status and may also affect cerebral activation, cognitive performance, and broader functional reserve. These mechanisms are particularly relevant in geriatric populations because oral impairment may simultaneously hinder eating, reduce protein and micronutrient intake, and worsen fatigue and physical capability, all of which are themselves linked to depressive symptoms and reduced resilience in older age [18,19,20,21].

A third pathway is psychosocial and may be especially important in community-dwelling older adults. Oral impairment can affect appearance, speech, confidence in social interaction, willingness to smile, and comfort when eating in public, thereby diminishing oral health-related quality of life and increasing the likelihood of embarrassment, withdrawal, loneliness, and social isolation. Recent evidence suggests that oral health, mental health, social participation, and socioeconomic position are closely interconnected, while meta-analytic data in older populations indicate that social isolation is itself associated with a higher risk of depression. Accordingly, oral impairment may be associated with depressive outcomes not only through biological stress or physical dysfunction, but also by undermining self-image, daily functioning, and social connectedness in later life [22,23,24].

These pathways are likely to overlap rather than operate independently in later life. For example, impaired mastication may restrict dietary intake while simultaneously reducing enjoyment of meals and participation in social eating; oral pain, xerostomia, tooth loss, or prosthetic difficulties may adversely affect comfort, appearance, communication, self-esteem, and perceived quality of life; and the resulting nutritional, functional, and psychosocial burden may coexist with inflammatory and frailty-related vulnerability. Conversely, depressive symptoms may themselves be associated with poorer oral self-care, reduced motivation, altered eating patterns, lower healthcare utilization, and social withdrawal, potentially reinforcing oral difficulties. The oral–depression relationship should therefore be understood as potentially multidirectional and embedded within shared geriatric vulnerabilities rather than as a single causal pathway from oral impairment to depression.

### 1.3. Why a New Review Was Needed: Evidence Gaps, Redundancy Risks, and the Rationale for a Focused Observational Review

Recent reviews have suggested that oral problems and depressive outcomes are linked in later life, but most have combined broad oral-health constructs, mixed age groups, mixed analytic directions, or multiple neuropsychological outcomes within the same evidence framework [25,26,27,28]. As a result, they are informative at a general level but less suitable for gerontopsychiatric interpretation of oral structural and functional impairment as an exposure domain in older adults.

The present review was designed to address that gap by restricting eligibility to observational studies in older adults, enforcing a fixed oral-exposure-to-depression-outcome direction, separating major oral-exposure families, and avoiding pooled synthesis across non-equivalent effect metrics [29,30,31,32,33,34,35,36]. The observational restriction was chosen because the review question concerned associations between naturally occurring oral structural or functional impairment and depressive outcomes in older populations, rather than the efficacy of a specific dental, nutritional, psychiatric, or other intervention. Including intervention studies would have introduced fundamentally different exposure definitions, comparators, treatment effects, and inferential targets, thereby reducing the interpretability of a synthesis intended to characterize epidemiological associations. Restricting the review to observational designs also allowed cross-sectional and longitudinal evidence to be examined within a common exposure–outcome framework, while preserving the distinction between contemporaneous and temporally ordered associations. The contribution of this review therefore lies not only in topic relevance, but in providing a more clinically interpretable synthesis for geriatric psychiatry that preserves exposure specificity, temporal meaning where possible, and effect–metric integrity.

More specifically, the present meta-analysis provides a dedicated OR-only quantitative synthesis of oral-impairment-to-depression associations in older adults, while retaining RR/PR estimates and coefficient-based findings in separate secondary syntheses rather than combining statistically non-equivalent measures. It further complements the pooled estimate with heterogeneity, prediction-interval, subgroup, meta-regression, influence, leave-one-out, funnel-asymmetry, and trim-and-fill analyses, thereby providing information on the magnitude, robustness, and between-study variability of the association that is not captured by a broad narrative summary alone.

### 1.4. Study Objective and Review Question

Against this background, the present review addressed a focused question of direct relevance to geriatric psychiatry and healthy-aging research: in older adults, are indicators of oral structural and/or functional impairment associated with depressive outcomes, including depression diagnosis, depressive symptoms, incident depression, and depression severity? The aim was to identify, critically appraise, and synthesize observational evidence in which oral impairment was explicitly treated as the exposure and depression-related outcomes were explicitly treated as the dependent endpoints. This framing was chosen because recent research on oral frailty, oral function, and aging increasingly supports the view that oral exposures are multidimensional and clinically meaningful, yet they remain insufficiently integrated into geriatric mental-health inference unless they are examined with clear exposure definitions, age framing, and analytic directionality [37,38,39].

The primary objective was to determine whether oral structural and/or functional impairment in older adults is associated with adverse depression-related outcomes. Secondary objectives were to examine whether the observed association differed descriptively across major oral-exposure families, whether it remained directionally consistent in adjusted observational estimates, and whether longitudinal evidence supported the same adverse pattern seen in cross-sectional analyses. These objectives were aligned with recent literature indicating that oral frailty, oral function, oral health-related quality of life (OHRQoL), and related geriatric oral indicators are linked to adverse aging trajectories, psychosocial restriction, and depression-relevant health transitions, while the magnitude, comparability, and temporal interpretation of these associations still require more rigorous review design and more disciplined synthesis [40,41].

From a gerontopsychiatric perspective, oral structural and functional impairment should not be viewed solely as a somatic condition, but as a multidimensional factor interacting with affective disorders, cognitive decline, and psychosocial vulnerability in later life. Accordingly, examining oral impairment within a geriatric psychiatry framework may provide clinically relevant information for multidisciplinary assessment, prevention-oriented care, and patient-centered management in aging populations.

## 2. Materials and Methods

### 2.1. Reporting Framework, Review Design, and Protocol Status

This study was conducted as a systematic review and meta-analysis of observational studies evaluating whether oral structural and functional impairment is associated with depressive outcomes in older adults. The review used a prespecified exposure–outcome direction, with oral impairment consistently treated as the exposure and depression-related measures as the outcomes. Reporting followed the Preferred Reporting Items for Systematic Reviews and Meta-Analysis (PRISMA) 2020 statement. A completed PRISMA 2020 checklist and PRISMA 2020 flow diagram are provided with the main manuscript and Supplementary Materials. The review was not prospectively registered. Specifically, no prospective registration was obtained in PROSPERO, the Open Science Framework (OSF) Registries, or another public registry. Because the review was not prospectively registered, the eligibility criteria, exposure families, effect–selection rules, and quantitative–synthesis hierarchy were locked before completion of the quantitative workflow and were applied without post hoc modification. The frozen effect–selection dataset and the reconciliation of downstream analytic streams are provided in the Supplementary Materials to maximize transparency and auditability [42,43,44,45,46,47].

No retrospective registration was undertaken because the review and quantitative synthesis had already progressed beyond the stage at which registration could serve its intended prospective function. We therefore report the absence of prospective registration transparently and provide the frozen effect-selection dataset and analytic-stream reconciliation as auditability safeguards; these measures should not be interpreted as substitutes for prospective public preregistration. Because these safeguards were generated within the review workflow and were not prospectively time–stamped in a public registry, they cannot provide independent verification that the eligibility criteria, exposure definitions, effect–selection rules, and analytic plans were fixed before the review results were known.

### 2.2. Eligibility Criteria

Eligibility criteria were defined a priori and applied consistently during title/abstract screening and full-text review. Studies were eligible if they used an observational design and examined older adults or a separable older-adult subgroup within a broader-age population. For review purposes, a study met the older-adult population criterion when the analytic sample was explicitly geriatric (typically ≥60 or ≥65 years) or when an older-adult subgroup estimate was reported separately and could be extracted without ambiguity. To reduce construct drift, retained oral exposures were later classified into prespecified review-level families distinguishing structural impairment, functional impairment, symptom-based impairment, and broader proxy indicators. This operationalization was consistent with contemporary geriatric oral-health frameworks that distinguish oral frailty and oral hypofunction as clinically meaningful but non-identical multidimensional constructs [48,49,50,51].

Eligible outcomes were restricted to depression-related endpoints, including diagnosed depression, depressive symptoms, incident depression, and depression severity, whether operationalized as binary or continuous variables. Outcome ascertainment could be based on diagnosis or on validated instruments commonly used in older populations, including the Geriatric Depression Scale (GDS), Patient Health Questionnaire (PHQ), Center for Epidemiologic Studies Depression Scale (CES–D), Self-Rating Depression Scale (SDS), Euro–D, and comparable measures with established psychometric support in older adults [52,53,54,55]. Studies were excluded if depression was treated as the predictor and oral status as the outcome, if the endpoint was not specifically depressive in nature, if oral exposure could not be isolated from a broader non-oral construct, or if the report was a review, commentary, protocol, conference abstract without extractable data, case report, animal study, in vitro study, or intervention study outside the observational scope of the review. The final distribution of included studies across the qualitative and quantitative syntheses is summarized in Supplementary Materials Table S2, and detailed study characteristics are presented in Table S4.

### 2.3. Information Sources and Search Strategy

The literature search was conducted in six bibliographic databases: Medical Literature Analysis and Retrieval System Online (MEDLINE)/PubMed, Scopus, Web of Science Core Collection, Embase, American Psychological Association (APA) PsycInfo, and Cumulative Index to Nursing and Allied Health Literature (CINAHL). The initial searches covered publications from 1 January 2016 to 5 January 2026 and were updated on 19 April 2026 using the same search framework and eligibility criteria; newly eligible records identified by the updated search were incorporated into the final review corpus. Search strategies were developed for each database using platform-appropriate controlled vocabulary, free-text terms, Boolean operators, and field tags, with syntax adapted to the indexing and search structure of the respective database [56,57,58,59,60].

Across databases, the search syntax combined three concept blocks: older populations, oral structural or functional impairment, and depressive outcomes. Population terms included aged, elderly, older adult(s), older people, senior(s), and geriatric terms. The oral-exposure block included terms relating to tooth loss, missing or remaining teeth, dentition, edentulism, mastication or chewing difficulty, oral function, oral hypofunction, oral frailty, xerostomia or dry mouth, oral pain, dentures/prostheses, oral health, and oral health-related quality of life. The depressive-outcome block included depression, depressive disorder, depressive symptoms, late-life depression, and incident depression. No language restrictions were applied. Restrictive observational study-design filters were not used at the database-search stage because they could reduce retrieval sensitivity; observational design eligibility was instead determined during title/abstract screening and full–text assessment. Retrieved records were combined in a common reference–management workflow and deduplicated before screening using a structured approach [61]. The complete database-specific search strategies—including exact search strings, controlled–vocabulary and free-text terms, Boolean structure, field tags, search dates, resource types searched, and applicable filters or restrictions—are provided in Supplementary Materials Table S1.

The 2016 cutoff was defined a priori as a scope restriction rather than as a biological or methodological threshold, and it should not be interpreted as implying that studies published before this date are necessarily less informative. Its purpose was to delimit a clinically contemporary evidence window during a period of increasing attention to multidimensional oral functional impairment and oral frailty in older populations, while maintaining a focused review scope.

### 2.4. Study Selection and Eligibility Adjudication

Study selection comprised title/abstract screening followed by full-text eligibility assessment. After a calibration exercise, two reviewers independently screened records against the prespecified eligibility criteria, with potentially eligible records proceeding to full-text review. Full texts were independently assessed by the same reviewers for population eligibility, oral exposure, depressive outcome, analytic direction, study design, and the availability of an extractable effect estimate. Disagreements were resolved by consensus or, when necessary, by a third reviewer. Reasons for full-text exclusion are reported in Supplementary Materials Table S3 [62,63,64,65,66,67].

### 2.5. Data Extraction and Data Management

Data were extracted using a structured, piloted form capturing study characteristics, population framing, oral exposure and depressive outcome definitions, adjustment status, effect metric and estimate, confidence interval, and analytic-stream assignment. One reviewer performed primary extraction and a second independently verified all fields against the full text; unresolved discrepancies were adjudicated by a third reviewer. When multiple eligible estimates were available, adjusted estimates best matching the prespecified exposure contrast, outcome definition, older-adult population criterion, and analytic stream were prioritized before dataset freezing.

Extracted data were organized in a master dataset and assigned to prespecified analytic streams according to metric comparability and inferential role. The frozen effect-selection dataset and stream assignments are provided in Supplementary Table S6, with reconciliation of the derived analytic streams in Table S7. Analyses were performed in Python (version 3.12.13) using reproducible scripted workflows and independently cross-checked [43,44,66,67].

### 2.6. Exposure Definitions, Outcome Definitions, and Directionality Rules

Eligible oral exposures were retained as defined in the primary studies and then assigned to prespecified review-level families to preserve construct fidelity while enabling structured synthesis. For the purposes of this review, oral structural impairment referred to deficits in the anatomical or dentition-related substrate of the oral system, including tooth loss, edentulism, reduced number of remaining teeth, severe tooth loss, loss of functional dentition, and reduced functional tooth units. Oral functional impairment referred to reduced capacity, performance, or symptom–related integrity of oral functions, including chewing difficulty or disability, oral hypofunction, impaired oral physical function, xerostomia, and salivary dysfunction. Oral frailty was treated as a multidimensional functional construct because contemporary definitions incorporate several manifestations of declining oral function and may also include a structural component such as reduced tooth number [49,50,51,68].

Accordingly, the structural and functional categories used in this review should be understood as analytic groupings rather than as mutually exclusive clinical diagnoses. Broader proxy indicators, including self-rated oral health, denture/prosthetic status, and oral health-related quality of life (OHRQoL), were retained only when they were explicitly analyzed as oral-status indicators relevant to the review question and were interpreted cautiously because they may integrate structural, functional, symptomatic, and psychosocial dimensions rather than represent a single impairment domain [69,70,71]. This family-based classification was used for evidence mapping, subgrouping, and analytic-stream assignment and remained fully traceable to the frozen master dataset and its derived quantitative datasets in Supplementary Materials, particularly Tables S6 and S8–S10. For review-level subgrouping and meta-regression, ascertainment type was classified as: (i) likely clinical/objective or structured, when the retained estimate relied primarily on clinical examination, registry diagnosis, interviewer-administered standardized instruments, or clearly structured survey procedures; (ii) likely self-report or mixed, when the retained estimate relied predominantly on self-reported oral status/function or on mixed ascertainment; and (iii) unclear, when the report did not permit confident classification.

Depressive outcomes were eligible when the primary study operationalized depression as a diagnosis or newly diagnosed depression, a binary depressive-symptom endpoint derived from a recognized assessment instrument, incident depressive symptoms during follow-up, or a continuous measure of depressive symptom burden or severity. No single review-level diagnostic threshold was imposed because the included studies used different validated instruments and study-specific operational definitions; instead, the original outcome definition and cutoff were retained for each study. The assessment instruments represented in the included evidence included the Geriatric Depression Scale (GDS; 5– and 15–item versions), Center for Epidemiologic Studies Depression Scale (CES–D; full and 10–item versions), Patient Health Questionnaire (PHQ–8 and PHQ–9), Euro-D, Self-Rating Depression Scale (SDS), and 30-item General Health Questionnaire (GHQ–30), together with study–specific depressive symptom measures. Among studies using dichotomized scale–based outcomes, reported thresholds included GDS–15 ≥ 5, GDS–15 > 5 or >4, CES–D ≥ 16, CES–D–10 ≥ 4 or ≥10, PHQ–9 ≥ 10, SDS ≥ 40, and GHQ–30 ≥ 7. Continuous scale scores were retained as continuous outcomes and were not dichotomized post hoc. Diagnosis-based and other binary depressive endpoints for which the primary study did not define the outcome through one of these numerical scale thresholds were retained according to the original study definition. Study-specific depressive-outcome definitions, instruments, thresholds, and selected estimates are reported in Supplementary Materials Tables S4 and S6.

For review-level evidence mapping, these original definitions were grouped into four endpoint classes: diagnosis-based depression, binary depressive symptoms, incident depressive outcomes, and continuous depressive symptom or severity measures. Only one primary estimate per study per analytic stream was carried forward. When multiple eligible estimates were reported, preference was given to the estimate that best matched the prespecified oral-exposure contrast, older-adult analytic frame, depressive-outcome definition, highest relevant level of covariate adjustment, and intended quantitative or structured narrative stream. A strict directionality rule was applied throughout: retained analyses had to evaluate oral structural or functional impairment as the exposure and depressive outcomes as the dependent endpoints. Studies modeling depression as the predictor of oral status were excluded irrespective of statistical significance. When necessary, contrasts were directionally harmonized at the interpretive level so that all retained estimates mapped to the same substantive meaning, namely greater oral impairment in relation to greater depressive burden; any metric-level transformation required for synthesis was handled separately in the effect-size harmonization procedures described in Section 2.8. This explicit exposure-outcome specification was adopted to reduce reverse-causation ambiguity, improve comparability across heterogeneous observational designs, and preserve transparent linkage between study-level operational definitions and the final evidence streams [72,73].

### 2.7. Risk-of-Bias Assessment and Certainty of Evidence

Risk of bias was assessed independently by two reviewers, with disagreements resolved through discussion and, when necessary, adjudication by a third reviewer. Because all included studies were observational, study-level methodological quality was evaluated using the Newcastle–Ottawa Scale (NOS) with design-appropriate operationalization [74]. Cohort studies were assessed using the standard NOS framework, whereas analytical cross-sectional studies were evaluated using a prespecified adapted version that preserved the three NOS domains and the conventional 0–9 star scale. The Selection domain contributed up to 4 stars and considered the representativeness and definition of the study population, sampling or recruitment procedures, ascertainment of the oral exposure, and relevant response or completeness characteristics. The Comparability domain contributed up to 2 stars and reflected the extent to which analyses controlled for major demographic confounders and additional relevant socioeconomic, behavioral, or health-related covariates. The Outcome/Exposure domain contributed up to 3 stars and considered the validity and consistency of depressive-outcome and oral-exposure ascertainment and, for longitudinal studies, the adequacy of follow-up and completeness of outcome assessment. Total NOS scores ranged from 0 to 9 stars and were interpreted descriptively as high methodological quality (7–9 stars), moderate methodological quality (5–6 stars), or low methodological quality (≤4 stars). Each domain judgment and its study–specific rationale were recorded before the evidence base was frozen. Total NOS scores were used descriptively and as study-level moderators in exploratory analyses, including both categorical quality grouping and the continuous NOS total score. The complete study-level domain scores, total scores, quality categories, and supporting rationales are reported in Supplementary Materials Table S5, Panel A.

For analytical cross-sectional studies, the adapted scoring was operationalized explicitly as follows. In the Selection domain (0–4 stars), one point was assigned for each adequately satisfied component concerning: (i) clear definition and reasonable representativeness of the target population and analytic sample; (ii) transparent and appropriate sampling or recruitment procedures; (iii) clear definition and structured ascertainment of the oral exposure; and (iv) adequate reporting of participation, response, or analytic–sample completeness. In the Comparability domain (0–2 stars), two stars indicated adjustment for core demographic confounders together with additional relevant socioeconomic, behavioral, functional, or clinical covariates; one star indicated partial confounder control or incompletely described multivariable adjustment; and zero stars indicated no meaningful adjustment. In the Outcome/Exposure domain (0–3 stars), three stars indicated clearly defined and sufficiently robust exposure and depressive-outcome ascertainment, consistent measurement procedures, and adequate reporting of outcome or analytic completeness; two stars indicated one material limitation or uncertainty in these features; one star indicated multiple important limitations while retaining sufficient information for appraisal; and zero stars indicated inadequate or insufficiently reported ascertainment. Cross-sectional design itself did not trigger an automatic fixed star deduction; lack of temporal separation was recorded as an interpretative limitation and was considered together with the strength of measurement and reporting. When information was insufficient to verify that a criterion was satisfied, the more conservative score was assigned. The complete item–level operational rules are reproduced in Supplementary Table S5, Panel A.

The NOS was selected because it allowed a common study-level framework across the mixed observational designs included in this review; however, its limitations were explicitly recognized. In particular, a summary star score may obscure domain–specific sources of bias, involves implicit weighting across items, and does not provide the same structured causal-domain assessment of confounding, selection, measurement, and missing data as more recent risk-of-bias frameworks. We therefore did not interpret NOS totals as direct measures of overall internal validity or use them to determine certainty of evidence. Alternative domain-based tools were considered, but a retrospective reassessment with a different instrument after completion of the prespecified review workflow was not undertaken because it would introduce a new post hoc appraisal framework and would not constitute an independent sensitivity analysis. Instead, the study-level NOS judgments and their domain rationales are reported transparently in Supplementary Table S5, and the relationship between methodological quality and effect magnitude was examined using both categorical and continuous NOS moderators.

Certainty of evidence was assessed separately using the Grading of Recommendations Assessment, Development and Evaluation (GRADE) framework. In accordance with GRADE principles, certainty judgments were made at the level of the relevant synthesized body of evidence rather than at the level of individual studies. Observational evidence bodies were initially considered low certainty and were then evaluated across the standard GRADE domains, including risk of bias, inconsistency, indirectness, imprecision, and publication bias, together with any factors that could justify rating up when appropriate. This distinction between study-level methodological quality and body-level certainty was maintained deliberately because the two constructs are related but not interchangeable. Study-level NOS assessments are reported in Panel A of Supplementary Materials Table S5, whereas body-level GRADE certainty judgments for the synthesized evidence streams are reported in Panel B of the same table [75].

### 2.8. Quantitative-Synthesis Eligibility and Effect-Size Harmonization

Quantitative-synthesis eligibility was determined after extraction and analytic-stream assignment, retaining one principal estimate per study per stream and prioritizing adjusted estimates that best matched the prespecified exposure contrast, depressive outcome, older-adult population criterion, and relevant covariate adjustment. The primary pooled synthesis was restricted to odds ratios (ORs) for binary depressive outcomes; risk ratio (RR) and prevalence ratio (PR) estimates and coefficient-based measures were retained in separate structured narrative streams because of differences in scale and interpretation [76,77]. Accordingly, the pooled OR represents a cross-domain average association across non-identical oral constructs rather than a clinically uniform effect for a single homogeneous exposure. Pooling was retained because the contributing estimates shared the same effect metric (OR), the same prespecified analytic direction (greater oral impairment as the exposure and greater depressive burden as the outcome), and an older-adult population frame, while the random-effects model explicitly allowed the underlying associations to vary across studies. This decision does not imply that tooth loss, oral frailty, chewing dysfunction, xerostomia, oral health-related quality of life, other oral constructs, or the different depression instruments are clinically equivalent; their non-equivalence was addressed through construct-specific subgroup analyses, meta-regression, and prediction-interval estimation.

ORs were transformed to the natural logarithmic scale, with standard errors derived from reported 95% confidence intervals when necessary. Directionality was harmonized so that estimates consistently represented greater oral impairment in relation to greater depressive burden. RR/PR and coefficient estimates were not converted to ORs or pooled across incompatible metrics; their original metric and model interpretation were retained in the corresponding secondary evidence streams [78,79].

### 2.9. Statistical Analysis

All quantitative analyses were performed on the frozen analytic datasets using prespecified effect selections and harmonized directionality. Statistical inference was two-sided, and effect estimates, confidence intervals, and derived summary statistics were reported to three decimal places. Because clinical, methodological, and exposure-related heterogeneity was anticipated across the included observational studies, random-effects methods were preferred over common-effect synthesis. Exploratory subgroup analyses and meta-regression were prespecified as descriptive tools for examining heterogeneity structure and were not interpreted as confirmatory tests of effect modification [80,81,82,83,84,85,86,87,88,89,90,91].

#### 2.9.1. Primary OR-Only Random-Effects Meta-Analysis

The primary meta-analysis combined study-specific log odds ratios using inverse–variance weighting under a random-effects model. Between-study variance was estimated with restricted maximum likelihood (REML), given its favorable operating characteristics relative to several alternative τ^2^ estimators, particularly when heterogeneity is non-negligible and study sizes are uneven. Inference for the pooled effect was based on the Hartung–Knapp–Sidik–Jonkman approach, and pooled estimates were back-transformed to the odds ratio scale for presentation. For each pooled model, we reported the summary odds ratio with its 95% confidence interval (CI), the test statistic for the overall effect, Cochran’s Q, I^2^, τ^2^, τ, Higgins’ H, and the 95% prediction interval. The prediction interval was included to reflect the range of effects plausibly expected in a new comparable study, rather than only the average effect across the studies already synthesized [80,81].

#### 2.9.2. Exploratory Subgroup Analyses

Exploratory subgroup analyses were conducted within the primary OR-only meta-analysis to examine whether pooled estimates varied across prespecified study-level characteristics. Subgrouping variables included design class, exposure family, geographic region, ascertainment type, methodological–quality category, and older-adult population framing. These analyses were interpreted strictly as descriptive explorations of heterogeneity. Particular caution was applied to subgroup levels containing only two or three studies, for which confidence intervals and prediction intervals were considered unstable [82].

#### 2.9.3. Exploratory Univariate Meta-Regression

To further explore residual heterogeneity in the primary OR-only meta-analysis, we fitted univariate mixed-effects meta-regression models with one moderator at a time. These analyses were exploratory rather than confirmatory. Given the modest number of studies in the primary meta-analysis, the models were not intended to identify definitive effect modifiers and were interpreted with particular caution when moderator levels were sparse [83,89].

#### 2.9.4. Leave-One-Out and Influence Diagnostics

The robustness of the primary OR-only pooled estimate was examined using leave-one-out analysis, in which the random-effects model was refitted after excluding each study in turn. For each refitted model, we recorded the pooled odds ratio and heterogeneity statistics in order to identify whether any single study materially altered the magnitude, precision, or heterogeneity structure of the summary association. We additionally calculated study-level influence diagnostics, including leverage (hat values), standardized residuals, and Cook’s distance, together with Baujat-type diagnostics summarizing each study’s contribution to the overall heterogeneity statistic and the absolute shift in the pooled effect after exclusion. These procedures were used to distinguish studies that were simply heterogeneous from those that were genuinely influential on the pooled estimate [84,85].

#### 2.9.5. Small-Study Effects and Trim-and-Fill Sensitivity Analysis

Potential small-study effects in the primary OR-only meta-analysis were assessed by visual inspection of the funnel plot and by a regression-based asymmetry test when the number of pooled studies was considered sufficient for such analysis. Because asymmetry tests are sensitive to the number of included studies and may also be influenced by between-study heterogeneity, any non-zero asymmetry signal was interpreted as indicating possible small-study effects rather than definitive publication bias [87,88]. Trim-and-fill analysis was therefore used only as a sensitivity analysis and not as a corrected causal estimate of the true underlying association [90].

#### 2.9.6. Structured Quantitative Narrative Synthesis of RR/PR Studies

Studies reporting risk ratios (RRs) or prevalence ratios (PRs) were synthesized using a structured quantitative narrative approach rather than formal pooled meta-analysis. Pooling RR/PR estimates with ORs was avoided due to differences in underlying risk interpretation and baseline risk dependency, which could introduce bias in mixed-metric meta-analysis. The synthesis was organized according to metric type, study design, outcome temporality, exposure family, adjustment status, and direction of association. This decision was based on the limited number of non-OR studies and on the interpretive differences between prevalence-based and risk-based relative measures, which would have made a secondary pooled ratio model more vulnerable to indirectness than informative for inference. The narrative framework was therefore applied to preserve comparability of interpretation without overstating statistical precision in a sparse and conceptually heterogeneous body of evidence [86].

#### 2.9.7. Structured Quantitative Narrative Synthesis of Beta/Coefficient Studies

Studies reporting continuous-effect estimates, including adjusted beta coefficients, standardized beta coefficients, total effect coefficients, and two-stage least squares (2SLS) coefficients, were synthesized narratively rather than pooled. These estimates were reviewed according to coefficient type, scale, exposure coding, outcome scale, adjustment status, and direction of effect, with particular attention to whether worse oral status or function corresponded to greater depressive burden. No omnibus coefficient meta-analysis was undertaken because these estimators are not directly interchangeable in scale, interpretation, or underlying model assumptions, and pooling them would therefore have produced a summary measure of doubtful substantive meaning. A structured narrative approach was used instead to preserve analytic transparency while avoiding cross-model aggregation unsupported by metric equivalence [91].

## 3. Results

### 3.1. Study Selection

The database search yielded 2050 records. After removal of 973 duplicates, 1077 records remained for title/abstract screening, of which 1006 were excluded. Seventy–one full–text reports were retrieved and assessed for eligibility, with no reports lost at the retrieval stage. Following full-text assessment, 30 observational studies [92,93,94,95,96,97,98,99,100,101,102,103,104,105,106,107,108,109,110,111,112,113,114,115,116,117,118,119,120,121] were retained in the final review corpus, whereas 41 reports [122,123,124,125,126,127,128,129,130,131,132,133,134,135,136,137,138,139,140,141,142,143,144,145,146,147,148,149,150,151,152,153,154,155,156,157,158,159,160,161,162] were excluded for prespecified reasons. The full pattern of report-level exclusions is presented in Supplementary Materials Table S3. The most frequent reason for exclusion was directionality mismatch/reverse analytic direction, namely studies in which depression or psychological symptoms were modeled as the predictor, grouping variable, or explanatory factor and oral status was modeled as the outcome. Additional reports were excluded because they involved non-depressive or non-standalone depressive outcomes, insufficiently geriatric samples or non-separable older-adult subgroups, ineligible publication types, no eligible oral exposure or an exposure outside the prespecified review scope, an ineligible study design, or insufficient quantitative reporting for reliable eligibility adjudication or effect extraction.

**Figure 1:**
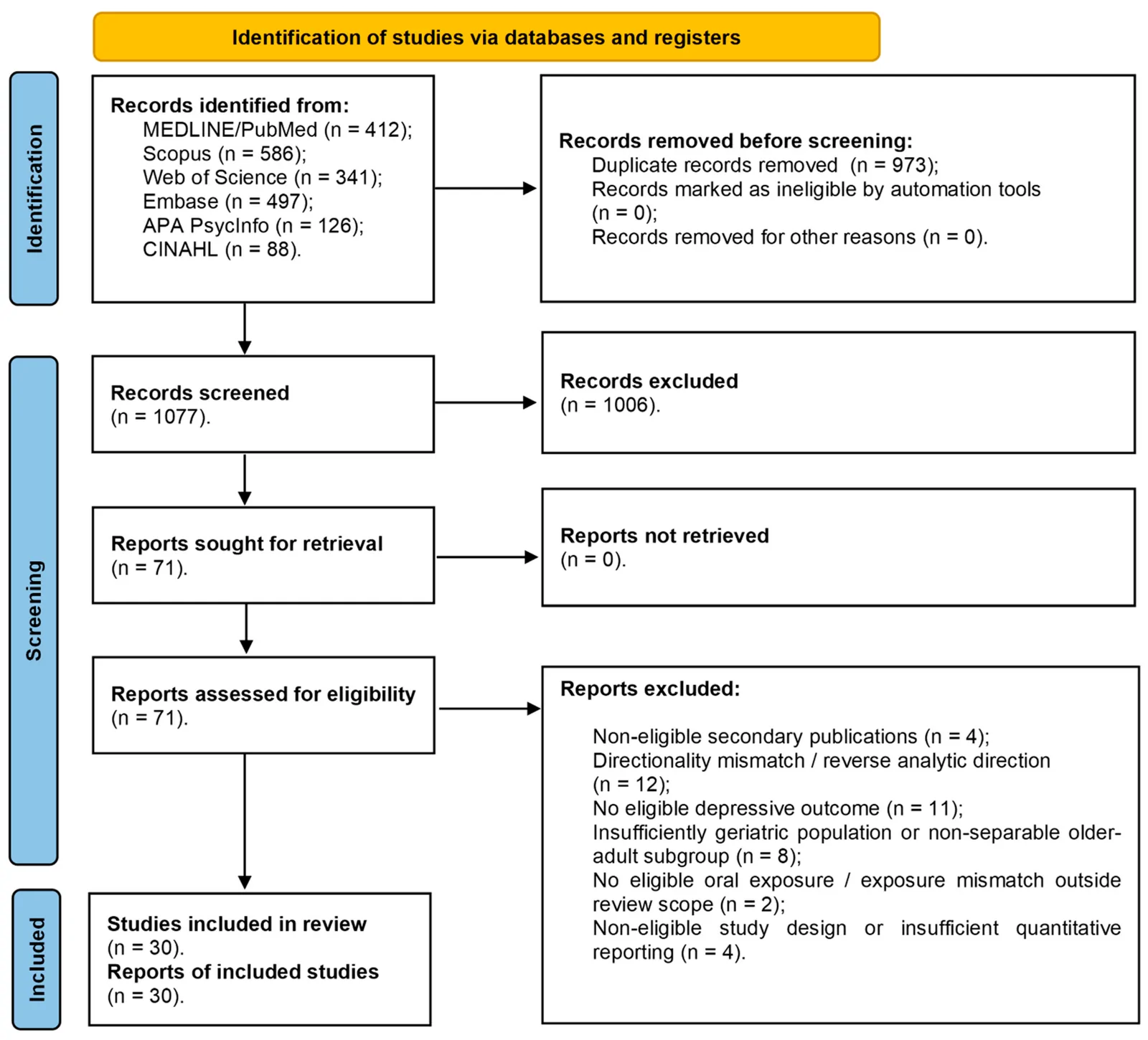
PRISMA 2020 [47] flow diagram of study identification, screening, eligibility assessment, and inclusion. After deduplication, 1077 records were screened, 71 full-text reports were assessed for eligibility, and 30 studies were included in the final review.

Overall, this exclusion profile was consistent with the review’s deliberately narrow analytic framework, which prioritized older-adult observational studies that explicitly evaluated oral structural or functional impairment as the exposure in relation to depressive outcomes as the endpoint. All 30 included studies contributed to the qualitative synthesis, whereas 29 studies [92,93,94,95,96,97,98,99,101,102,103,104,105,106,107,108,109,110,111,112,113,114,115,116,117,118,119,120,121] entered at least one quantitative or structured secondary analytic stream, as reconciled in Supplementary Materials Table S7. One study [100] was retained for qualitative synthesis only and did not contribute to the frozen quantitative workflow. The final evidence base was therefore complete at the report level, internally reconciled across analytic streams, and unaffected by full-text retrieval loss. Study-level contributions to the qualitative and/or quantitative evidence synthesis are summarized in Supplementary Materials Table S2. The study-selection process is shown in Figure 1.

### 3.2. Characteristics of Included Studies and Evidence Mapping

Detailed study-level characteristics are summarized in Supplementary Materials Tables S2 and S4, whereas the final allocation of studies across the frozen analytic streams is reconciled in Tables S7–S10. Across the 30 included observational studies published between 2016 and 2026, 18 studies (60.0%) were cross-sectional or cross-sectional-analytic [92,93,94,95,98,99,100,101,103,106,109,110,111,112,113,116,117,118,120], whereas 12 studies (40.0%) used longitudinal or prospective designs [96,97,102,104,105,107,108,112,114,115,119,121]. The evidence base was therefore weighted toward cross-sectional inference, but it also included a substantial longitudinal component comprising conventional cohort studies, registry-linked follow-up, panel analyses, repeated-wave aging cohorts, and one marginal-structural-model analysis [96,97,102,104,105,107,108,112,114,115,119,121]. Most studies were population–based, community-based, or nationally representative [92,95,98,99,100,101,104,106,107,108,111,115,116,117,118,119,120,121], although the corpus also included institutionalized older adults living in old-age homes [93], a frailty outpatient clinic sample [94], and a registry-based older-adult cohort [102]. Reported study sizes ranged from 201 to 169,061 participants. However, several reports used subgroup-restricted, follow-up, imputed, or repeated-wave analytic samples that were smaller than the parent cohort or broader than the source population. Most studies were conducted in strictly older-adult cohorts or clearly separable older-adult analytic subsamples. Only two reports relied on broader source populations while still contributing an eligible older-adult estimate to the review: the multi-country Survey of Health, Aging and Retirement in Europe (SHARE) analysis beginning at age 50 years [92] and the Korean nationally representative survey study with a separately reported subgroup aged ≥65 years [95].

The geographic distribution of the evidence was concentrated in Asia. Twenty-one of the 30 studies (70.0%) were conducted in Asian settings [93,94,95,96,97,98,99,102,104,105,106,107,110,111,112,113,114,115,116,119,120,121], including eight from Japan [94,97,102,104,105,113,114,119], six from China [99,110,111,116,120,121], and four from South Korea [95,106,107,112]. The remaining nine studies (30.0%) came from multinational Europe/Israel [92], Brazil [96,103], Australia [100,118], the United States [101,117], the United Kingdom [108], and Poland [109]. This distribution indicates that the available evidence was predominantly Asian, with more limited representation from Europe, the Americas, and Oceania. Where sex distribution was reported, women generally predominated across the included cohorts [92,93,94,95,96,97,98,99,101,102,103,104,105,107,108,109,110,111,112,114,115,116,119,120,121], although a small number of studies were male-predominant [113,117] and one Australian cohort included men only [118]. The age profile was consistently geriatric, with most mean ages clustered in the late sixties to early eighties and several studies focusing on particularly old community-dwelling populations [94,99,100,120,121].

Exposure definitions were broad enough to capture the multidimensional nature of oral impairment while remaining structured for evidence mapping. The most common domain was tooth loss/dentition [92,93,94,95,96,97,101,102,103,104,109,116,119,120,121], followed by oral frailty/functional decline [94,98,111,114], chewing dysfunction or chewing disability [102,106,115,118], broader oral-impairment constructs such as self–rated oral health, oral physical function, or functional tooth units [107,110,112,117], OHRQoL/oral impacts [105,108], denture/prosthetic status [99,104], and xerostomia/salivary dysfunction [113]. This distribution is clinically informative, but it also reinforces that not all retained oral indicators are equivalent in construct specificity or causal proximity to depressive outcomes.

The final evidence map confirmed that all 30 studies contributed to the qualitative synthesis, whereas 29 studies (96.7%) contributed to at least one quantitative or structured secondary synthesis. The primary OR-only meta-analysis included 15 studies (50.0%) [94,95,97,98,102,103,105,106,107,108,111,113,116,117,119]. A secondary RR/PR stream comprised four studies (13.3%) [96,104,115,118], and a secondary beta/coefficient stream comprised 10 studies (33.3%) [92,93,99,101,109,110,112,114,120,121]. One study remained qualitative only (3.3%) [100]. This stream-based architecture indicates that the review corpus was quantitatively informative for most included reports, but only after strict metric separation and prespecified evidence mapping. Accordingly, studies reporting ORs formed the primary quantitative analysis, whereas ratio-based and coefficient-based studies were retained in structured secondary syntheses consistent with their statistical meaning and degree of comparability. Overall, the evidence base was characterized by substantial heterogeneity in exposure definitions and outcome operationalization, with a predominance of cross-sectional designs and Asian populations, which may limit generalizability.

### 3.3. Risk of Bias and Certainty of Evidence

Study-level methodological appraisals and body-level certainty judgments are summarized in Supplementary Materials Table S5, with Newcastle–Ottawa Scale (NOS) assessments reported in Panel A and GRADE judgments reported in Panel B. Across the 30 included observational studies, total NOS scores ranged from 5 to 9, with a mean of 7.3, a median of 7.0, and an interquartile range of 7.0–8.0. Using the prespecified study-level classification, 25 studies (83.3%) were rated as high methodological quality [92,95,96,97,98,99,101,102,104,105,106,107,108,110,111,112,113,114,115,116,117,118,119,120,121], whereas 5 studies (16.7%) were rated as moderate methodological quality [93,94,100,103,109]. No study fell within the low-quality range. Overall, this distribution indicates that the included evidence base was methodologically suitable for synthesis, with most NOS deductions reflecting limitations commonly encountered in observational studies rather than major deficiencies in exposure ascertainment, outcome definition, or analytical reporting.

At the domain level, the strongest NOS performance was observed in comparability, where multivariable adjustment for key sociodemographic and clinical covariates was commonly reported across the corpus [92,94,95,96,97,98,99,101,102,103,104,105,106,107,108,110,111,112,113,114,115,116,117,118,119,120,121]. By contrast, deductions in the selection and outcome/exposure domains were more often related to cross-sectional temporality, setting-restricted recruitment, self-reported oral-exposure assessment, subgroup-derived older-adult estimates from broader sampling frames, or incomplete reporting of participation and follow-up structure [92,93,94,95,98,99,100,103,105,106,107,108,109,110,111,112,113,116,117,118,120]. Accordingly, the NOS profile suggests that most studies were interpretable within the limits of observational design, but were not uniformly protected against residual confounding, indirectness, or temporal ambiguity.

Body-level certainty judgments were more conservative than the study-level NOS profile because GRADE evaluates confidence in a synthesized body of evidence rather than study quality alone. For the primary OR-only evidence, the observational evidence initially entered the GRADE assessment at low certainty. Confidence was further reduced by serious inconsistency, reflected by substantial between-study heterogeneity (I^2^ = 88.1%) and a 95% prediction interval extending across the null (0.849–3.065), indicating that the magnitude of association varied markedly across study contexts. Additional concern arose from possible publication bias or small-study effects: funnel-plot asymmetry was supported by the Egger test, and the trim-and-fill sensitivity analysis attenuated the pooled estimate from OR = 1.613 to OR = 1.290 (95% CI: 0.990–1.690), with the interval crossing the null. Residual confounding, variation in exposure and depression ascertainment, and incomplete temporal separation in the substantial cross-sectional component of the evidence base provided further reasons for caution. No upgrading factor was judged sufficiently strong or consistent to offset these limitations. Accordingly, the primary OR-only evidence was rated as very low certainty. The secondary RR/PR evidence was also considered very low certainty because of the small number of studies, mixed ratio estimands, indirectness, and residual confounding, while the coefficient-based stream remained very low certainty because of heterogeneous coefficient types, model structures, limited cross-study comparability, and imprecision. These ratings indicate substantial uncertainty regarding the precise magnitude and generalizability of the observed associations and therefore support cautious, non-causal interpretation.

### 3.4. Qualitative Synthesis of Observational Evidence

Because the included studies differed substantially in exposure definition, outcome format, temporality, and statistical metric, the qualitative synthesis was organized according to prespecified oral-exposure domains rather than by study design alone. All 30 included studies contributed to this synthesis [92,93,94,95,96,97,98,99,100,101,102,103,104,105,106,107,108,109,110,111,112,113,114,115,116,117,118,119,120,121]. The narrative synthesis is presented in three domain-based subsections: structural oral impairment; functional oral impairment; and OHRQoL, xerostomia, prosthetic status, and other oral-impairment indicators. This structure was intended to complement, rather than duplicate, the subsequent quantitative analyses.

#### 3.4.1. Structural Oral Impairment and Depressive Outcomes

Structural oral impairment was one of the most consistently represented exposure domains in the included literature. In total, 12 of the 30 included studies examined dentition-related structural indicators, including missing natural teeth, number of remaining teeth, extracted-tooth burden, severe tooth loss, and edentulism [92,93,95,96,97,101,103,109,116,119,120,121]. Across this structural subset, the qualitative signal was predominantly convergent: 10 studies reported findings directionally compatible with greater depressive burden among participants with poorer dentition or greater tooth loss [92,93,95,96,97,109,116,119,120,121], whereas 2 studies showed attenuated, uncertain, or non–independent associations after adjustment or subgroup restriction [101,103]. This pattern indicates that dentition–related deficits were repeatedly linked to depressive outcomes across diverse settings, although the magnitude and statistical independence of the association were not fully uniform across all structural contrasts [92,93,95,96,97,101,103,109,116,119,120,121].

The cross-sectional structural evidence was broadly consistent in direction. In the multinational SHARE analysis, missing natural teeth and a greater number of missing teeth were both associated with higher depressive symptom burden, whereas more complete tooth replacement was associated with lower depressive symptoms [92]. In institutionalized older adults in India, higher oral-disease burden, including dentition-related impairment, was associated with greater depression severity [93]. In the older-adult subgroup of a nationally representative Korean survey, severe tooth loss was associated with depressive symptoms [95]. A clinically assessed Polish study likewise found that extracted-tooth burden was independently associated with greater depression severity [109]. In a large Chinese population-based study, Wang et al. reported that stage-specific tooth loss was associated with depressive symptoms, with the strongest adjusted association observed in the 20–27 tooth-loss category [116]. In another Chinese population-based study, poorer dentition, reflected by fewer remaining teeth, was associated with higher depression levels, with dietary diversity and life satisfaction acting as partial explanatory pathways [120]. Taken together, these studies support a recurrent cross-sectional gradient in which more advanced structural oral deterioration corresponds to greater depressive burden [92,93,95,109,116,120].

Not all structural findings remained clearly independent after full modeling. In the Brazilian study by Muniz et al., depressive symptoms were not independently associated with normative oral conditions, including edentulism, after multivariable adjustment [103]. Likewise, the instrumental-variable analysis by Matsuyama et al. yielded an imprecise older-adult subgroup estimate compatible with no clear independent association [101]. These studies help temper selective-significance bias in the narrative. Even so, the longitudinal structural evidence showed an overall adverse association, suggesting that poorer dentition may precede less favorable depressive outcomes in some older-adult settings [96,97,119,121].

Overall, the qualitative evidence indicates that structural oral impairment, especially tooth loss, severe dentition loss, and edentulism, was usually associated with more adverse depressive outcomes in older adults, although heterogeneity remained at the level of contrast definition, analytic model, and population frame. The most consistent patterns were observed in studies evaluating cumulative or more advanced dentition loss, whereas null or attenuated findings were confined to a smaller subset of analyses involving broader normative oral constructs or less precise subgroup-specific estimates [92,93,95,96,97,101,103,109,116,119,120,121]. In substantive terms, the structural evidence supports the view that dentition-related deterioration is an important component of the oral–depression relationship in later life and provides a qualitatively coherent foundation for the quantitative analyses presented in the following sections.

#### 3.4.2. Functional Oral Impairment and Depressive Outcomes

Functional oral impairment formed a second major qualitative domain and was represented by 8 of the 30 included studies (26.7%) [94,98,102,106,111,114,115,118]. This subset comprised 5 cross-sectional studies [94,98,106,111,118] and 3 longitudinal studies [102,114,115] and covered two closely related constructs: oral frailty/functional decline [94,98,111,114] and chewing dysfunction or chewing disability [102,106,115,118]. In contrast to the somewhat more heterogeneous structural-dentition literature, the functional evidence was notably uniform in direction. All 8 studies (100.0%) reported findings consistent with greater depressive burden among participants with poorer oral function, whether oral impairment was operationalized as oral frailty, oral function decline, poor chewing ability, chewing disability, or limited chewing capacity [94,98,102,106,111,114,115,118].

The cross-sectional oral-frailty evidence was strongly concordant. In a frailty outpatient sample in Japan, Kawamura et al. reported that oral frailty was associated with depressive mood in older adults, supporting the view that multidimensional oral-function loss clusters with affective vulnerability in clinically burdened aging populations [94]. In Taiwan, Lin et al. likewise found that oral frailty was associated with late-life depression in community-dwelling older adults [98]. In China, Sun et al. showed that oral frailty remained independently associated with depressive symptoms even in the presence of social frailty and sarcopenia, suggesting that oral frailty contributes additional explanatory information rather than merely reflecting generalized age-related decline [111]. Taken together, these studies move the functional literature beyond single indicators such as missing teeth or chewing difficulty and instead conceptualize oral impairment as a multidimensional decline state involving mastication, swallowing, dryness, pronunciation, or oral motor performance [94,98,111].

The chewing-function literature was similarly consistent. In South Korea, Park et al. reported that subjective chewing difficulty was associated with depressive symptoms in a large community-based older-adult sample [106]. In Australia, Wright et al. found that limited chewing capacity was associated with depressive symptoms in older men, even within a cohort framework that also captured other oral conditions [118]. In Japan, Miyano et al. reported that poor chewing ability was prospectively associated with newly diagnosed depression in later life [102], whereas in Singapore, Tay et al. reported that chewing disability was prospectively associated with a higher risk of clinically significant depressive symptoms over follow-up, with loneliness accounting for part of the observed association [115]. Collectively, these studies indicate that compromised masticatory function is not merely a concurrent marker of poor oral status, but may also be linked to later depressive burden through socially and behaviorally meaningful pathways [102,106,115,118].

The strongest temporal support within the functional domain came from the longitudinal evidence. Miyano et al. provided prospective support for an association between poor chewing ability and later depression diagnosis [102]. Tanaka et al., using a 12–year community-based cohort in Japan, showed that baseline oral frailty was associated with persistently worse depressive-symptom trajectories over time [114]. Tay et al. extended this pattern by showing that chewing disability was prospectively associated with later clinically significant depressive symptoms in a longitudinal marginal structural model, with loneliness acting as an important mediator [115]. These studies are particularly informative because they reduce the interpretive limitations inherent in cross-sectional designs and suggest that functional oral impairment may precede worsening depressive status rather than simply co-occur with it [102,114,115].

Overall, the qualitative evidence indicates that functional oral impairment was one of the most coherent adverse exposure domains in the review corpus. Unlike the structural domain, where some adjusted or subgroup-restricted analyses were attenuated, the functional literature showed a uniformly adverse direction of association across oral frailty, oral function decline, chewing difficulty, and chewing disability [94,98,102,106,111,114,115,118]. In substantive terms, this pattern suggests that the depression-related relevance of oral impairment in older adults is not limited to cumulative structural loss, but extends to day-to-day oral performance and function, which may influence mood through nutritional restriction, effortful eating, social embarrassment, frailty-linked vulnerability, and reduced participation in everyday life.

#### 3.4.3. OHRQoL, Xerostomia, Prosthetic Status, and Other Oral-Impairment Indicators

The remaining qualitative evidence comprised a heterogeneous but clinically important group of oral exposures that did not fit neatly within the structural-dentition or core functional-frailty domains. In total, 10 of the 30 included studies (33.3%) contributed to this domain [99,100,104,105,107,108,110,112,113,117]. These studies covered oral health-related quality of life (OHRQoL) and oral impacts [105,108], xerostomia or salivary dysfunction [113], prosthetic status [99,104], and a broader set of other oral-impairment indicators, including oral physical function, self-rated oral health, global oral health scores, functional tooth units, oral appearance, and meal-related oral dissatisfaction [100,107,110,112,117]. Although these constructs were more heterogeneous than those in the preceding subsections, the qualitative pattern remained directionally coherent: all 10 studies supported an adverse oral-health gradient in which poorer oral status, worse oral impacts, lower prosthetic restoration, or more impaired oral-function proxies were associated with greater depressive burden or less favorable depressive trajectories [99,100,104,105,107,108,110,112,113,117].

The clearest evidence within this domain came from studies of OHRQoL and oral impacts. In Japan, Ohi et al. reported that impaired OHRQoL, measured with the Oral Impacts on Daily Performances (OIDP) instrument, was associated cross-sectionally with depressive symptoms and was also prospectively associated with incident depressive symptoms over 4 years after adjustment for objective oral status, oral-health behavior, cognition, and major clinical covariates [105]. In England, Rouxel et al. showed that worsening oral impacts on daily life were associated with increased depressive symptoms over time, even after accounting for time-varying health and social factors [108]. Although measured differently and in distinct populations, these studies converged on the view that oral impairment becomes especially relevant to depression when it translates into daily-life interference, discomfort, social limitation, or reduced oral quality of life [105,108].

The evidence on prosthetic status was likewise directionally consistent and clinically informative. In the Chinese Longitudinal Healthy Longevity Survey (CLHLS) analysis by Lou et al., denture use was associated with lower depressive symptoms, and part of this relationship appeared to operate through greater dietary diversity [99]. In the Japanese cohort study by Nakazawa et al., the highest risk of incident depressive symptoms was observed among older adults with 0–9 teeth and no dental prostheses, whereas the corresponding risk was attenuated among those with equally severe tooth loss but with prosthetic restoration [104]. Taken together, these findings suggest that prosthetic rehabilitation may partially buffer the affective consequences of severe dentition loss, although the magnitude of this mitigation is likely shaped by nutrition, function, adaptation, and broader social context [99,104].

Other oral-impairment indicators also showed predominantly adverse associations with depressive outcomes. In South Korea, Park et al. found that worse oral physical function was associated with depressive symptoms at follow-up [107]. In urban China, Sun et al. reported that poorer self-rated oral health was associated with greater depressive symptoms and that this relationship was partly mediated by dietary satisfaction [110]. In the Korean Longitudinal Study of Aging (KLoSA), Sung et al. showed that poorer oral health was prospectively associated with later depressive symptoms, with formal social engagement partially mediating the eligible oral-to-depression pathway, although the reverse depression-to-oral-health pathway was stronger overall [112]. In the U.S. National Health and Nutrition Examination Survey (NHANES)-based study by Wei et al., severely reduced functional tooth units were associated with higher odds of depression, supporting the relevance of broader oral-function proxies even when they are not classified under the core chewing-dysfunction domain in the frozen dataset [117]. Finally, the qualitative-only study by Mariño et al. showed that dentition status, concern about mouth appearance, and enjoyment of meals contributed significantly to depressive symptoms and psychological well-being in independent-living older Australians, underscoring the relevance of appearance-and satisfaction-related oral constructs that are clinically meaningful but not easily harmonized into a pooled quantitative framework [100].

Xerostomia and salivary dysfunction also emerged as relevant depressive correlates. In Japanese community-dwelling older adults, Takiguchi et al. found that low unstimulated salivary flow rate and other oral-dysfunction indicators, including mouth pain, were associated with a high depressive-symptom burden [113]. This finding is important because it extends the oral–depression relationship beyond visible dentition loss and chewing impairment to include oral dryness and salivary insufficiency, domains that may influence mood through discomfort, altered eating, oral pain, sleep disturbance, and reduced oral confidence [113]. Collectively, this heterogeneous but directionally consistent domain suggests that the oral–depression relationship in later life is not confined to tooth counts and chewing performance alone. Depressive outcomes were also linked to oral impacts on daily life, impaired OHRQoL, salivary dysfunction, lack of prosthetic restoration in the context of severe tooth loss, reduced global oral function, poorer self-rated oral health, and appearance-related oral concerns [99,100,104,105,107,108,110,112,113,117]. These findings reinforce the broader interpretation that clinically meaningful oral impairment in older adults is multidimensional and that depression-related vulnerability may be shaped not only by structural loss and mastication, but also by discomfort, oral self-perception, social confidence, daily oral functioning, and the extent to which oral deficits remain unmitigated by adaptive or restorative pathways.

### 3.5. Primary OR-Only Random-Effects Meta-Analysis

**Figure 2:**
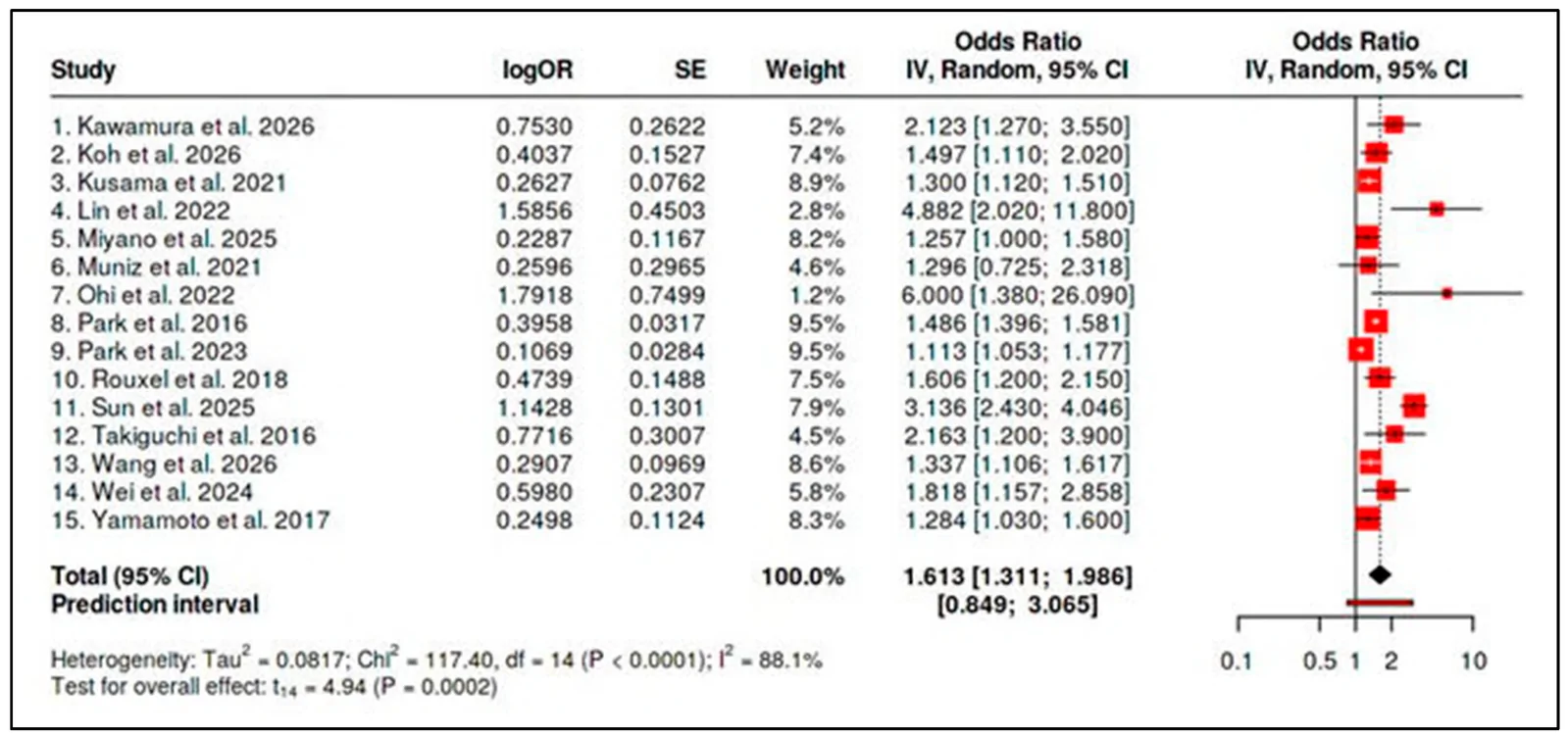
Forest plot of the 15 studies included in the primary OR-only meta-analysis. The pooled random-effects estimate was OR = 1.613 (95% CI: 1.311–1.986), with substantial between-study heterogeneity (I^2^ = 88.1%) and a 95% prediction interval of 0.849–3.065. Squares represent study-specific estimates, horizontal lines represent 95% CIs, and the diamond represents the pooled estimate [94,95,97,98,102,103,105,106,107,108,111,113,116,117,119].

The primary OR-only meta-analysis was based on the frozen effect-selection dataset and included 15 of the 30 studies (50.0%) retained in the final review corpus and 15 of the 29 studies (51.7%) contributing to at least one quantitative or structured secondary synthesis [94,95,97,98,102,103,105,106,107,108,111,113,116,117,119]. This analysis comprised 9 cross-sectional studies [94,95,98,103,106,111,113,116,117] and 6 longitudinal studies [97,102,105,107,108,119] and covered multiple oral–exposure families, including tooth loss/dentition [95,97,103,116,119], oral frailty/functional decline [94,98,111], chewing dysfunction [102,106], oral health-related quality of life (OHRQoL)/oral impacts [105,108], xerostomia/salivary dysfunction [113], and other oral impairment [107,117]. Most studies in this analysis were classified as having high methodological quality, with only two classified as having moderate or lower methodological quality [94,95,97,98,102,103,105,106,107,108,111,113,116,117,119]. The primary pooled results are summarized in Table 1 and presented in Figure 2.

Across the 15 OR-based studies, the random-effects model showed a statistically significant adverse association between oral structural and functional impairment and depressive outcomes in older adults, with a pooled odds ratio (OR) of 1.613 (95% CI: 1.311–1.986; *p* = 0.0002) [94,95,97,98,102,103,105,106,107,108,111,113,116,117,119]. All study-specific point estimates indicated an adverse association, ranging from OR = 1.113 [107] to OR = 6.000 [105]. Between-study heterogeneity was substantial (Cochran’s Q = 117.40, df = 14, *p* < 0.0001; I^2^ = 88.1%, 95% CI: 82.0–92.1%), with τ^2^ = 0.0817 (95% CI: 0.0360–0.4410), τ = 0.2860 (95% CI: 0.1910–0.6640), and Higgins’ H = 2.896 (95% CI: 2.357–3.557) [94,95,97,98,102,103,105,106,107,108,111,113,116,117,119]. The corresponding 95% prediction interval ranged from 0.849 to 3.065, indicating that the direction and magnitude of association may vary across comparable settings and that the pooled OR should therefore be interpreted as an average association across heterogeneous study contexts rather than as a uniform effect applicable across settings. This pattern is consistent with the clinical and methodological diversity of the primary OR-only meta-analysis, which remained metric-compatible but still incorporated variation in exposure family, outcome formulation, ascertainment type, and temporal design [94,95,97,98,102,103,105,106,107,108,111,113,116,117,119].

Because these oral-exposure constructs are clinically related but not exchangeable, the primary OR-only synthesis is also presented by exposure family in Table 1, Panel B. Tooth loss/dentition showed the most stable construct-specific association (k = 5; OR = 1.326, 95% CI: 1.247–1.409; I^2^ = 0.0%), whereas oral frailty/functional decline showed a larger pooled association (k = 3; OR = 2.967, 95% CI: 1.459–6.037; I^2^ = 33.8%). Chewing dysfunction (k = 2; OR = 1.419, 95% CI: 0.550–3.659; I^2^ = 47.6%), OHRQoL/oral impacts (k = 2; OR = 2.529, 95% CI: 0.001–7225.535; I^2^ = 66.3%), and other oral impairment (k = 2; OR = 1.349, 95% CI: 0.064–28.347; I^2^ = 77.6%) were substantially less precise because of sparse study counts. Xerostomia/salivary dysfunction was represented by a single OR-eligible study and was therefore not pooled as a separate subgroup. Accordingly, construct-specific estimates should be used preferentially when interpreting the magnitude associated with a particular oral domain, while the overall OR = 1.613 is retained only as a descriptive cross-domain summary.

The principal quantitative findings can be summarized in three observations. First, all 15 study-specific OR estimates indicated an adverse association, ranging from 1.113 to 6.000, with an overall pooled OR of 1.613 (95% CI: 1.311–1.986). Second, the substantial heterogeneity (I^2^ = 88.1%) and the wide 95% prediction interval (0.849–3.065), which crossed the null, indicate considerable variation across study contexts. Third, although no individual study determined the overall direction of the pooled estimate, influence diagnostics identified several studies that contributed disproportionately to heterogeneity, and the trim-and-fill sensitivity analysis materially attenuated the pooled association. Table 1 and the four graphical displays presented in the following sections provide complementary perspectives on these findings and should be interpreted together.

Taken together, the primary OR-only synthesis provides the central quantitative signal of the review: poorer oral structural and functional status was associated, on average, with higher odds of depression-related outcomes in older adults. At the same time, the heterogeneity profile and wide prediction interval indicate meaningful between-study variation, and the main pooled estimate should be interpreted as associative rather than causal (Figure 2).

The cross-domain pooled OR was therefore not considered sufficient as the sole quantitative basis for interpretation. Complementary stratified analyses were used to examine whether the average association varied according to study design, oral–exposure family, ascertainment type, geographic region, methodological quality, and older-adult population frame, with corresponding exploratory meta-regression where estimable. Among these dimensions, study design, exposure family, and ascertainment type were given particular interpretative emphasis because they directly address temporality, construct definition, and measurement approach. These additional analyses are presented in Section 3.6 and Section 3.7 and in Supplementary Tables S12–S14 and should be interpreted jointly with, rather than as ancillary checks of, the overall pooled OR. However, because several strata contained only two or three studies, these analyses remain exploratory and were not reclassified as independent confirmatory or co-primary tests.

### 3.6. Exploratory Subgroup Analyses

Exploratory subgroup analyses of the primary OR-only meta-analysis are summarized in Supplementary Materials Table S12. Across analyzable strata, the associations remained in the same adverse direction, although their magnitude and precision varied and several strata contained only two or three studies. Cross-sectional studies showed a larger pooled association (k = 9; OR = 1.862, 95% CI: 1.385–2.502) than longitudinal studies (k = 6; OR = 1.266, 95% CI: 1.047–1.531), although this descriptive contrast should not be interpreted as definitive effect modification.

Exposure-family analyses were particularly informative. Oral frailty/functional decline showed the largest pooled estimate (k = 3; OR = 2.967, 95% CI: 1.459–6.037), whereas tooth loss/dentition showed a smaller but highly consistent association (k = 5; OR = 1.326, 95% CI: 1.247–1.409; I^2^ = 0.0%). Chewing dysfunction, OHRQoL/oral impacts, and other oral-impairment strata showed associations in the same adverse direction, but the small numbers of studies and wide confidence intervals precluded reliable subgroup inference.

Estimates were similar in Asian and non-Asian strata, although the latter contained only three studies, limiting geographic comparisons. Importantly, 12 of the 15 studies included in the primary OR-only meta-analysis were conducted in Asian settings, and the Asian subgroup remained highly heterogeneous (OR = 1.651, 95% CI: 1.257–2.168; I^2^ = 90.3%), whereas the non-Asian subgroup comprised only three studies (OR = 1.604, 95% CI: 1.170–2.200; I^2^ = 0.0%). The numerical similarity between these subgroup estimates should therefore not be interpreted as evidence of geographic equivalence or broad geographic generalizability, because the non-Asian comparison was sparse and the Asian evidence itself was internally heterogeneous. Associations also tended to be larger in studies using self-reported or mixed ascertainment than in those using clinical/objective or structured ascertainment. The high-quality NOS subgroup closely reproduced the overall estimate, whereas the moderate-or-lower quality subgroup contained only two studies and was highly imprecise. Overall, these exploratory analyses suggest that the magnitude and coherence of the association vary particularly by oral-exposure construct and ascertainment method; complete subgroup estimates and heterogeneity statistics are provided in Table S12.

### 3.7. Exploratory Meta-Regression Findings

Exploratory univariate mixed-effects meta-regression results for the primary OR-only meta-analysis are summarized in Supplementary Materials Table S13, with coefficient-level estimates reported in Table S14. Because only 15 studies contributed to the primary meta-analysis and several moderator levels were sparse, these analyses were considered exploratory and were not interpreted as confirmatory tests of effect modification. Exposure family (QM = 60.8724, *p* < 0.0001) and ascertainment type (QM = 9.0584, *p* = 0.0108) showed statistically significant omnibus moderator tests, suggesting that part of the observed heterogeneity was structured by what oral construct was measured and how it was ascertained. Design class approached conventional significance (QM = 3.4177, *p* = 0.0645), whereas region did not explain detectable variation (QM = 0.0134, *p* = 0.9079).

Methodological quality was examined beyond categorical subgrouping using two separate meta-regression parameterizations. The categorical NOS quality moderator did not show evidence of association with effect magnitude (QM = 0.0198, *p* = 0.8882). Consistently, modeling the NOS total score as a continuous moderator also showed no statistically detectable quality-effect gradient (QM = 0.8834, *p* = 0.3473); the coefficient for each one-point increase in NOS score was −0.0998 on the log-OR scale (*p* = 0.3644; exponentiated coefficient = 0.9050, 95% CI: 0.7196–1.1383). These findings do not demonstrate that methodological quality is unrelated to effect size; rather, with only 15 studies, a restricted NOS score range, and the known limitations of summary quality scores, the analysis had limited ability to detect such a relationship.

Overall, the meta-regression findings suggest that heterogeneity was more visibly related to oral-construct definition and ascertainment than to geography or NOS-based study quality. However, these study-level moderator results remain exploratory and cannot distinguish true effect modification from construct heterogeneity, residual confounding, or methodological artifact.

### 3.8. Leave-One-Out, Influence Diagnostics, and Baujat Assessment

**Figure 3:**
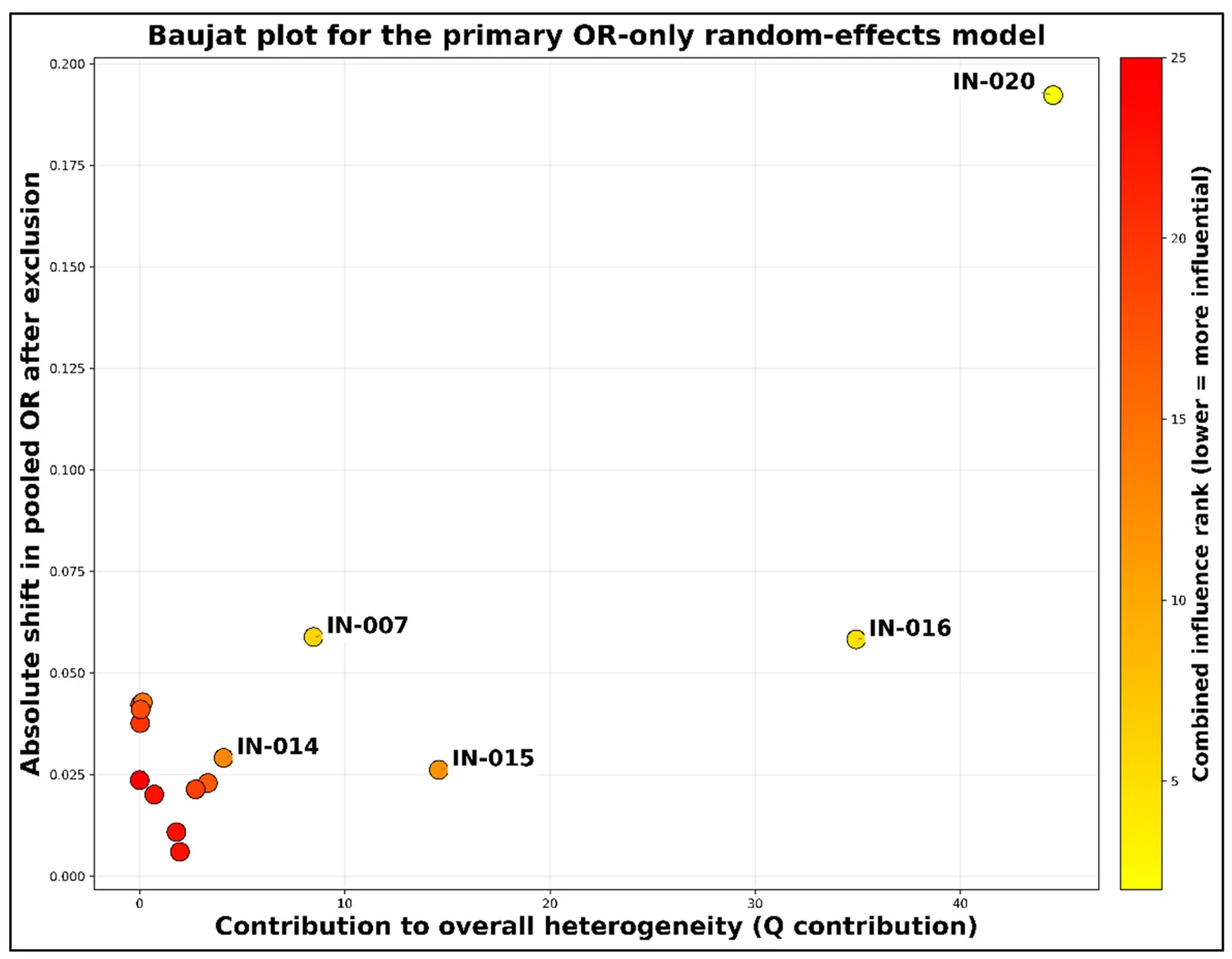
Baujat plot showing each study’s contribution to between-study heterogeneity and the change in the pooled OR after study exclusion. Park et al. [107] contributed most strongly to heterogeneity, whereas Sun et al. [111] produced the largest change in the pooled estimate. No individual study determined the overall direction of the pooled association; complete numerical diagnostics are provided in Supplementary Tables S15 and S16 [94,95,97,98,102,103,105,106,107,108,111,113,116,117,119].

Leave-one-out, influence, and Baujat diagnostics are reported in Supplementary Materials Tables S11, S15 and S16, with the Baujat pattern visualized in Figure 3. The pooled OR remained above the null and statistically significant after exclusion of each study in turn, ranging from OR = 1.421 (95% CI: 1.231–1.640) after exclusion of Sun et al. [111] to OR = 1.671 (95% CI: 1.350–2.069) after exclusion of Park et al. [107]. Thus, the primary pooled association was not dependent on any single study, although heterogeneity remained substantial across most exclusions. Influence diagnostics identified Lin et al. [98], Park et al. [107], and Sun et al. [111] as the three studies with the greatest combined influence in the Baujat assessment (Supplementary Table S16). Their influence arose through different combinations of contribution to heterogeneity and displacement of the pooled estimate: Park et al. [107] contributed particularly strongly to heterogeneity, whereas Sun et al. [111] produced the largest absolute shift in the pooled OR and was the only study exceeding the prespecified Cook’s distance threshold. Importantly, however, sequential exclusion of individual studies did not reverse the direction or eliminate the statistical significance of the pooled association. Thus, Figure 3 should be interpreted as identifying an uneven distribution of influence across the evidence base rather than a single study responsible for the overall adverse association [94,95,97,98,102,103,105,106,107,108,111,113,116,117,119].

### 3.9. Small-Study Effects and Trim-and-Fill Sensitivity Analysis

**Figure 4:**
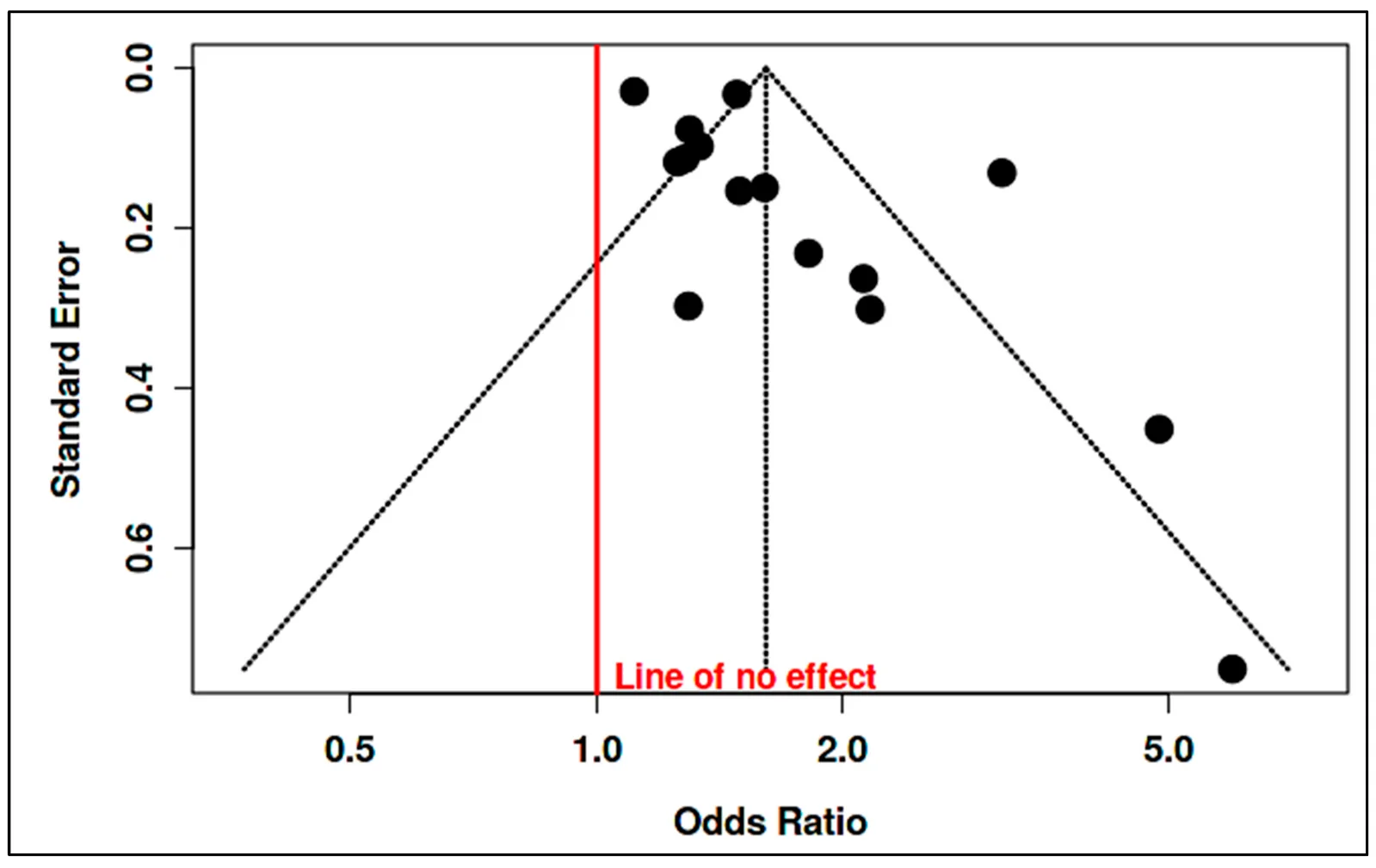
Funnel plot for the 15 studies included in the primary OR-only meta-analysis. Funnel-plot asymmetry was supported by Egger’s regression test (intercept = 2.13, 95% CI: 0.31–3.95; *p* = 0.039). Because heterogeneity was substantial (I^2^ = 88.1%), the observed asymmetry should be interpreted as evidence compatible with small-study effects rather than as definitive evidence of publication bias. Black dots represent individual study estimates; the vertical dashed line indicates the pooled effect estimate, and the diagonal dashed lines indicate the pseudo 95% confidence limits [94,95,97,98,102,103,105,106,107,108,111,113,116,117,119].

Funnel-plot inspection suggested asymmetry in the primary OR-only analysis (Figure 4), supported by Egger’s regression test (intercept = 2.13, 95% CI: 0.31–3.95; *p* = 0.039). This finding is compatible with small-study effects but should not be interpreted as definitive evidence of publication bias, particularly given the substantial between-study heterogeneity [94,95,97,98,102,103,105,106,107,108,111,113,116,117,119].

**Figure 5:**
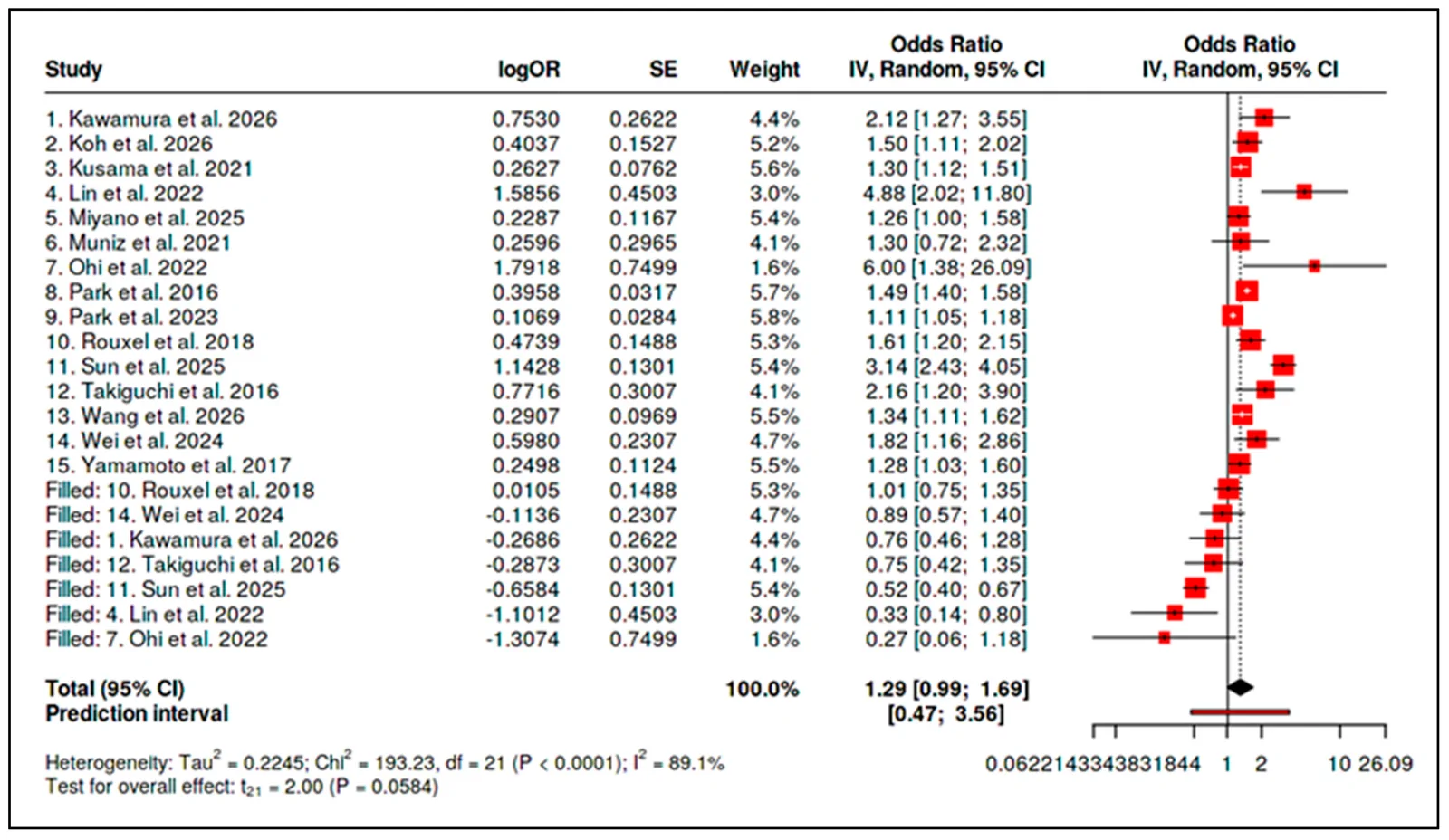
Trim-and-fill sensitivity analysis of the primary OR-only meta-analysis. Seven potentially missing studies were imputed under the funnel-asymmetry model. The pooled estimate decreased from OR = 1.613 (95% CI: 1.311–1.986) to OR = 1.290 (95% CI: 0.990–1.690; *p* = 0.0584) and no longer reached conventional statistical significance. The analysis represents an assumption-dependent sensitivity scenario rather than a corrected estimate of the true association [94,95,97,98,102,103,105,106,107,108,111,113,116,117,119].

Trim-and-fill sensitivity analysis imputed seven potentially missing studies (Table 2; Figure 5). The adjusted pooled estimate was attenuated to OR = 1.290 (95% CI: 0.990–1.690; *p* = 0.0584) and therefore no longer reached conventional statistical significance. Heterogeneity remained substantial (I^2^ = 89.1%), with a wide 95% prediction interval (0.470–3.560). Thus, adjustment for potential asymmetry weakened the statistical robustness of the pooled signal without producing a more homogeneous effect structure.

Overall, the small-study-effect analyses materially qualify the primary pooled result. Funnel-plot asymmetry and the significant Egger test indicate that smaller studies tended to contribute to an asymmetric effect distribution, although substantial heterogeneity prevents attributing this pattern specifically to publication bias. Under the trim-and-fill sensitivity scenario, the pooled estimate decreased from OR = 1.613 to OR = 1.290 and its 95% CI crossed the null (0.990–1.690; *p* = 0.0584). The trim-and-fill estimate should not be interpreted as a corrected true effect; rather, the contrast between the observed and imputed-data models demonstrates that the magnitude and conventional statistical significance of the pooled association are sensitive to assumptions about potentially missing small studies.

### 3.10. Secondary Ratio-Based Evidence (RR/PR): Structured Narrative Synthesis

Secondary non-OR-based evidence is summarized in Supplementary Materials Table S9. This stream comprised 4 studies (13.3% of the full review corpus), including 2 risk-ratio studies [104,115] and 2 prevalence-ratio studies [96,118]. Three of these studies were longitudinal [96,104,115], whereas one was cross-sectional [118]. All four studies were classified as high methodological quality on the Newcastle–Ottawa Scale and all four reported adjusted estimates, but they were not pooled meta-analytically because this stream combined prevalence-based and risk-based relative measures, included both cross-sectional and longitudinal evidence, and remained too small to support a stable secondary ratio meta-analysis without introducing additional interpretive indirectness. These studies were therefore retained for structured quantitative narrative synthesis rather than formal pooled modeling.

Despite these constraints, the direction of effect across the RR/PR stream was fully consistent. All 4 studies (100.0%) supported an adverse association between poorer oral status or function and depressive outcomes in older adults [96,104,115,118]. In the Brazilian longitudinal cohort studied by Kunrath and Silva [96], incident tooth loss between 2009 and 2015 was associated with a higher prevalence of depressive moods at follow-up (PR = 1.860, 95% CI: 1.010–3.530). In the Japanese cohort analyzed by Nakazawa et al. [104], older adults with 0–9 remaining teeth and no dental prostheses had a higher risk of incident depressive symptoms than those with ≥20 teeth (RR = 1.270, 95% CI: 1.040–1.560), reinforcing the relevance of severe structural loss when not mitigated by prosthetic rehabilitation. In Singapore, Tay et al. [115] reported that chewing disability was prospectively associated with a higher risk of clinically significant depressive symptoms over six years (RR = 1.480, 95% CI: 1.150–1.820), extending the adverse signal into the functional domain without, in the context of the present synthesis, establishing causality. Finally, in older Australian men, Wright et al. [118] found that limited chewing capacity was associated with depressive symptoms (PR = 1.930, 95% CI: 1.340–2.790). Across the stream, the reported adjusted ratio estimates ranged from 1.270 to 1.930, and every study pointed in the same adverse direction.

In summary, the RR/PR evidence provides convergent secondary support for the findings of the primary OR-only meta-analysis, although its inferential weight is more limited. The consistency of direction across all four studies strengthens the broader evidence base, yet the combination of mixed ratio metrics, mixed temporal frameworks, and limited study number limits the precision and generalizability of any cross-study summary conclusion. The most defensible interpretation is therefore that this secondary ratio-based stream corroborates, rather than independently anchors, the main adverse association between oral impairment and depressive outcomes in older adults.

### 3.11. Secondary Coefficient-Based Evidence: Structured Narrative Synthesis

Secondary continuous-effect evidence is summarized in Supplementary Materials Table S10. This stream comprised 10 studies (33.3% of the full review corpus) and was methodologically more heterogeneous than the ratio-based stream in both metric type and interpretation. Specifically, the retained studies reported five adjusted beta coefficients [92,93,109,114,121], three total effect beta coefficients [99,110,120], one standardized beta coefficient [112], and one two-stage least squares (2SLS) coefficient [101]. Seven studies were cross-sectional [92,93,99,101,109,110,120] and three were longitudinal [112,114,121]. Most were classified as high methodological quality, whereas two studies were rated as moderate-or-lower quality [93,109]. Because these coefficients differed materially in scale, estimand structure, and model assumptions, they were not pooled quantitatively and were instead retained for structured quantitative narrative synthesis.

Even within this heterogeneous continuous-effect stream, the overall directional pattern was broadly coherent. In studies where greater oral impairment was coded directly, worse oral status was generally associated with higher depressive symptom burden or greater depression severity [92,93,109,114,121]. Hajek et al. [92] reported that the presence of missing natural teeth was associated with higher Euro–D depressive symptom scores (adjusted beta = 0.210, 95% CI: 0.171–0.249), while Kaur et al. [93] found that increasing missing-teeth burden was associated with higher Patient Health Questionnaire-9 (PHQ–9) depression severity (adjusted beta = 0.122, 95% CI: 0.107–0.129). In Polish older adults, Skośkiewicz-Malinowska et al. [109] likewise showed that extracted-tooth burden was associated with greater depression severity (adjusted beta = 0.111, 95% CI: 0.105–0.116). Longitudinally, Tanaka et al. [114] reported that baseline oral frailty was prospectively associated with persistently worse depressive symptom trajectories (adjusted beta = 0.126, 95% CI: 0.062–0.189), and Zhang et al. [121] found that having only 1–9 natural teeth, compared with ≥20, was associated with higher depressive symptom burden at follow-up (adjusted beta = 0.590, 95% CI: 0.300–0.650). These findings support an adverse continuous-effect signal across both structural and functional oral exposures.

A complementary set of studies used better oral-health indicators as the exposure metric, and the coefficients were correspondingly negative, again supporting the same substantive direction. In China, Lou et al. [99] reported that denture use was associated with lower depressive symptom scores (total effect beta = −0.341, 95% CI: −0.505 to −0.178). Sun et al. [110] found that better self-rated oral health was associated with lower depressive symptom burden (total effect beta = −0.220, 95% CI: −0.440 to −0.010). In the Korean longitudinal panel study, Sung et al. [112] showed that better oral health was prospectively associated with fewer subsequent depressive symptoms, measured using the Geriatric Oral Health Assessment Index (GOHAI) as the oral-health indicator (standardized beta = −0.040, 95% CI: −0.060 to −0.030). In another Chinese population-based study, a greater number of remaining teeth was associated with lower depression levels (total effect beta = −0.018, 95% CI: −0.031 to−0.005) [120]. These inverse-coded results are fully compatible with the studies above once the substantive direction is harmonized, namely that better oral status or function corresponds to lower depressive burden, and conversely that worse oral status or function corresponds to greater depressive burden.

One study in this stream was more equivocal. In the U.S. instrumental-variable analysis by Matsuyama et al. [101], the retained older-adult subgroup estimate was a 2SLS coefficient of −0.055 (95% CI: −1.019 to 0.909), which was imprecise and compatible with no clear independent association. This estimate remains informative because it was derived from an instrumental-variable framework designed to strengthen causal inference compared with conventional observational regression models. Its inclusion reduces the risk of selectively overemphasizing statistically significant adverse findings and underscores that, even in a directionally coherent corpus, not every coefficient-based analysis yields a precise or independently compelling signal [101].

Overall, the coefficient-based stream supports the same broad substantive conclusion as the primary OR-only synthesis and the secondary RR/PR narrative stream: poorer oral structural or functional status tends to be associated with worse depressive outcomes in older adults. However, the inferential status of this stream is more limited, not because the direction of evidence is inconsistent, but because the coefficients themselves are not directly exchangeable across studies. The mixture of adjusted beta, total effect beta, standardized beta, and instrumental-variable estimates means that this evidence is best used to reinforce the cross-metric coherence of the oral–depression relationship rather than to generate a pooled continuous-effect summary of doubtful interpretability. In this sense, the coefficient-based narrative synthesis deepens the review by showing that the adverse oral–depression pattern is visible not only in binary odds-ratio models, but also across multiple continuous modeling frameworks with different statistical assumptions [92,93,99,101,109,110,112,114,120,121].

## 4. Discussion

### 4.1. Principal Findings and Integrated Interpretation

For clarity, the principal findings are interpreted below by considering the pooled association together with its heterogeneity and prediction interval, the exploratory subgroup and meta-regression results, the influence and small-study-effect analyses, and the complementary RR/PR and coefficient-based evidence. This systematic review and meta-analysis identified a broadly consistent adverse association between oral structural and functional impairment and depression-related outcomes in older adults. Across the qualitative evidence base, the direction of association was usually adverse, and the primary OR-only meta-analysis showed a statistically significant average-effect signal. Leave-one-out and influence diagnostics did not materially alter the direction of the pooled association, but the trim-and-fill sensitivity analysis materially attenuated the estimate from OR = 1.613 (95% CI: 1.311–1.986) to OR = 1.290 (95% CI: 0.990–1.690; *p* = 0.0584), so conventional statistical significance was not retained under this asymmetry-based scenario.

This result should not be interpreted either as a definitive null finding or as a corrected estimate of the true association. Trim-and-fill is an assumption-dependent sensitivity procedure based on funnel-plot asymmetry, and substantial between-study heterogeneity can itself contribute to asymmetry; accordingly, the imputed estimate cannot distinguish publication bias from other potential sources of small-study effects [87,88,90]. Its appropriate interpretation is that the pooled estimate and its conventional statistical significance are sensitive to assumptions about potentially missing small studies. Together with the substantial heterogeneity, wide prediction interval, and very low GRADE certainty, this attenuation materially reduces confidence in the robustness and generalizability of the pooled estimate.

The magnitude of this heterogeneity substantially limits the practical interpretability of the pooled OR: it should be regarded as an average association across heterogeneous study contexts rather than as a single effect magnitude expected to apply to an individual patient, population, or healthcare setting. Accordingly, the pooled estimate should not, in isolation, be used to define clinical thresholds, predict individual risk, or support policy-level effect estimates. The most defensible interpretation is that oral impairment is a relevant correlate or marker of late-life depressive vulnerability, not that a single pooled estimate can be generalized across all oral constructs and settings. Reverse causation remains a key concern, as depressive symptoms may also contribute to poorer oral health behaviors and outcomes, which cannot be fully disentangled in cross-sectional designs.

The secondary RR/PR and coefficient-based streams provide complementary rather than quantitatively interchangeable evidence. The RR/PR evidence is particularly informative because it includes temporally ordered and prevalence-based associations expressed on relative scales that differ from odds, thereby showing that the adverse oral–depression pattern is not confined to logistic-regression ORs. The coefficient-based stream adds a further dimension by showing that poorer oral status is also generally associated with greater depressive symptom burden when depression is modeled continuously, while studies coding better oral health tend to show coefficients in the opposite direction. Together, these streams strengthen the cross-metric directional coherence of the evidence and indicate that the observed association is not an artifact of a single outcome scale or statistical model.

However, their contribution is primarily qualitative and triangulatory rather than confirmatory in a pooled quantitative sense: RR and PR estimates differ in estimand and temporal interpretation, while adjusted, standardized, total-effect, and instrumental-variable coefficients differ in scale, model structure, and covariate adjustment. Their magnitudes therefore should not be compared directly with one another or with the pooled OR, and the relatively small number of studies in each secondary stream limits precision and the strength of independent inference.

The overall interpretation is cautious but clinically meaningful. The consistency of direction across structural, functional, prosthetic, salivary, and oral-impact domains, together with the longitudinal findings and the consistent direction of results across different effect measures, supports the relevance of oral impairment within the broader set of clinical and psychosocial vulnerabilities associated with late-life depression. However, the current evidence base remains observational, heterogeneous, and potentially affected by residual confounding and small-study distortion; accordingly, causal claims should be avoided.

### 4.2. Sources of Heterogeneity and Effect Modification

The high between-study heterogeneity (I^2^ = 88.1%) is most plausibly understood as reflecting a combination of clinical and methodological diversity rather than a single source of variation. Accordingly, the overall OR is retained as a descriptive cross-domain average rather than as evidence of a single homogeneous oral–depression effect; when interpreting the magnitude associated with a specific oral construct, the corresponding construct-specific subgroup estimate and its within-domain heterogeneity are more informative than the overall pooled estimate. Structural oral impairment, particularly tooth loss, severe dentition loss, and edentulism, showed a largely adverse pattern across both cross-sectional and longitudinal studies. Functional oral impairment also showed a coherent adverse signal, with oral frailty, chewing difficulty, chewing disability, and broader oral-function decline repeatedly associated with worse depressive outcomes. The subgroup analyses are compatible with this interpretation, but they should not be overread. Tooth loss/dentition yielded the most numerically stable pooled subgroup estimate, whereas oral frailty/functional decline yielded the largest pooled effect. Because several subgroup strata were sparse, the safest conclusion is not that one oral-exposure family is definitively stronger than another, but that exposure definition materially influences effect size, precision, and interpretability.

Study design also matters for interpretation. Cross-sectional studies tended to show stronger pooled associations than longitudinal studies, whereas the prospective evidence showed associations in the same adverse direction, although of smaller magnitude. This pattern is compatible with a mixture of contemporaneous symptom clustering, shared vulnerability, and partial temporal ordering, and it argues against causal simplification in either direction. The meta-regression findings further suggest that heterogeneity was structured more by oral-construct definition and ascertainment than by region or broad quality grouping. This is clinically plausible, because tooth counts, oral frailty indices, chewing-related measures, oral impacts, and broader self-rated oral-health indicators are related but not interchangeable constructs. Still, these findings remain exploratory and should be treated as hypothesis-generating only. Importantly, the residual heterogeneity cannot be attributed confidently to a single source or interpreted as evidence of true effect modification. Part of the variation may represent genuine differences in association across populations, oral-impairment domains, and clinical or regional contexts; however, methodological differences in exposure ascertainment, depression measurement, study design, covariate adjustment, residual confounding, and sampling may generate or amplify the same pattern. The subgroup and meta-regression analyses therefore cannot reliably distinguish biological or clinical effect modification from methodological artifact, particularly given the modest number of studies and sparse moderator strata. The absence of statistically significant moderation by region or broad study-quality grouping should likewise not be interpreted as evidence that these characteristics are irrelevant, because the available study-level analyses had limited power to detect such differences.

Overall, the interpretation by oral-exposure domain and study design strengthens, rather than weakens, the main findings of the review. The adverse association was not confined to one exposure family or one epidemiological design, but its magnitude and interpretability varied systematically across oral constructs and analytical contexts. The most defensible conclusion is therefore that oral structural and functional impairment is best understood as a family of related later-life vulnerability markers, some of which may function more as stable background indicators and others as more immediate functional correlates of depressive burden. This distinction is important for both research design and clinical translation, because it suggests that the oral–depression relationship in older adults should be studied and addressed in a construct-specific rather than reductionist way.

### 4.3. Biological Plausibility and Clinical Implications

The findings are biologically plausible and clinically relevant. They support a multidimensional model in which oral impairment may be linked to depressive outcomes through interacting inflammatory, functional, nutritional, and psychosocial pathways. Oral inflammation, dysbiosis, impaired mastication, reduced dietary quality, frailty-related decline, oral discomfort, social embarrassment, and lower oral health-related quality of life may all contribute to a broader late-life vulnerability profile. These pathways should be regarded as biologically and clinically plausible explanatory mechanisms rather than as causal pathways demonstrated by the evidence synthesized in this review. Given the observational nature of the included studies, the present findings cannot establish whether oral impairment directly influences depressive outcomes, whether depressive symptoms adversely affect oral health and function, or whether both are partly explained by shared underlying determinants. From a clinical perspective, these findings support comprehensive oral assessment rather than an approach based primarily on tooth counts or acute dental pathology. In older adults, oral evaluation should extend beyond tooth counts and acute pathology to include chewing function, oral frailty indicators, oral dryness, prosthetic adequacy, and the functional consequences of oral impairment. This is especially relevant in geriatric and gerontopsychiatric settings, where oral difficulties may coexist with frailty, poor nutrition, reduced participation, and social withdrawal.

The reverse clinical implication is also relevant at the level of assessment, though not of causal direction: depressive symptoms in later life should prompt broader attention to oral status and oral function. The practical implication is not that oral rehabilitation alone can be assumed to prevent or treat depression, but that oral impairment may identify an area for integrated assessment and potentially modifiable support within multidisciplinary care. These findings suggest that oral health assessment may represent a relevant but currently underutilized component of geriatric psychiatric evaluation. Screening for oral impairment could help identify individuals with a greater burden of factors associated with depressive symptoms and support more integrated multidisciplinary care approaches, without implying that oral impairment independently predicts or causes subsequent depression. These findings support consideration of oral status and oral function within comprehensive geriatric mental health assessment, but they do not establish that oral impairment is an independent causal determinant of depression or that oral rehabilitation alone will reduce depressive outcomes.

### 4.4. Comparison with Previous Reviews, Specific Added Value, and Implications for Future Research

Compared with earlier reviews that synthesized broad oral-health constructs or broader aging outcomes, the present review focused specifically on oral-exposure-to-depression associations in older adults and preserved effect-metric comparability by separating OR, RR/PR, and coefficient-based evidence streams [163,164,165,166]. This approach improves interpretability for geriatric psychiatry because it keeps the primary pooled model closer to the meaning of the original analytical estimates.

At the same time, this more focused design does not remove the fundamental limitations of the underlying evidence base. The available studies remain observational, heterogeneous in exposure and outcome definitions, and vulnerable to residual confounding. The main added value of the present review is therefore not a claim of definitive causal clarity, but a more disciplined and clinically interpretable synthesis of the available older-adult evidence.

Future research should address several limitations of the current evidence base. First, greater emphasis should be placed on adequately powered longitudinal studies with repeated assessments of both oral status and depressive outcomes. Such studies should establish temporal ordering more clearly, incorporate sufficiently long follow-up, account for baseline oral status and depressive symptoms, and rigorously address major shared determinants, including frailty, multimorbidity, socioeconomic disadvantage, nutritional status, cognitive impairment, medication burden, healthcare access, and health-related behaviors. These design features would improve assessment of temporally ordered associations while reducing, although not eliminating, concerns regarding residual confounding and reverse causation.

Second, greater standardization of oral-impairment definitions and measurement is needed. The present evidence base encompasses clinically distinct constructs, including tooth loss and dentition status, chewing dysfunction, oral frailty, xerostomia, prosthetic status, and oral health-related quality-of-life impairment. These domains should not be treated as interchangeable indicators of a single oral-health construct. Future studies should use clearly defined exposure domains, validated and reproducible assessment protocols, consistently reported severity thresholds, and, where appropriate, complementary objective and patient-reported measures. Depression outcomes should likewise be assessed using validated instruments with clearly reported thresholds or diagnostic criteria and with explicit distinction between depressive symptoms, symptom severity, and clinically diagnosed depression.

Third, prospective research should examine whether the observed associations vary according to clinically relevant characteristics and whether nutritional vulnerability, functional decline, pain, loneliness, social participation, or other psychosocial factors may explain or modify the observed patterns. Where sufficiently powered, studies should evaluate differences according to age, sex, frailty, cognitive status, socioeconomic position, residential setting, and clinically distinct oral-impairment domains. Such analyses should be interpreted as investigations of potential explanatory pathways or effect heterogeneity rather than as demonstrations of causality unless supported by appropriate study designs.

Finally, oral health should be more explicitly incorporated into geriatric mental-health and gerontopsychiatric research frameworks. This does not imply that oral impairment is currently an established independent predictor of depression or that oral treatment has been demonstrated to prevent depressive illness. Rather, multidisciplinary prospective research should determine whether structured oral-health assessment adds clinically useful information to comprehensive geriatric and gerontopsychiatric assessment and whether coordinated dental, geriatric, nutritional, primary-care, and mental-health approaches improve patient-centered outcomes. Ultimately, appropriately designed prospective and interventional studies will be required before the observational associations identified in this review can support preventive or therapeutic recommendations.

### 4.5. Strengths and Methodological Limitations of the Review

This review has several strengths. It addressed a clearly defined exposure-outcome question in which oral structural and functional impairment was consistently treated as the exposure and depression-related outcomes as the endpoints; it used dual-reviewer screening and adjudication procedures; and it relied on a frozen master dataset with reconciled analytic streams. A further strength was the deliberately structured quantitative strategy, which kept the principal OR-only meta-analysis separate from secondary RR/PR and coefficient-based evidence when formal pooling would have obscured metric meaning. Another strength is the breadth of robustness assessment. Rather than relying on a single pooled estimate, the review incorporated exploratory subgroup analyses, univariate meta-regression, leave-one-out diagnostics, influence assessment, Baujat analysis, funnel-plot asymmetry testing, and trim-and-fill sensitivity analysis. This allowed the primary pooled estimate to be interpreted alongside evidence of heterogeneity, influence, and possible small-study effects rather than in isolation.

Several limitations should also be emphasized. First, the evidence base was entirely observational and therefore remains vulnerable to residual confounding, imperfect temporality, and bidirectional reinforcement. Consequently, neither the cross-sectional associations nor the temporally ordered associations observed in longitudinal studies should be interpreted as demonstrating that oral impairment causes subsequent depression. Reverse causation and shared determinants—including frailty, multimorbidity, socioeconomic disadvantage, nutritional vulnerability, and health-related behaviors—remain plausible alternative or complementary explanations for the observed associations.

Second, heterogeneity in the primary OR-only meta-analysis was substantial, several subgroup analyses were based on few studies, and the asymmetry analyses indicated that the pooled estimate was sensitive to potential small-study effects. The high residual heterogeneity limits the generalizability of the pooled estimate and precludes interpreting the summary OR as a uniform association across populations or clinical settings. The high residual heterogeneity limits the generalizability of the pooled estimate and precludes interpreting the summary OR as a uniform association across populations or clinical settings. Although subgroup analyses and meta-regression identified exploratory variation according to oral-impairment construct and ascertainment, the available study-level data were insufficient to determine whether the remaining heterogeneity reflected true effect modification, methodological variation, residual confounding, or a combination of these factors. Third, exposure and outcome definitions were clinically meaningful but heterogeneous, encompassing structurally different oral constructs and multiple depressive instruments. Taken together, these limitations explain the very low GRADE certainty assigned to the primary synthesized evidence: the observational design and residual confounding restricted the initial level of confidence, while serious inconsistency and sensitivity to possible small-study effects further weakened confidence in the precision and generalizability of the pooled estimate. The generally moderate-to-high NOS scores of individual studies do not offset these body-level concerns, because study-level methodological quality and certainty in a pooled evidence body address different questions.

Geographic representation also limits external validity. Twenty-one of the 30 included studies (70.0%) were conducted in Asian settings, and 12 of the 15 studies contributing to the primary OR-only meta-analysis were from Asia. Although the pooled ORs were numerically similar in Asian and non-Asian subgroup analyses, the non-Asian subgroup contained only three studies and therefore provided limited power for meaningful geographic comparison, while heterogeneity within the Asian subgroup remained very high. Differences in oral-health infrastructure, access to dental and mental-health services, cultural expectations regarding dentition and prosthetic care, population characteristics, and healthcare systems may influence both exposure ascertainment and the clinical meaning of oral impairment across regions. Accordingly, the pooled estimate should not be assumed to be directly generalizable to European, North American, or other underrepresented populations, and the absence of statistically significant regional moderation should not be interpreted as evidence of geographic equivalence.

The secondary RR/PR and coefficient-based evidence also have important interpretative limitations: they include relatively few studies and comprise non-exchangeable estimands, scales, and modeling approaches. Consequently, their numerical magnitudes cannot be directly compared with one another or with the pooled OR, and their principal value is as complementary evidence of directional consistency rather than as precise independent summary effects.

The NOS itself also has limitations as a study-level appraisal instrument. Its summary star score can mask domain-specific bias patterns and does not capture confounding, selection, measurement, and missing-data bias with the same granularity as newer domain-based risk-of-bias frameworks. Although neither categorical NOS quality nor the continuous NOS total score showed a statistically detectable relationship with effect magnitude in exploratory meta-regression, these null findings should be interpreted cautiously because the primary OR-only meta-analysis included only 15 studies and NOS scores were concentrated within a relatively narrow range.

Fourth, the review intentionally focused on studies published from 2016 onward, which improved contemporary relevance but may have excluded older eligible studies. This publication-date restriction may therefore have excluded relevant earlier studies, including potentially influential or foundational evidence, and may have introduced time-window selection bias. Accordingly, the findings should be interpreted as a synthesis of the contemporary evidence published from 2016 onward rather than as an exhaustive synthesis of the entire historical evidence base concerning the association between oral impairment and depressive outcomes in older adults. Because the publication-date restriction was applied at the database-retrieval stage, records published before 2016 were not systematically captured or screened, and the present review dataset therefore cannot provide a reliable estimate of how many additional studies would otherwise have met the eligibility criteria. A valid sensitivity analysis using a broader publication window would require a de novo rerun of the complete six-database search, deduplication, screening, eligibility adjudication, extraction, and synthesis workflow under the expanded timeframe rather than a simple reanalysis of the frozen dataset; this post hoc scope expansion was not undertaken in the present revision. Future updates of the review should consider searching from database inception to determine whether inclusion of earlier longitudinal evidence materially changes the magnitude, heterogeneity, or interpretation of the synthesized association.

Review-process limitations should also be recognized. The review was not prospectively registered, and although the eligibility framework, effect-selection rules, and analytic streams were finalized before quantitative synthesis, public preregistration would have provided an additional safeguard against post hoc analytical drift. Accordingly, despite the use of frozen analytical datasets and prespecified effect-selection rules, selective analytical decision-making cannot be excluded with the same degree of confidence that would have been afforded by a prospectively registered protocol. The present findings should therefore be interpreted as informative for integrated geriatric and gerontopsychiatric assessment, but not as a basis for strong causal inference.

### 4.6. Clinical Practice and Policy Implications

From a clinical perspective, the findings support closer integration of oral assessment into geriatric and gerontopsychiatric care, particularly for older adults with frailty, poor nutrition, social withdrawal, or depressive symptoms. Oral assessment in later life should extend beyond counting teeth or documenting acute pathology and should also consider chewing function, oral dryness, prosthetic adequacy, oral frailty indicators, and the everyday functional consequences of oral impairment.

At the level of practice and policy, the key implication is that oral impairment should not be regarded as an isolated dental issue in later life. It may represent a clinically relevant marker of mental-health vulnerability that warrants integration into multidisciplinary assessment. Even so, the current evidence does not justify assuming that oral rehabilitation alone will prevent or treat depression.

These clinical implications should nevertheless be interpreted in light of the composition of the evidence base. Eighteen of the 30 included studies were cross-sectional, and most participants were drawn from community-based, population-based, or nationally representative samples rather than from institutional, specialist geriatric, or psychiatric care settings. The available evidence therefore supports recognition of oral impairment as a clinically relevant correlate within multidimensional geriatric assessment, but it provides more limited support for prognostic use, referral thresholds, or intervention decisions in specific clinical populations. The predominance of Asian studies further restricts direct extrapolation to underrepresented healthcare systems and populations. Accordingly, the practice implications described here should be regarded as principles for multidisciplinary awareness and assessment rather than as evidence-based clinical rules applicable uniformly across care settings.

Operationally, these findings support role-specific coordination within older-adult care rather than a new stand-alone screening mandate. Dental professionals can identify oral structural and functional problems—including chewing difficulty, xerostomia, prosthetic inadequacy, oral pain, and oral frailty indicators—and should be attentive to coexisting depressive symptoms, social withdrawal, or functional decline that may warrant communication with the patient’s physician or mental-health team. Physicians and geriatric clinicians can incorporate oral status and oral function into broader assessment of frailty, nutrition, multimorbidity, medication burden, and depressive symptoms and facilitate dental evaluation when clinically relevant oral problems are identified. Mental-health professionals can consider oral pain, impaired eating, appearance-related concerns, reduced social participation, and oral self-care difficulties as potentially relevant components of the patient’s overall symptom and functional burden and can support referral for dental assessment when indicated. Such interprofessional coordination is consistent with integrated, patient-centered care for older adults, but the present observational evidence does not establish that dental treatment will prevent or improve depression, nor does it define a validated oral-health threshold for psychiatric referral [5,13].

## 5. Conclusions

In this systematic review and meta-analysis of observational studies, poorer oral structural and functional status was associated, on average, with worse depressive outcomes in older adults. However, the current evidence base remains observational, heterogeneous, and of very low certainty, and possible small-study effects further limit confidence in the pooled estimate. These findings should therefore be interpreted as supportive of integrated geriatric and gerontopsychiatric assessment rather than as evidence for causal inference or stand-alone treatment recommendations. Future research should prioritize adequately powered longitudinal studies with repeated oral and depressive assessments, standardized and clinically distinct definitions of oral impairment, rigorous control of shared geriatric and socioeconomic determinants, and evaluation of whether structured oral-health assessment can be meaningfully integrated into multidisciplinary geriatric mental-health frameworks. Such evidence is required before the present observational associations can support preventive, prognostic, or treatment recommendations.

## Figures and Tables

**Table 1:** Primary OR-only random-effects synthesis of oral impairment and depressive outcomes in older adults: overall cross-domain estimate and exposure-family-stratified results.

Panel A. Overall cross-domain OR-only synthesis.			
**Domain**	**Statistic**		**Value**
Pooled effect	Number of studies (k)		15
Pooled effect	Odds Ratio (OR, 95% CI)		1.613 (1.311–1.986)
Pooled effect	Test statistic for overall effect		t(14) = 4.94, df = 14
Pooled effect	*p*-value (overall effect)		0.0002
Heterogeneity	Cochran’s Q test (χ^2^)		117.40
Heterogeneity	*p*-value (Q test)		<0.0001
Heterogeneity	I^2^ (95% CI), %		88.1% (82.0–92.1%)
Heterogeneity	τ^2^ (95% CI)		0.0817 (0.0360–0.4410)
Heterogeneity	τ (95% CI)		0.2860 (0.1910–0.6640)
Heterogeneity	Higgins’ H (95% CI)		2.896 (2.357–3.557)
Prediction interval (PI)	95% Prediction Interval (OR)		0.849–3.065
Publication bias/small-study effects	Egger’s intercept (95% CI)		2.13 (0.31–3.95)
Publication bias/ Small-study effects	t-statistic		2.299
Publication bias/ Small-study effects	*p*-value (Egger’s test)		0.039
Panel B. Exposure-family-stratified primary presentation.			
**Exposure family**	**k**	**Pooled OR (95% CI)**	**I^2^ (%)**
Tooth loss/dentition	5	1.326 (1.247–1.409)	0.0
Oral frailty/functional decline	3	2.967 (1.459–6.037)	33.8
Chewing dysfunction	2	1.419 (0.550–3.659)	47.6
OHRQoL/oral impacts	2	2.529 (0.001–7225.535)	66.3
Other oral impairment	2	1.349 (0.064–28.347)	77.6
Xerostomia/salivary dysfunction	1	2.163 (1.200–3.900); single study, not pooled	Not estimable

**Notes:** The primary quantitative synthesis was based on a random-effects inverse-variance meta-analysis restricted to OR-based estimates included in the primary meta-analysis. Pooled odds ratios (ORs) and 95% confidence intervals (CIs) were estimated on the logarithmic scale and back-transformed for presentation. Between-study variance was estimated using restricted maximum likelihood (REML), and inference for the overall pooled effect was based on the Hartung–Knapp adjustment; accordingly, the overall effect was tested using a t statistic with 14 degrees of freedom. Heterogeneity was summarized using Cochran’s Q, I^2^, τ^2^, τ, and Higgins’ H, each with corresponding 95% CIs where available. The 95% prediction interval was calculated to reflect the range of effects plausibly expected in a new comparable study. Egger’s regression test is reported as an indicator of funnel-plot asymmetry and possible small-study effects rather than as definitive proof of publication bias. All estimates are reported exactly as generated in the frozen analytical workflow. Panel B presents the exposure-family-stratified estimates already generated within the frozen analytical workflow and reported in Supplementary Table S12. These estimates are provided to avoid implying clinical exchangeability among distinct oral-impairment constructs. Subgroups containing only two or three studies should be interpreted cautiously because Hartung–Knapp confidence intervals may be very wide when the number of studies is small. Xerostomia/salivary dysfunction was represented by one eligible OR study and was therefore not meta-analytically pooled as an independent exposure family.

**Table 2:** Trim-and-fill-adjusted random-effects sensitivity analysis of the primary OR-only meta-analysis of oral structural and functional impairment and depressive outcomes in older adults: adjusted pooled effect, heterogeneity, and prediction interval.

Domain	Statistic	Value
Trim-and-Fill (sensitivity)	Number of imputed studies	7
	Adjusted number of studies (*k*)	22
Adjusted pooled effect	Adjusted Odds Ratio (OR, 95% CI)	1.290 (0.990–1.690)
	Test statistic for overall effect	t(21) = 2.000, df = 21
	*p*-value (overall effect)	0.0584
Adjusted heterogeneity	Cochran’s Q test (χ^2^)	193.23
	*p*-value (Q test)	<0.0001
	I^2^ (95% CI), %	89.1% (85.5–91.6%)
	τ^2^ (95% CI)	0.2245 (0.1370–0.8570)
	τ (95% CI)	0.4740 (0.3700–0.9260)
	Higgins’ H statistic (95% CI)	3.030 (2.630–3.460)
Adjusted prediction interval (PI)	95% Prediction Interval (OR)	0.470–3.560

**Notes**: The trim-and-fill sensitivity analysis was performed on the augmented OR-only dataset after imputation of 7 potentially missing studies. The adjusted pooled odds ratio (OR) and its 95% confidence interval (CI) were derived from the trim-and-fill random-effects model generated within the frozen analytical workflow. Between-study variance remained estimated using restricted maximum likelihood (REML), and inference for the adjusted pooled effect was based on the corresponding t statistic with 21 degrees of freedom. Heterogeneity confidence intervals were calculated from the augmented study-level log odds ratios and corresponding standard errors. The 95% CI for τ^2^ was obtained using the Q-profile method, the 95% CI for τ was derived by square-root transformation of the τ^2^ limits, and the 95% CIs for I^2^ and Higgins’ H were estimated using non-central chi-square-based heterogeneity interval methods. The 95% prediction interval reflects the range of effects plausibly expected in a new comparable study after trim-and-fill augmentation. *p* values below 0.0001 are reported as <0.0001.

## Data Availability

No individual participant data were generated. This study used data extracted from published articles. The derived study-level extraction sheet, effect-size harmonization file, and analytic tables supporting the findings are provided in the Supplementary Materials.

## Supplementary Materials

The following supporting information can be downloaded at: https://www.mdpi.com/article/10.3390/diseases14090309/s1, Table S1. Database-specific search strategies and query structures used in the systematic review, Table S2. Overview of study-level contribution to the qualitative and/or quantitative evidence synthesis, Table S3. Studies excluded from the qualitative and/or quantitative evidence synthesis, with reasons for exclusion based on prespecified eligibility criteria, Table S4. Included studies and their principal methodological, population, exposure, outcome, and selected effect-estimate characteristics, Table S5. Study-level Newcastle–Ottawa Scale (NOS) assessments of included studies (Panel A) and body-level GRADE certainty judgments for the synthesized evidence streams (Panel B); Panel A. Study-level Newcastle–Ottawa Scale (NOS) assessment of included studies; Panel B. Body-level GRADE certainty judgments for the synthesized evidence streams, Table S6. Frozen master effect-selection dataset for the 30 studies retained in the final review corpus, including analytic-stream assignment, selected effect estimates, stream-eligibility judgments, and planned downstream use, Table S7. Reconciliation of derived analytic streams with the frozen master effect-selection dataset (Table S6), including quantitative-stream coverage and qualitative-only retention, Table S8. Primary Odds ratio (OR)-only backbone dataset used in the final quantitative workflow, Table S9. Secondary RR/PR dataset retained for structured quantitative narrative synthesis, Table S10. Secondary beta/coefficient dataset retained for structured quantitative narrative synthesis, Table S11. Leave-one-out diagnostics for the primary OR-only random-effects meta-analysis, Table S12. Exploratory subgroup random-effects analyses within the primary Odds ratio (OR)-only backbone, Table S13. Exploratory univariate mixed-effects meta-regression summary for the primary Odds ratio (OR)-only backbone, Table S14. Coefficient-level results from exploratory univariate mixed-effects meta-regression models for the primary Odds ratio (OR)-only backbone, Table S15. Study-level influence diagnostics for the primary Odds ratio (OR)-only random-effects model, Table S16. Study-level Baujat diagnostic data for the primary Odds ratio (OR)-only backbone.
