# Supplementary material for: Oral Structural and Functional Impairment in Relation to Depressive Outcomes in Older Adults: A Systematic Review and Meta-Analysis of Observational Studies
Source: Diseases. 2026 Aug 26;14(9):309. doi: 10.3390/diseases14090309 (PMC13605695; doi:10.3390/diseases14090309)
Supplement: Supplementary file 1 [file diseases-14-00309-s001.zip › diseases-4470095_Supplementary_Materials.pdf]

Review

Supplementary Materials for:

"Oral Structural and Functional Impairment in Relation to Depressive Outcomes in Older Adults: A Systematic Review and Meta–analysis of Observational Studies"

Table S1. Database-specific search strategies and query structures used in the systematic review.

Search ID	Access Platform	Source Database	Search Execution Date(s)	Resource Types Searched	Full Search Strategy and Search Terms
S1–PUB	PubMed (NCBI)	MEDLINE/ PubMed	5 January 2026; updated rerun 19 April 2026	Journal articles; ahead–of–print records; indexed and publisher–supplied records	Date limits: 1 January 2016 to 5 January 2026. Language restrictions: none. Search string: ((aged[MeSH Terms] OR "Aged, 80 and over"[MeSH Terms] OR elderly[tiab] OR elder*[tiab] OR "older adult*"[tiab] OR "older people"[tiab] OR senior*[tiab] OR geriatric*[tiab] OR ageing[tiab] OR aging[tiab]) AND (("Tooth Loss"[MeSH Terms] OR "Mastication"[MeSH Terms] OR "Mouth Diseases"[MeSH Terms] OR "Oral Health"[MeSH Terms] OR edentulism[tiab] OR edentulous[tiab] OR "tooth loss"[tiab] OR "missing teeth"[tiab] OR "number of teeth"[tiab] OR dentition[tiab] OR "severe tooth loss"[tiab] OR "remaining teeth"[tiab] OR "functional dentition"[tiab] OR "chewing difficulty"[tiab] OR "chewing difficulties"[tiab] OR masticat*[tiab] OR "oral function"[tiab] OR "oral function decline"[tiab] OR "oral hypofunction"[tiab] OR "oral frailty"[tiab] OR xerostomia[tiab] OR "dry mouth"[tiab] OR "mouth pain"[tiab] OR "oral pain"[tiab] OR denture*[tiab] OR

					prosthes*[tiab] OR "oral health–related quality of life"[tiab] OR OHRQoL[tiab] OR OIDP[tiab] OR GOHAI[tiab])) AND (("Depression"[MeSH Terms] OR "Depressive Disorder"[MeSH Terms] OR depress*[tiab] OR "depressive symptom*"[tiab] OR "depressive disorder"[tiab] OR "depression symptoms"[tiab] OR "late–life depression"[tiab] OR "incident depression"[tiab])) NOT (animals[MeSH Terms] NOT humans[MeSH Terms]))
S1–SCO	Scopus (Elsevier)	Scopus	5 January 2026; updated rerun 19 April 2026	Articles; articles in press; conference records retrieved if matched and screened for eligibility	Date limits: 1 January 2016 to 5 January 2026. Language restrictions: none. Search string: TITLE–ABS–KEY ((elderly OR elder* OR "older adult*" OR "older people" OR senior* OR geriatric* OR ageing OR aging) AND ("tooth loss" OR "missing teeth" OR "number of teeth" OR dentition OR edentulism OR edentulous OR "severe tooth loss" OR "remaining teeth" OR "functional dentition" OR "chewing difficulty" OR "chewing difficulties" OR masticat* OR "oral function" OR "oral function decline" OR "oral hypofunction" OR "oral frailty" OR xerostomia OR "dry mouth" OR "mouth pain" OR "oral pain" OR denture* OR prosthes* OR "oral health" OR "oral health–related quality of life" OR OHRQoL OR OIDP OR GOHAI) AND (depress* OR "depressive symptom*" OR "depressive disorder" OR "late–life depression" OR "incident depression")) AND PUBYEAR > 2015
S1–WOS	Web of Science (Clarivate)	Web of Science Core Collection	5 January 2026; updated rerun 19 April 2026	Articles; Early Access records; proceedings records screened if retrieved	Date limits: 1 January 2016 to 5 January 2026. Language restrictions: none. Search string: TS=((elderly OR elder* OR "older adult*" OR "older people" OR senior* OR geriatric* OR ageing OR aging) AND ("tooth loss" OR "missing teeth" OR "number of teeth" OR dentition OR edentulism OR edentulous OR "severe tooth loss" OR "remaining teeth" OR "functional dentition" OR "chewing difficulty" OR "chewing difficulties" OR masticat* OR

					"oral function" OR "oral function decline" OR "oral hypofunction" OR "oral frailty" OR xerostomia OR "dry mouth" OR "mouth pain" OR "oral pain" OR denture* OR prosthes* OR "oral health" OR "oral health–related quality of life" OR OHRQoL OR OIDP OR GOHAI) AND (depress* OR "depressive symptom*" OR "depressive disorder" OR "late–life depression" OR "incident depression"))
S1–EMB	Ovid	Embase	5 January 2026; updated rerun 19 April 2026	Embase–indexed journal records; conference abstracts retrievable but excluded later if insufficient for eligibility or extraction	Date limits: 1 January 2016 to 5 January 2026. Language restrictions: none. Search string: 1. (elderly or elder* or older adult* or older people or senior* or geriatric* or ageing or aging).ti,ab,kf. 2. exp aged/ or exp very elderly/ 3. 1 or 2 4. exp tooth loss/ or exp edentulousness/ or exp mastication/ or exp oral health/ or exp xerostomia/ or exp denture/ 5. ("tooth loss" or "missing teeth" or "number of teeth" or dentition or edentulism or edentulous or "severe tooth loss" or "remaining teeth" or "functional dentition" or "chewing difficulty" or "chewing difficulties" or masticat* or "oral function" or "oral function decline" or "oral hypofunction" or "oral frailty" or xerostomia or "dry mouth" or "mouth pain" or "oral pain" or denture* or prosthes* or "oral health" or "oral health–related quality of life" or OHRQoL or OIDP or GOHAI).ti,ab,kf. 6. 4 or 5 7. exp depression/ or exp depressive disorder/ 8. (depress* or "depressive symptom*" or "depressive disorder" or "late–life depression" or "incident depression").ti,ab,kf. 9. 7 or 8 10. 3 and 6 and 9 11. limit 10 to yr="2016–Current"
S1–PSY	Ovid	APA PsycInfo	5 January 2026; updated rerun 19 April 2026	Journal articles and indexed psychological literature	Date limits: 1 January 2016 to 5 January 2026. Language restrictions: none. Search string: 1. (elderly or elder* or older adult* or older people or senior* or geriatric* or ageing or aging).ti,ab,kf. 2. exp aging/ or exp gerontology/ 3. 1 or 2 4. ("tooth loss" or "missing teeth" or

					"number of teeth" or dentition or edentulism or edentulous or "severe tooth loss" or "remaining teeth" or "functional dentition" or "chewing difficulty" or "chewing difficulties" or masticat* or "oral function" or "oral function decline" or "oral hypofunction" or "oral frailty" or xerostomia or "dry mouth" or "mouth pain" or "oral pain" or denture* or prosthes* or "oral health" or "oral health–related quality of life" or OHRQoL or OIDP or GOHAI).ti,ab,kf. 5. exp oral health/ or exp oral hygiene/ 6. 4 or 5 7. exp depression/ or exp major depression/ 8. (depress* or "depressive symptom*" or "depressive disorder" or "late–life depression" or "incident depression").ti,ab,kf. 9. 7 or 8 10. 3 and 6 and 9 11. limit 10 to yr="2016–Current"
S1–CIN	EBSCOhost	CINAHL Complete/ CINAHL Ultimate	5 January 2026; updated rerun 19 April 2026	Nursing and allied health journal records	Date limits: 1 January 2016 to 5 January 2026. Language restrictions: none. Search string: ((MH "Aged+") OR TI (elderly OR elder* OR "older adult*" OR "older people" OR senior* OR geriatric* OR ageing OR aging) OR AB (elderly OR elder* OR "older adult*" OR "older people" OR senior* OR geriatric* OR ageing OR aging)) AND (((MH "Tooth Loss+") OR (MH "Edentulism+") OR (MH "Oral Health+") OR (MH "Mastication+") OR (MH "Xerostomia+") OR TI ("tooth loss" OR "missing teeth" OR "number of teeth" OR dentition OR edentulism OR edentulous OR "severe tooth loss" OR "remaining teeth" OR "functional dentition" OR "chewing difficulty" OR "chewing difficulties" OR masticat* OR "oral function" OR "oral function decline" OR "oral hypofunction" OR "oral frailty" OR xerostomia OR "dry mouth" OR "mouth pain" OR "oral pain" OR denture* OR prosthes* OR "oral health" OR "oral health–related quality of life" OR OHRQoL OR OIDP OR GOHAI) OR AB ("tooth loss" OR "missing teeth" OR "number of teeth" OR dentition OR

	edentulism OR edentulous OR "severe tooth loss" OR "remaining teeth" OR "functional dentition" OR "chewing difficulty" OR "chewing difficulties" OR masticat* OR "oral function" OR "oral function decline" OR "oral hypofunction" OR "oral frailty" OR xerostomia OR "dry mouth" OR "mouth pain" OR "oral pain" OR denture* OR prosthes* OR "oral health" OR "oral health–related quality of life" OR OHRQoL OR OIDP OR GOHAI))) AND (((MH "Depression+") OR TI (depress* OR "depressive symptom*" OR "depressive disorder" OR "late–life depression" OR "incident depression") OR AB (depress* OR "depressive symptom*" OR "depressive disorder" OR "late–life depression" OR "incident depression")))

Notes: Searches were designed to maximize sensitivity while maintaining conceptual specificity for studies evaluating oral structural and functional impairment in relation to depressive outcomes in older adults. No language restrictions were applied. All original database searches were executed on 5 January 2026. An updated search using the same strategies and eligibility criteria was rerun across all databases on 19 April 2026, and any newly eligible studies were incorporated into the final review corpus before submission. The 2016 restriction was applied to prioritize contemporary evidence reflecting recent conceptual developments in oral frailty and geriatric functional assessment; however, this approach may have excluded earlier foundational studies and should be interpreted as a trade–off between relevance and historical completeness. Records retrieved from the six databases were managed in Mendeley Reference Manager (version 2.143) before deduplication and screening. Because restrictive study–design filters may reduce retrieval sensitivity, observational–study eligibility was determined during title/abstract screening and full–text review rather than by applying narrow design filters at the search stage. Records were considered eligible only when oral structural or functional impairment was treated as the exposure and depressive outcomes were treated as the endpoint; studies with reversed analytic directionality were excluded during screening. Search strategies were developed iteratively by the review team and adapted to each database platform to preserve conceptual equivalence, transparency, and reproducibility.

Table S2. Overview of study-level contribution to the qualitative and/or quantitative evidence synthesis.

Record ID	Authors (Year)	Study Design	Included in Qualitative Synthesis	Included in Quantitative Synthesis	Key Contribution to the Review
IN–001	Hajek et al. (2023) [92]	Cross–sectional, population–based, multicountry observational study (Survey of Health, Ageing and Retirement in Europe (SHARE) wave 5)	Yes	Yes	Large multicountry ageing sample showing that poorer oral status was associated with higher depressive symptom burden; contributed to the beta/coefficient evidence stream.
IN–002	Kaur et al. (2024) [93]	Cross–sectional institution–based observational study	Yes	Yes	Institutionalized older–adult sample supporting an association between poorer oral status and greater depression severity; contributed one interpretable adjusted beta estimate.
IN–003	Kawamura et al. (2026) [94]	Cross–sectional outpatient observational study	Yes	Yes	Geriatric outpatient study showing that oral frailty was associated with depressive moods in a frailty–clinic population; contributed to the odds ratio (OR)–only backbone.
IN–004	Koh et al. (2026) [95]	Cross–sectional analysis of a nationally representative survey	Yes	Yes	National Korean study showing an association between severe tooth loss and depressive symptoms; quantitative inclusion was based on the separately reported older–adult subgroup aged ≥65 years.
IN–005	Kunrath and Silva (2021) [96]	Prospective longitudinal cohort study	Yes	Yes	Brazilian longitudinal cohort showing that deterioration in oral status, particularly incident tooth loss, was associated with greater depressive symptom burden; contributed to the Risk ratio/prevalence ratio (RR/PR) stream.
IN–006	Kusama et al. (2021) [97]	Prospective longitudinal cohort study	Yes	Yes	Japanese longitudinal cohort showing that fewer remaining teeth were associated with incident depressive symptoms; also relevant to oral function and orofacial appearance pathways.
IN–007	Lin et al. (2022) [98]	Cross–sectional community–based observational study	Yes	Yes	Taiwanese community–based study showing that oral frailty was strongly associated with late–life

					depression in a dose–response pattern; contributed to the odds ratio (OR)–only backbone.
IN–008	Lou et al. (2024) [99]	Cross–sectional population–based observational study (Chinese Longitudinal Healthy Longevity Survey (CLHLS) 2018)	Yes	Yes	Large Chinese older–adult sample showing that better oral–health indicators, especially denture use and more frequent toothbrushing, were associated with lower depressive symptom burden; contributed to the coefficient–based stream.
IN–009	Mariño et al. (2020) [100]	Cross–sectional observational study using data from an ageing cohort wave	Yes	No	Australian community–based old–old sample showing that dentition–related self–perception and concern about mouth appearance were associated with depressive symptoms and psychological well–being; retained for qualitative synthesis only.
IN–010	Matsuyama et al. (2021) [101]	Instrumental–variable observational study using pooled repeated cross–sectional survey data (natural experiment)	Yes	Yes	Instrumental–variable evidence consistent with a possible association between tooth loss and higher depressive symptom burden; only the separately reported older–adult subgroup aged 60–70 years was retained.
IN–011	Miyano et al. (2025) [102]	Longitudinal registry–based observational study	Yes	Yes	Japanese longitudinal study showing that self–reported chewing difficulty was associated with higher odds of newly diagnosed depression; contributed to the odds ratio (OR)–only backbone.
IN–012	Muniz et al. (2021) [103]	Cross–sectional observational study	Yes	Yes	Important null or attenuated finding showing that poorer normative oral conditions or worse oral self–perception were not independently associated with depressive symptoms after adjustment; retained to reduce selective–significance bias.
IN–013	Nakazawa et al. (2024) [104]	Prospective longitudinal cohort study	Yes	Yes	Japanese cohort study showing that severe tooth loss was associated with incident depressive symptoms and that dental prosthesis use attenuated this risk; contributed to the Risk ratio/prevalence ratio (RR/PR) stream.

IN–014	Ohi et al. (2022) [105]	Prospective cohort study with cross–sectional and 4–year longitudinal analyses	Yes	Yes	Japanese cohort evidence showing that impaired oral health–related quality of life (OHRQoL) was associated with both prevalent and incident depressive symptoms.
IN–015	Park et al. (2016) [106]	Cross–sectional, nationally representative observational study	Yes	Yes	Korean community–based study showing that subjective chewing difficulty and pronunciation discomfort were associated with higher odds of depressive symptoms in older adults.
IN–016	Park et al. (2023) [107]	Two–wave longitudinal observational study	Yes	Yes	Korean longitudinal ageing study showing that worsening OHRQoL over time was associated with higher odds of depressive symptoms.
IN–017	Rouxel et al. (2018) [108]	Longitudinal cohort analysis using repeated English Longitudinal Study of Ageing (ELSA) waves	Yes	Yes	English longitudinal study showing that worsening oral impacts on daily life were associated with increased depressive symptoms over time.
IN–018	Skośkiewicz–Malinowska et al. (2018) [109]	Cross–sectional observational study	Yes	Yes	Polish clinically assessed elderly sample showing that tooth–loss burden and multimorbidity were independently associated with greater depression severity.
IN–019	Sun et al. (2022) [110]	Cross–sectional community–based observational study	Yes	Yes	Urban Chinese study showing that better self–rated oral health was associated with lower depressive symptom burden, with dietary satisfaction acting as a partial mediator.
IN–020	Sun et al. (2025) [111]	Cross–sectional observational study	Yes	Yes	Chinese older–adult study showing that oral frailty was independently associated with depressive symptoms, alongside sarcopenia and social frailty.
IN–021	Sung et al. (2025) [112]	Two–wave longitudinal panel study with cross–lagged mediation analysis	Yes	Yes, with caution	Korean longitudinal study showing better oral health was prospectively associated with fewer subsequent depressive symptoms over time and that formal social engagement partially mediated this pathway.

IN–022	Takiguchi et al. (2016) [113]	Cross–sectional observational study nested in a community–based ageing cohort	Yes	Yes	Japanese community–based study showing that objective and subjective oral dryness, especially low unstimulated salivary flow, was associated with depressive symptoms.
IN–023	Tanaka et al. (2026) [114]	Twelve–year prospective community–based cohort study with repeated–measures mixed–effects analysis	Yes	Yes, with caution	Long–term Japanese cohort evidence showing that oral frailty was associated with persistently worse depressive symptom trajectories in older adults.
IN–024	Tay et al. (2025) [115]	Longitudinal observational study using marginal structural models	Yes	Yes	Singaporean longitudinal study showing that chewing disability was prospectively associated with a higher risk of clinically significant depressive symptoms, with part of the effect mediated by loneliness.
IN–025	Wang et al. (2026) [116]	Cross–sectional population–based observational study	Yes	Yes	Large Chinese study showing that stage–specific tooth loss was associated with depressive symptoms, with the strongest adjusted association at the 20–27 tooth–loss stage.
IN–026	Wei et al. (2024) [117]	Cross–sectional nationally representative observational study	Yes	Yes	Nationally representative U.S. study showing that severe impairment of masticatory function, measured by functional tooth units, was associated with higher odds of depression in older adults.
IN–027	Wright et al. (2020) [118]	Cross–sectional analysis nested within a longitudinal ageing cohort	Yes	Yes	Australian cohort–based study showing that limited chewing capacity and untreated dental decay were associated with depressive symptoms in older men.
IN–028	Yamamoto et al. (2017) [119]	Longitudinal population–based cohort study	Yes	Yes	Japanese longitudinal study showing that edentulism and deterioration in chewing ability were prospectively associated with incident depressive symptoms in older adults.
IN–029	Zhang et al. (2025) [120]	Cross–sectional population–based observational study	Yes	Yes	Chinese population–based study showing that poorer oral–health indicators were associated with higher depression levels, with dietary diversity and life satisfaction acting as mediators.

IN–030	Zhang et al. (2021) [121]	Longitudinal population–based cohort study	Yes	Yes	Chinese longitudinal study showing that fewer natural teeth were prospectively associated with higher depressive symptom scores at follow–up, while denture use was associated with lower depressive burden.

Notes: This table summarizes the contribution of each included study to the qualitative and/or quantitative evidence synthesis. "Included in Quantitative Synthesis" indicates entry into at least one prespecified quantitative or structured secondary analytic stream, including the Odds ratio (OR)–only backbone, the Risk ratio/prevalence ratio (RR/PR) stream, or the coefficient–based stream. "Yes, with caution" indicates that the study was retained in a quantitative stream but required cautious interpretation because of limitations in comparability, indirectness, or effect–estimate structure. One study, Mariño et al. (2020) [100], was retained for qualitative synthesis only and did not enter the frozen quantitative workflow. Study–level analytic assignment is reconciled in Supplementary Materials, Table S7.

Table S3. Studies excluded from the qualitative and/or quantitative evidence synthesis, with reasons for exclusion based on prespecified eligibility criteria.

Record ID	Authors (Year)	Study Design	Eligibility Decision	Reason for Exclusion (Criterion and Justification)
EX–001	Abregú–Flores et al. (2025) [122]	Cross–sectional prospective clinical study	Excluded	Directionality mismatch/reverse analytic direction. Participants were classified by depressive symptom status (GDS–15), and oral health indicators were then compared across depression groups.
EX–002	Almohaimeed et al. (2022) [123]	Cross–sectional analysis of NHANES 2015–2016	Excluded	Directionality mismatch/reverse analytic direction. The study treated depressive symptom status as the independent variable and oral health measures (oral condition, oral pain, dental–care access, and oral health–related quality of life) as dependent outcomes.
EX–003	Bafageeh and Loux (2024) [124]	Cross–sectional analysis of NHANES 2017 to March 2020 prepandemic	Excluded	Directionality mismatch/reverse analytic direction. The study modeled depressive symptom severity as the independent variable and multiple oral health outcomes and oral–hygiene behaviors as dependent outcomes.
EX–004	Barbosa et al. (2018) [125]	Cross–sectional multistage–sampled study in Brazilian public healthcare users	Excluded	Population mismatch/insufficiently geriatric sample. Although the analytic direction was eligible, the study included participants aged 15 years and older, had a mean age of 39.88 years, and included only a small minority aged >60 years.
EX–005	Cademartori et al. (2021) [126]	Cross–sectional analysis within a population–based pregnancy cohort	Excluded	Population mismatch/non–geriatric sample. Although the analytic direction was eligible, the study was conducted exclusively in pregnant women from the Pelotas Birth Cohort and therefore did not meet the prespecified older–adult population criterion.
EX–006	Cannon et al. (2022) [127]	Rapid systematic review / conference supplement abstract	Excluded	Publication type mismatch/non–eligible study design. The record was a rapid systematic review presented as a supplement abstract, not an original observational study.
EX–007	Cao et al. (2024) [128]	Cross–sectional survey of rural older adults in China	Excluded	Directionality mismatch/reverse analytic direction. The study treated depressive symptoms/depression status as the independent variable and oral health knowledge, attitudes, and practices as dependent outcomes.
EX–008	Castrejón–Pérez et al. (2022) [129]	Cross–sectional analysis of a community–based older–adult cohort	Excluded	Outcome mismatch/no depressive outcome. The study examined the association between oral conditions and the Frailty Index, with frailty treated as the dependent outcome.

EX–009	Dantas et al. (2020) [130]	Longitudinal quasi–experimental intervention study	Excluded	Design mismatch/non–eligible study design. The study was a quasi–experimental intervention study, whereas the review included observational studies only.
EX–010	D'Avila et al. (2017) [131]	Cross–sectional study of community–dwelling older adults in Brazil	Excluded	Directionality mismatch/reverse analytic direction. The study modeled depressive symptoms as an independent variable and root caries/root–at–risk ratio as the oral health outcome.
EX–011	Delbari et al. (2024) [132]	Cross–sectional cohort–based oral health survey	Excluded	Outcome mismatch/no depressive outcome. The study evaluated oral health status, oral–hygiene behaviors, denture use, edentulism, DMFT, and oral impact on daily performance in adults aged ≥50 years.
EX–012	Figueredo et al. (2020) [133]	Paired controlled clinical trial	Excluded	Design mismatch/non–eligible study design. The study was a controlled clinical trial evaluating the effect of new complete–denture insertion on masticatory ability and oral health–related quality of life in frail and nonfrail elders, whereas the review included observational studies only.
EX–013	Henni et al. (2023) [134]	Scoping review	Excluded	Publication type mismatch/non–eligible study design. The article was a scoping review mapping the literature on oral health and oral health–related quality of life among older adults receiving home health care services, not an original observational study.
EX–014	Hong et al. (2023) [135]	Conference supplement abstract using longitudinal ageing survey data	Excluded	Directionality mismatch/reverse analytic direction. The study examined the impact of depressive symptoms on oral health, treating depressive symptoms as the predictor and deteriorated oral health as the outcome.
EX–015	Hsu et al. (2025) [136]	Cross–sectional population–based study	Excluded	Population mismatch/insufficiently geriatric sample. The study examined adults aged ≥50 years, with a substantial middle–aged component (47.6% aged 50–64 years), and did not report separately analyzable results for a strictly older–adult subgroup.
EX–016	Hussein et al. (2022) [137]	Systematic review with meta–analyses	Excluded	Publication type mismatch/non–eligible study design. The article was a systematic review with meta–analyses, not an original observational study.
EX–017	Ito et al. (2024) [138]	Cross–sectional analysis of baseline ELSI–Brazil data	Excluded	No eligible oral exposure; the study examined the association between depressive symptoms and general self–rated health in Brazilian adults aged ≥50 years without evaluating any prespecified oral exposure such as tooth loss, oral function decline, chewing difficulty, oral frailty, or oral health–related quality of life.

EX–018	Jabr et al. (2024) [139]	Cross–sectional comparative study of prosthetic rehabilitation groups	Excluded	Outcome mismatch/no depressive outcome. The study compared completely edentulous older adults rehabilitated with different prosthetic treatments and evaluated masticatory performance, oral health–related quality of life, self–perception of oral health, and nutritional status, but it did not report depression diagnosis, depressive symptoms, incident depression, or depression severity as an eligible outcome.
EX–019	Jilili et al. (2024) [140]	Longitudinal community cohort study	Excluded	Outcome mismatch/no depressive outcome. The study examined the association between oral status (tooth loss and denture use) and healthy ageing over four years in Chinese older adults.
EX–020	Kamiya et al. (2016) [141]	Experimental functional neuroimaging study with within–subject denture/tooth–loss comparisons	Excluded	Outcome mismatch/no depressive outcome. The study evaluated prefrontal activity, chewing performance, occlusal force, and masticatory muscle activity in partially edentulous older adults under denture–versus–tooth–loss conditions, but it did not report depression diagnosis, depressive symptoms, incident depression, or depression severity as an eligible outcome.
EX–021	Kimble et al. (2023) [142]	Prospective longitudinal analysis of two older–adult cohorts	Excluded	Outcome mismatch/no depressive outcome. The study examined prospective associations between oral health markers and progression of physical frailty/severe frailty in older adults from the United Kingdom and the United States.
EX–022	Kossioni et al. (2018) [143]	Special article / practice guideline paper	Excluded	Publication type mismatch/non–eligible study design. The article was a practice guideline/special article providing recommendations for physicians on promoting oral health in frail older adults, not an original observational study.
EX–023	Maki et al. (2024) [144]	Cross–sectional community–based study	Excluded	Directionality mismatch/reverse analytic direction, together with population mismatch. The study modeled oral dysfunction as the dependent variable and examined depressive mood as an explanatory predictor.
EX–024	Manica et al. (2024) [145]	Cross–sectional analysis of baseline ELSI–Brazil data	Excluded	Directionality mismatch/reverse analytic direction. The study treated psychosocial aspects, including depressive symptoms, as predictors and self–reported oral health as the outcome.
EX–025	Marques et al. (2017) [146]	Longitudinal cohort study of older adults in southern Brazil	Excluded	Outcome mismatch/no depressive outcome. The study examined the 8–year incidence of edentulism and its predictors in older adults.
EX–026	Mun et al. (2019) [147]	Cross–sectional study of older adults with suspected dementia in South Korea	Excluded	Directionality mismatch/reverse analytic direction. The study modeled oral health–related quality of life (GOHAI) as the outcome and treated geriatric depression, oral dryness, oral health state, cognitive function, and activities of daily living as explanatory variables.

EX–027	Nihtilä et al. (2019) [148]	Controlled intervention study with tailored xerostomia and nutritional interventions	Excluded	Design mismatch/non–eligible study design. The study was an intervention study evaluating tailored xerostomia care and nutritional intervention among home–care clients aged ≥75 years, whereas the review included observational studies only.
EX–028	Ortíz–Barrios et al. (2019) [149]	Cross–sectional study with latent class analysis in community–dwelling older adults	Excluded	Outcome mismatch/no depressive outcome. The study examined the impact of poor oral health on oral health–related quality of life (OHRQoL/GOHAI) in older adults using latent oral–health classes.
EX–029	Palomer et al. (2024) [150]	Cross–sectional population–based study	Excluded	Population mismatch/insufficiently geriatric sample. The study analyzed adults aged ≥18 years from the Chilean National Health Survey and did not report a separately analyzable older–adult subgroup.
EX–030	Park et al. (2022) [151]	Cross–sectional path–analysis study of community–dwelling older adults in Korea	Excluded	Insufficient quantitative reporting for robust effect extraction. The study was conceptually relevant and involved older adults aged ≥65 years, but the available record provided only abstract–level standardized path coefficients and p–values without 95% confidence intervals, standard errors, or full model statistics required for reliable inclusion in the quantitative workflow and meta–analytic dataset.
EX–031	Petrovsky et al. (2019) [152]	Two–wave epidemiological observational study	Excluded	Outcome mismatch/no depressive outcome. The study examined associations between oral health symptoms (tooth and gum symptoms) and changes in cognitive function among community–dwelling older Chinese adults in the United States.
EX–032	Qu et al. (2024) [153]	Cross–sectional population–based study with propensity–score matching and causal mediation analysis	Excluded	Population mismatch/insufficiently geriatric sample. The study was conducted in a middle–aged and older population from CHARLS and did not report a separately analyzable older–adult subgroup for the primary oral exposure–depression association.
EX–033	Rech et al. (2019) [154]	Cross–sectional study nested within a cohort of community–dwelling older adults	Excluded	Directionality mismatch/reverse analytic direction. The study investigated factors associated with xerostomia and hyposalivation, treating depressive symptoms as an explanatory variable and xerostomia/salivary hypofunction as the oral outcome.
EX–034	Ribeiro et al. (2016) [155]	Cross–sectional population–based study of older adults in southern Brazil	Excluded	Directionality mismatch/reverse analytic direction. The study modeled edentulism, severe tooth loss, and lack of functional dentition as dependent oral outcomes and treated depression as an explanatory variable in the analytical models.

EX–035	Rivoli et al. (2025) [156]	Observational longitudinal study of older adults attending primary care	Excluded	No eligible oral exposure/outside review scope. The study examined 12–month changes in depressive symptoms and cognitive function in Brazilian older adults using GDS–15 and MMSE, with general clinical predictors such as medication use, self–perceived health, polypharmacy, and falls.
EX–036	Saarela et al. (2019) [157]	Cross–sectional study in long–term care settings	Excluded	Outcome mismatch/no depressive outcome. The study examined the association between oral symptom burden (chewing problems, swallowing difficulties, dry mouth) and generic health–related quality of life (15D) among older residents in long–term care.
EX–037	Seki et al. (2025) [158]	Prospective cohort study of community–dwelling older adults	Excluded	Outcome mismatch/no depressive outcome. The study examined longitudinal associations of remaining natural teeth, functional teeth, and occlusal force with incident functional disability and all–cause mortality among older Japanese adults with fewer than 20 remaining teeth.
EX–038	Singh et al. (2024) [159]	Cross–sectional analysis of NHANES 2015–2018	Excluded	Directionality mismatch/reverse analytic direction. The study modeled untreated dental decay as the dependent oral outcome and examined associations with different disability types and related factors rather than evaluating oral structural or functional impairment as the exposure and depressive outcome as the endpoint.
EX–039	Takeuchi et al. (2026) [160]	Longitudinal cohort study based on repeated online survey waves	Excluded	Population mismatch/insufficiently geriatric sample. The study analyzed Japanese adults aged ≥20 years without baseline depression from JACSIS and did not report a separately analyzable older–adult subgroup for the oral exposure–depression association.
EX–040	Tian et al. (2025) [161]	Cross–sectional multicenter observational study	Excluded	Population mismatch/insufficiently geriatric sample. The study included adults aged ≥50 years from the WCHAT cohort and did not report a separately analyzable older–adult subgroup for the main oral exposure–depressive symptoms association.
EX–041	Zwick et al. (2023) [162]	Longitudinal population–based cohort study	Excluded	Population mismatch/insufficiently geriatric sample. The study analyzed adults aged ≥50 years from ELSA and did not report a separately analyzable older–adult subgroup for the main oral exposure–depressive symptoms association.

Notes: This table summarizes reports excluded after full–text assessment according to the prespecified eligibility framework. Exclusion reasons are reported at the report level to preserve transparency and auditability, even when multiple reports addressed related domains. Each record was assigned a primary exclusion rationale aligned with the final adjudicated reason for non–inclusion in the review corpus.

Table S4. Included studies and their principal methodological, population, exposure, outcome, and selected effect-estimate characteristics.

Record ID	Authors (Year), Country	Study Design	Population Characteristics [Sex; Age]	Analytic Sample	Exposure Contrast/Oral Indicator	Depressive Outcome and Selected Model/Effect Estimate (95% CI)	Clinical Context/Relevant Subgroup	Summary Interpretation
IN–001	Hajek et al. (2023), Multinational Europe/Israel [92]	Cross–sectional, population–based, multicountry observational study (Survey of Health, Ageing and Retirement in Europe (SHARE) wave 5)	Male 43.9%; female 56.1%; mean age 66.6 (SD 10.2) years	Total analytic sample N = 62,358; depressive model in total sample n = 61,972; missing–teeth model n = 45,984	Presence of missing natural teeth vs absence	Depressive symptoms (Euro–D continuous score); adjusted beta = 0.210 (95% CI: 0.171 to 0.249)	General community–dwelling ageing population; no clinical depression subgroup	Poorer oral status was associated with higher depressive symptom burden, whereas more complete replacement of missing natural teeth was associated with lower depressive symptoms.
IN–002	Kaur et al. (2024), India [93]	Cross–sectional, institution–based observational study	Male 35.6%; female 64.4%; age groups: 60–70 years 69.0%, 70–80 years 26.4%, >80 years 4.6%; mean age not reported	N = 500	Missing–teeth score, per 1–unit increase	Depression severity (PHQ–9 continuous score); adjusted beta = 0.122 (95% CI: 0.107 to 0.129)	Institutionalized older adults residing in three old–age homes	Greater missing–tooth burden was associated with higher depression severity in institutionalized older adults.
IN–003	Kawamura et al. (2026), Japan [94]	Cross–sectional outpatient observational study	Sex not reported in available file; mean age 78.4 (SD 6.4) years;	N = 337	Oral frailty (OF–5 ≥2) vs no oral frailty	Depressive moods (GDS–15 ≥5); adjusted OR = 2.13 (95% CI: 1.27 to 3.55)	Frailty outpatient clinic population	Oral frailty was associated with approximately doubled odds of depressive moods in older outpatients.

			all participants aged ≥65 years					
IN–004	Koh et al. (2026), South Korea [95]	Cross–sectional analysis of a nationally representative survey	Overall sample: male 42.8%, female 57.2%, aged ≥30 years; eligible older–adult subgroup aged ≥65 years	Overall N = 9,166; eligible older–adult subgroup n = 1,938	Fewer than 12 remaining natural teeth vs 12 or more	Depressive symptoms (binary); stratified multivariable OR = 1.49 (95% CI: 1.11 to 2.02) in adults aged ≥65 years	Older–adult subgroup extracted from a broader adult Korean survey	Severe tooth loss was associated with higher odds of depressive symptoms in the eligible older–adult subgroup.
IN–005	Kunrath and Silva (2021), Brazil [96]	Prospective longitudinal cohort study	Male 26.2%; female 73.8%; age groups at follow–up: 60–69 years 28.4%, 70–79 years 49.4%, ≥80 years 22.2%; mean age not reported	Baseline N = 439; follow–up analytic sample N = 164	Incident tooth loss between 2009 and 2015 vs same number of teeth	Depressive symptoms (GDS–15 ≥5); adjusted PR = 1.86 (95% CI: 1.01 to 3.53)	Community–dwelling older adults registered with family health units	Incident tooth loss was associated with greater risk of depressive symptoms at follow–up.
IN–006	Kusama et al. (2021), Japan [97]	Prospective longitudinal cohort study	Male 48.0%; female 52.0%; mean age 72.7 (SD 5.5) years	N = 8,875	≤19 remaining teeth vs ≥20 remaining teeth	Incident depressive symptoms (GDS–15 ≥5 at follow–up); adjusted OR = 1.30 (95% CI: 1.12 to 1.51)	Community–dwelling older adults aged ≥65 years, free of depressive symptoms at baseline	Fewer remaining teeth were associated with higher odds of incident depressive symptoms over follow–up.
IN–007	Lin et al. (2022), Taiwan [98]	Cross–sectional, community–based observational study	Male 28.6%; female 71.4%; age groups: ≤74 years 58.1%, ≥75 years 41.9%; mean	N = 1,100	Oral frailty vs non–oral frailty	Late–life depression (GDS–15 >5); adjusted OR = 4.89 (95% CI: 2.02 to 11.80)	Community–dwelling older adults aged ≥65 years	Oral frailty was strongly associated with late–life depression in a dose–response pattern.

			age not reported					
IN–008	Lou et al. (2024), China [99]	Cross–sectional, population–based observational study (Chinese Longitudinal Healthy Longevity Survey (CLHLS) 2018)	Male 45.9%; female 54.1%; mean age 83.1 (SD 11.2) years	N = 8,442	Denture use vs no denture use	Depressive symptoms (CESD–10 continuous score); total effect beta = –0.341 (95% CI: –0.505 to –0.178)	General older–adult population from CLHLS	Better oral–health indicators, especially denture use, were associated with lower depressive symptom burden.
IN–009	Mariño et al. (2020), Australia [100]	Cross–sectional observational study using data from an ageing cohort wave	Male 52.0%; female 48.0%; mean age 83.9 (SD 3.6) years; age range 79–96 years	N = 201	Concern about appearance of mouth/teeth	Depressive symptoms score; multivariable linear regression coefficient B = 0.93; 95% CI not reported	Independent–living old–old adults in metropolitan Melbourne	Oral self–perception, especially concern about mouth appearance, was associated with higher depressive symptom burden; exact variance estimates were insufficient for pooling.
IN–010	Matsuyama et al. (2021), USA [101]	Instrumental–variable observational study using pooled repeated cross–sectional survey data (natural experiment)	Overall sample: male 38.7%, female 61.3%, mean age 50.0 (SD 10.8) years, range 28–70 years; eligible subgroup aged 60–70 years	Overall N = 169,061; eligible older–adult subgroup sample size not reported	Effect per additional lost tooth in adults aged 60–70 years	Depressive symptoms (PHQ–8 total score); 2SLS coefficient = –0.055 (95% CI: –1.019 to 0.909)	Older–adult subgroup extracted from a broader U.S. adult sample	In the eligible older–adult subgroup, the estimate was imprecise and compatible with no clear independent association.

IN–011	Miyano et al. (2025), Japan [102]	Longitudinal registry–based observational study	Male 39.3%; female 60.7%; mean age 71.5 (SD 4.8) years	N = 23,477	Poor chewing ability vs good chewing ability	Newly diagnosed depression; adjusted OR = 1.26 (95% CI: 1.00 to 1.58)	Community–dwelling older adults from 17 Japanese municipalities; participants with prior depression excluded at baseline	Poor chewing ability was associated with higher odds of newly diagnosed depression during follow–up.
IN–012	Muniz et al. (2021), Brazil [103]	Cross–sectional observational study	Female 77.6%; male 22.4%; mean age 69.0 (SD 6.14) years; range 60–90 years	N = 250	Edentulism, yes vs no	Depressive symptoms (GDS–15 >5); adjusted OR = 1.296 (95% CI: 0.725 to 2.318)	Non–institutionalized older adults enrolled at a geriatric reference center	Normative oral conditions were not independently associated with depressive symptoms after multivariable adjustment.
IN–013	Nakazawa et al. (2024), Japan [104]	Prospective longitudinal cohort study	Male 49.7%; female 50.3%; mean age 72.8 (SD 5.4) years	N = 50,169	0–9 remaining teeth without dental prostheses vs ≥20 teeth	Incident depressive symptoms (GDS–15 >4 at follow–up); adjusted RR = 1.27 (95% CI: 1.04 to 1.56)	Community–dwelling older adults aged ≥65 years without depressive symptoms at baseline	Severe tooth loss was associated with higher risk of incident depressive symptoms, with attenuation among prosthesis users.
IN–014	Ohi et al. (2022), Japan [105]	Prospective cohort study with cross–sectional and 4–year longitudinal analyses	Mean age 67.8 (SD 7.2) years; all participants aged ≥55 years; sex distribution not fully reported in article tables	Cross–sectional n = 669; longitudinal n = 296	Impaired oral health–related quality of life vs no impairment	Development of depressive symptoms within 4 years; adjusted OR = 6.00 (95% CI: 1.38 to 26.09)	Community–dwelling older Japanese adults; longitudinal analysis restricted to participants free of depressive symptoms at baseline	Impaired oral health–related quality of life was strongly associated with both prevalent and incident depressive symptoms.
IN–015	Park et al. (2016), South Korea [106]	Cross–sectional, nationally representative	Male 40.6%; female 59.4%; all participants aged ≥65 years;	N = 50,694	Chewing difficulty vs no chewing difficulty	Depressive symptoms (CES–D ≥16); sex–combined adjusted OR = 1.486 (95% CI: 1.396 to 1.581)	Community–dwelling Korean older adults	Subjective chewing difficulty was associated with higher odds of depressive symptoms.

		observational study	mean age not reported					
IN–016	Park et al. (2023), South Korea [107]	Two–wave longitudinal observational study	Weighted sample: male 42.9%, female 57.1%; age groups: 65–69 years 35.3%, 70–74 years 25.3%, 75–79 years 20.7%, ≥80 years 18.6%	N = 3,615	Worse oral physical function score, inverse–coded from reported change	Depressive symptoms in 2020 (CES–D10 ≥4); adjusted OR = 1.111 (95% CI: 1.053 to 1.176)	Community–dwelling Korean older adults aged ≥65 years	Worsening oral–health–related quality of life, especially oral physical function, was associated with higher odds of depressive symptoms.
IN–017	Rouxel et al. (2018), United Kingdom [108]	Longitudinal cohort analysis using repeated English Longitudinal Study of Ageing (ELSA) waves	Later–life population from ELSA; sex distribution not reported in available article pages; age 50+ years	Primary depressive–symptoms change model N = 4,807; eligible at both waves N = 6,294	Incident oral impact on daily life vs no change	Incident depressive symptoms; adjusted OR = 1.60 (95% CI: 1.20 to 2.15)	Non–institutionalized older adults living in England	Worsening oral impacts on daily life were associated with increased depressive symptoms over time.
IN–018	Skośkiewicz–Malinowska et al. (2018), Poland [109]	Cross–sectional observational study	Male 36.0%; female 64.0%; mean age 74.4 (SD 7.4) years; age range 65–99 years	N = 500	Number of extracted teeth, per additional tooth	Depression severity (PHQ–9 total score); adjusted beta = 0.111 (95% CI: 0.105 to 0.116)	Urban older adults from Wroclaw	Greater extracted–tooth burden was independently associated with higher depression severity.
IN–019	Sun et al. (2022), China [110]	Cross–sectional, community–based observational study	Male 38.9%; female 61.1%; mean age 71.33 (SD 7.03) years	N = 781	Self–rated oral health, per 1–category increase from terrible to very good	Depressive symptoms score (CES–D–10); total effect B = –0.22 (95% CI: –0.44 to –0.01)	Urban older adults from Tianjin and Shijiazhuang	Better self–rated oral health was associated with lower depressive symptom burden.

IN–020	Sun et al. (2025), China [111]	Cross–sectional observational study	Male 46.5%; female 53.5%; age groups: 60–69 years 54.5%, 70–79 years 35.3%, ≥80 years 10.2%; mean age not reported	N = 1,453	Oral frailty vs no oral frailty	Depressive symptoms (PHQ–9); adjusted OR = 3.136 (95% CI: 2.430 to 4.046)	Community–dwelling Chinese older adults aged ≥60 years	Oral frailty was independently associated with markedly higher odds of depressive symptoms.
IN–021	Sung et al. (2025), South Korea [112]	Two–wave longitudinal panel study with cross–lagged mediation analysis	Male 41.5%; female 58.5%; mean age 75.12 (SD 6.85) years; all participants aged ≥65 years	N = 3,860	Better oral health measured with the Geriatric Oral Health Assessment Index (GOHAI)	Depressive symptoms in 2020 (CES–D–10 score); standardized beta = –0.04 (95% CI: –0.06 to –0.03)	Community–dwelling Korean older adults	Better oral health was prospectively associated with fewer subsequent depressive symptoms although the reverse path from depressive symptoms to later oral health was stronger.
IN–022	Takiguchi et al. (2016), Japan [113]	Cross–sectional observational study nested in a community–based ageing cohort	Male 53.8%; female 46.2%; participants were approximately 77–78 years old; mean age not reported	N = 351	Low unstimulated salivary flow rate (<0.2 mL/min) vs normal flow	High depressive–symptom burden (GHQ–30 ≥7); adjusted OR = 2.1 (95% CI: 1.2 to 3.9)	Community–dwelling older Japanese adults	Objective oral dryness, especially low unstimulated salivary flow, was associated with higher odds of depressive symptoms.
IN–023	Tanaka et al. (2026), Japan [114]	Twelve–year prospective community–based cohort study with repeated–measures mixed–effects analysis	Male 50.5%; female 49.5%; mean age 72.7 (SD 5.5) years; all participants aged ≥65 years	N = 1,679 participants; 7,048 observations across 7 waves	Baseline oral frailty vs non–oral frailty	Trajectory of depressive symptoms (GDS–5 score); adjusted beta = 0.126 (95% CI: 0.062 to 0.189)	Community–dwelling older adults from Kashiwa, Japan	Oral frailty was associated with persistently higher depressive symptom burden over 12 years.

IN–024	Tay et al. (2025), Singapore [115]	Longitudinal observational study using marginal structural models	Male 46.5%; female 53.5%; mean age 70.4 (SD 7.1) years; all participants aged ≥60 years and free of clinically significant depressive symptoms at baseline	N = 1,277	Chewing disability vs no chewing disability	Clinically significant depressive symptoms over 6 years; total cumulative effect RR = 1.48 (95% CI: 1.15 to 1.82)	Older adults in Singapore from the PHASE study	Chewing disability was prospectively associated with a higher risk of developing clinically significant depressive symptoms over time.
IN–025	Wang et al. (2026), China [116]	Cross–sectional, population–based observational study	Male 45.64%; female 54.36%; age groups: 65–79 years 41.1%, ≥80 years 58.9%; mean age not reported	N = 8,413	20–27 tooth loss vs 0–8 tooth loss	Depressive symptoms (CES–D–10 ≥10); adjusted OR = 1.337 (95% CI: 1.106 to 1.617)	Chinese older adults aged >65 years	Tooth loss was associated with depressive symptoms in a stage–like manner, with the strongest adjusted association at the 20–27 tooth–loss stage.
IN–026	Wei et al. (2024), USA [117]	Cross–sectional, nationally representative observational study	Male 55.1%; female 44.9%; age groups: 60–65 years 36.7%, 66–70 years 22.0%, 71–75 years 16.8%, 76–80 years 24.6%; mean age not reported	N = 5,764	0–3 functional tooth units vs 10–12 functional tooth units	Depression (PHQ–9 ≥10); adjusted OR = 1.819 (95% CI: 1.157 to 2.858)	U.S. community–dwelling older adults aged >60 years	Severe impairment of masticatory function measured by functional tooth units was associated with higher odds of depression.
IN–027	Wright et al. (2020),	Cross–sectional	Male 100%; cohort	Primary oral–health	Limited chewing capacity vs no	Depressive symptoms (GDS–15 ≥5); adjusted PR	Community–dwelling older	Limited chewing capacity was independently associated with

	Australia [118]	analysis nested within a longitudinal ageing cohort	comprised men aged ≥70 years at study entry and older at year–8 follow–up; mean age not reported	analytic sample N = 589	chewing problems	= 1.93 (95% CI: 1.34 to 2.79)	Australian men from the Concord Health and Ageing in Men Project	depressive symptoms in older men.
IN–028	Yamamoto et al. (2017), Japan [119]	Longitudinal population–based cohort study	Male/female distribution not reported in available article pages; all participants aged ≥65 years	Overall cohort N = 14,279; logistic–regression sample after multiple imputation ranged from 10,424 to 10,461 after excluding baseline depressive symptoms	No teeth vs ≥20 teeth	Incident depressive symptoms (GDS ≥5 at follow–up); adjusted OR = 1.28 (95% CI: 1.03 to 1.60)	Community–dwelling older Japanese adults without depressive symptoms at baseline	Having no teeth was prospectively associated with later depressive symptoms and supported a longitudinal structural oral–health signal.
IN–029	Zhang et al. (2025), China [120]	Cross–sectional, population–based observational study	Male 45.5%; female 54.5%; mean age 83.12 (SD 11.32) years	N = 10,010	Remaining teeth, per unit increase	Depression level (CESD–10 continuous score); total effect = –0.018 (95% CI: –0.031 to –0.005)	Community–dwelling older Chinese adults aged ≥60 years	Better oral status was associated with lower depression levels, with mediation through dietary diversity and life satisfaction.
IN–030	Zhang et al. (2021), China [121]	Longitudinal population–based cohort study	Male 46.99%; female 53.01%; mean age 81.29 (SD 10.32) years	N = 3,310	1–9 natural teeth vs ≥20 natural teeth	Depressive symptom score at follow–up; adjusted coefficient = 0.59 (95% CI: 0.30 to 0.65)	Community–dwelling Chinese older adults aged ≥65 years	Fewer teeth were associated with higher depressive symptom scores at 4–year follow–up, whereas denture

use was associated with lower depressive burden.

Notes: This table summarizes the principal methodological, population, exposure, outcome, and selected effect–estimate characteristics of the 30 studies included in the final review corpus. “Analytic Sample” refers to the sample contributing to the reported principal estimate, which may differ from the full recruited cohort or source population. Where studies reported multiple eligible oral–depression estimates, only the principal estimate most relevant to the study–level descriptive profile is presented here; full analytic selection, poolability, and stream assignment are reported in Tables S6–S10. Age expressions, study–design labels, and effect–estimate wording have been standardized for consistency across rows. Major acronyms are defined as follows: SHARE, Survey of Health, Ageing and Retirement in Europe; CLHLS, Chinese Longitudinal Healthy Longevity Survey; ELSA, English Longitudinal Study of Ageing; OHRQoL, oral health–related quality of life; GOHAI, Geriatric Oral Health Assessment Index; GDS, Geriatric Depression Scale; PHQ–9, Patient Health Questionnaire–9; CES–D/CESD–10, Center for Epidemiologic Studies Depression Scale; GHQ–30, 30–item General Health Questionnaire; 2SLS, two–stage least squares. Selected estimates are reported as originally expressed in the source studies or in the frozen analytical workflow used for this review. All effect estimates reported in Table S4 were cross–checked against the original publications and the final meta–analytic dataset to ensure consistency.

Table S5. Study–level Newcastle-Ottawa Scale (NOS) assessments of included studies (Panel A) and body-level GRADE certainty judgments for the synthesized evidence streams (Panel B).

Panel A. Study-level Newcastle–Ottawa Scale (NOS) assessment of included studies.

Record ID	Authors (Year)	NOS Selection Domain (0–4 Stars; Brief Justification)	NOS Comparability Domain (0–2 Stars; Brief Justification)	NOS Outcome/Exposure Domain (0–3 Stars; Brief Justification)	Total NOS Score (0–9 Stars)	NOS Methodological Quality Category	Brief Overall NOS Interpretation
IN–001	Hajek et al. (2023) [92]	3; Very large population–based SHARE sample with clear exposure specification and multicountry coverage, but oral exposure was self–reported and detailed non–response analysis was not fully reported.	2; Adjusted for age, sex, marital status, education, household income, chronic conditions, and country.	3; Depressive symptoms were assessed with the validated Euro–D instrument and procedures were applied consistently across participants.	8	High	Methodologically strong for a cross–sectional study, with only minor deductions for self–reported exposure and cross–sectional temporality.
IN–002	Kaur et al. (2024) [93]	2; Clearly defined older population (aged ≥60 years) sampled from three old–age homes, but representativeness was limited by institution–based sampling and incomplete age/response reporting.	1; Multiple regression was reported, but covariate adjustment and model–building details were incompletely described.	3; Oral variables were clinically assessed and depression was measured with PHQ–9 using consistent procedures.	6	Moderate	Clinically relevant but methodologically limited by institution–based sampling, cross–sectional design, and incomplete reporting.
IN–003	Kawamura et al. (2026) [94]	2; Clearly defined older outpatient population from a frailty clinic, but representativeness was	1; Logistic regression adjustment was reported, but the full covariate set was not	2; Depression and oral frailty were assessed with named instruments (GDS–15 and OF–5), but	5	Moderate	Tentatively usable and clinically relevant, but quality scoring should be confirmed against the complete full text.

		limited to a specialty setting and recruitment details were incompletely reported.	sufficiently detailed in the available file.	ascertainment details and missing–data handling were not fully verifiable from the available record.			
IN–004	Koh et al. (2026) [95]	3; Strong national survey framework with calibrated dental examinations and large sample size, but subgroup extraction from a broader adult sample was required for protocol alignment.	2; Adjusted for a substantial set of sociodemographic, behavioural, metabolic, and prosthetic–need covariates.	2; Oral exposure was clinically ascertained, but depressive symptoms were assessed by a simplified self–report outcome rather than a full validated scale in the final model.	7	High	Methodologically solid for a cross–sectional national survey, with deductions for subgroup–based geriatric eligibility and simplified outcome ascertainment.
IN–005	Kunrath and Silva (2021) [96]	3; Randomly selected older adults from family health units with clearly defined inclusion criteria, but the cohort was local and follow–up attrition was substantial.	2; Hierarchical multivariable modelling adjusted for key sociodemographic, health, medication, clinical oral, and self–perceived oral health variables.	2; Outcome assessment used validated GDS–15 and repeated oral assessments were performed by trained/calibrated examiners, but follow–up completion was limited.	7	High	Methodologically strong and especially valuable because of the longitudinal design, but not maximal because of follow–up loss and possible attrition bias.
IN–006	Kusama et al. (2021) [97]	4; Clearly defined community–dwelling older adults aged ≥65 years; baseline depressive cases were excluded and exposure was explicitly defined.	2; Main models adjusted for key demographic, socioeconomic, behavioural, household, and health confounders.	3; Outcome assessment used validated GDS–15, follow–up duration was adequate, and sensitivity analyses included multiple imputation and complete–case comparison.	9	High	One of the strongest studies in the review, with clear temporality, robust adjustment, and explicit sensitivity analyses.
IN–007	Lin et al. (2022) [98]	3; Clearly defined older population, stratified cluster sampling, and	2; Multivariable models adjusted for major demographic	3; Late–life depression was measured with GDS–15 and oral conditions	8	High	Methodologically strong for a cross–sectional study, with main

		high response rate, but the sample was regional rather than nationally representative.	and clinical confounders including frailty and sarcopenia.	were assessed using structured instruments and dental examination.			limitations being lack of temporality and regional sampling.
IN–008	Lou et al. (2024) [99]	4; Clearly defined older population (aged ≥65 years), large national CLHLS sample, and transparent inclusion/exclusion flow.	2; Adjusted for a broad set of sociodemographic, behavioural, and health–status covariates.	3; Depressive symptoms were measured with CESD–10 and oral exposures were consistently assessed; mediation procedures were clearly reported.	9	High	Methodologically strong for a cross–sectional observational study, with main limitations stemming from temporality rather than execution quality.
IN–009	Mariño et al. (2020) [100]	2; Clearly older community–dwelling population aged ≥79 years, but the sample was small and derived from long–term survivors of an earlier cohort.	1; Multivariable linear regression included several covariates, but stepwise modelling and incomplete reporting limited confidence in comparability control.	3; Validated psychological instruments and structured self–report procedures were used consistently.	6	Moderate	Useful for qualitative evidence, but limited by cross–sectional design, survivor bias, and incomplete reporting of effect precision.
IN–010	Matsuyama et al. (2021) [101]	3; Very large U.S. population–based sample from BRFSS with transparent sampling source and clear restrictions, but the main sample was not exclusively older–adult and subgroup–specific size for ages 60–70 years was not reported.	2; Strong quasi–experimental instrumental–variable strategy with adjustment for year of birth, survey wave, sex, and state of residence.	2; Depression was assessed with PHQ–8 and exposure was self–reported tooth loss, but the review–relevant effect was subgroup–derived.	7	High	Methodologically strong for observational evidence because of the natural–experiment design, but not maximal because of subgroup–based geriatric eligibility and indirectness.

IN–011	Miyano et al. (2025) [102]	3; Clearly defined older population (aged ≥65 years), large linked municipal dataset, and exclusion of participants with prior depression.	2; Adjusted for major demographic and health–related confounders including age, sex, BMI, comorbidity burden, smoking, alcohol, and sleep.	3; Outcome ascertainment through linked medical claims data was strong and follow–up was adequate for incident diagnosis detection.	8	High	Methodologically strong longitudinal study, with minor limitations related mainly to self–reported chewing exposure and regional rather than national coverage.
IN–012	Muniz et al. (2021) [103]	2; Clearly defined older–adult sample (aged ≥60 years) with standardized inclusion/exclusion criteria and calibrated oral examination, but convenience sampling from a single center limited representativeness.	1; Multivariable adjustment was partial and did not cover the full likely confounder structure.	3; Outcome was measured with validated GDS–15 and oral exposures were clinically assessed by trained/calibrated examiners.	6	Moderate	Moderate methodological quality overall: good outcome and exposure assessment, but limited by convenience sampling, cross–sectional design, and partial confounder control.
IN–013	Nakazawa et al. (2024) [104]	4; Clearly defined cohort of independent older adults aged ≥65 years; outcome absent at baseline and exposure categories explicitly defined before follow–up.	2; Fully adjusted models included major sociodemographic, behavioural, medical, household, and cognitive confounders.	2; Outcome assessment used validated GDS–15 and follow–up was adequate, but exposure was self–reported and follow–up completeness was imperfect.	8	High	Methodologically strong longitudinal cohort study, with only modest limitations related to self–reported exposure and follow–up attrition.
IN–014	Ohi et al. (2022) [105]	3; Community–dwelling older adults were clearly defined and baseline depressive cases were excluded from the	2; Fully adjusted models included major demographic, behavioural, medical, cognitive, and oral–	2; Outcome assessment used validated SDS and follow–up was adequate, but only a reduced subset of baseline participants	7	High	Valuable because of the longitudinal design and baseline depression exclusion, but not maximal because of attrition, small incident–case counts, and some age–definition indirectness.

		longitudinal analysis, but follow–up retention was limited.	health–related covariates.	contributed to the longitudinal analysis.			
IN–015	Park et al. (2016) [106]	4; Nationally representative elderly sample from the Korean Community Health Survey with large sample size and clearly defined age eligibility.	2; Adjusted models included major sociodemographic, health, stress, behavioural, and sleep–related covariates.	2; Depressive symptoms were measured with validated CES–D, but oral exposures were self–reported and the design was cross–sectional.	8	High	Methodologically strong for a cross–sectional national survey, with main limitations being self–reported oral function and lack of temporality.
IN–016	Park et al. (2023) [107]	3; Clearly defined older–adult cohort (aged ≥65 years), nationally based longitudinal ageing dataset, and transparent exclusion flow.	2; Adjusted models included major sociodemographic, health, functional, and oral covariates.	2; Depressive symptoms were measured with validated CES–D10 and the same oral–health instrument was used at both waves, but incident depression was not isolated by baseline exclusion.	7	High	Methodologically useful and informative for longitudinal oral–health change, but not maximally robust because of self–reported exposure and reporting limitations.
IN–017	Rouxel et al. (2018) [108]	3; Large national ageing cohort with repeated follow–up, but attrition and complete–case analysis reduced the analytical sample and the cohort was not restricted to a strict geriatric cutoff.	2; Fully adjusted models accounted for broad time–constant and time–varying confounders.	2; Depressive symptoms were measured with a validated CES–D short form and follow–up was adequate, but oral exposure measures were self–reported and loss to follow–up remained substantial.	7	High	Methodologically strong longitudinal evidence, but not maximal because of attrition, self–reported exposure, and age–frame indirectness.
IN–018	Skośkiewicz–Malinowska et al. (2018) [109]	2; Clearly older population and reasonable sample size, but voluntary urban	1; Multiple regression retained key predictors, but confounder control	3; Oral conditions were clinically measured by calibrated examiners and	6	Moderate	Useful clinically assessed evidence, but limited by cross–sectional design and local volunteer sampling.

		recruitment limited representativeness.	was limited and based on stepwise modelling.	depression was measured with PHQ–9.			
IN–019	Sun et al. (2022) [110]	3; Clearly defined older population (aged ≥60 years), face–to–face data collection, and calibration to local demographic structure, but the sample was urban and local rather than national.	2; Models adjusted for major sociodemographic and health–related confounders.	3; Depressive symptoms were measured with CES–D–10 and mediation/moderated mediation analyses were reported with confidence intervals.	8	High	Methodologically solid for a cross–sectional observational study, with main limitations being lack of temporality and local self–reported exposure assessment.
IN–020	Sun et al. (2025) [111]	2; Clearly older population (aged ≥60 years), but sampling appeared local/provincial rather than nationally representative.	2; Multivariable models adjusted for several relevant demographic and health–related covariates.	3; Depression was measured with PHQ–9 and oral frailty with OFI–8 using standardized procedures.	7	High	Methodologically solid for a cross–sectional observational study, with the main limitations being lack of temporality and non–national sampling.
IN–021	Sung et al. (2025) [112]	3; Clearly defined older–adult population (aged ≥65 years) from a national longitudinal ageing study with two repeated waves.	2; Cross–lagged models adjusted for a broad set of baseline and time–varying sociodemographic and health–related covariates.	2; Repeated assessment is a strength, but the study did not isolate an incident–depression cohort and oral health was self–reported.	7	High	Methodologically strong for longitudinal panel evidence, but not maximal because of self–reported exposure and indirect comparability of standardized cross–lagged coefficients.
IN–022	Takiguchi et al. (2016) [113]	3; Clearly defined elderly community cohort with random recruitment and standardized examinations, but long exclusions before final	1; Multivariable adjustment was performed, but the confounder set remained relatively narrow.	3; Oral exposures were clinically measured by trained/calibrated dentists, salivary flow was objectively assessed, and GHQ–30 was applied consistently.	7	High	Strong clinical oral assessment and clear regression modelling, but limited by cross–sectional temporality and local cohort selection.

		analysis may have introduced selection bias.					
IN–023	Tanaka et al. (2026) [114]	4; Clearly defined community–dwelling cohort aged ≥65 years, with exposure assessed before follow–up trajectories were modelled.	2; Mixed–effects models included a broad set of relevant sociodemographic, behavioural, nutritional, and chronic–disease covariates.	2; Repeated GDS–5 outcome assessment and long follow–up were major strengths, but some oral–frailty components were self–reported and attrition bias remained possible.	8	High	Methodologically strong longitudinal cohort evidence, with minor limitations related mainly to long–term attrition and mixed self–report/clinical exposure assessment.
IN–024	Tay et al. (2025) [115]	3; Clearly defined older cohort (aged ≥60 years), explicit exclusion of baseline depressive cases, and follow–up across three waves.	2; Strong adjustment strategy including time–invariant and time–varying confounders plus marginal structural modelling.	2; Outcome follow–up was adequate and repeated, but chewing disability was self–reported and the outcome was screening–based rather than diagnosis–based.	7	High	Methodologically strong longitudinal evidence, especially because of MSM/IPW, with modest limitations related to self–report, attrition, and screening–based outcome ascertainment.
IN–025	Wang et al. (2026) [116]	4; Clearly defined older–adult population (aged ≥65 years), large national CLHLS sample, and explicit tooth–loss categorization.	2; Fully adjusted depressive model accounted for a broad set of demographic, socioeconomic, behavioural, health, and social–support covariates.	2; Depressive symptoms were measured with CES–D–10, but tooth loss was self–reported and the design was cross–sectional.	8	High	Methodologically strong for a cross–sectional national ageing survey, with main limitations related to temporality and self–reported oral exposure.
IN–026	Wei et al. (2024) [117]	4; Clearly defined older–adult sample (aged >60 years), large national survey framework, transparent flow chart,	2; Fully adjusted model accounted for major demographic, socioeconomic, behavioural, and cardiometabolic	2; Depression was screened with PHQ–9 and oral exposure was derived from dental presence data, but the design was cross–	8	High	Methodologically strong for a cross–sectional national survey, with main limitations related to temporality and screening–based depression ascertainment.

		and standardized dental data allowing FTU derivation.	confounders, with additional checking by propensity–score matching.	sectional and depression was not clinically diagnosed.			
IN–027	Wright et al. (2020) [118]	3; Clearly defined older cohort and standardized year–8 dental assessments, but the analytic sample was reduced from the original CHAMP cohort because of mortality and attrition.	2; Adjusted models accounted for major demographic, socioeconomic, marital, morbidity, and dental–service covariates.	2; Depressive symptoms were measured with GDS–15 and oral variables were collected systematically, but chewing capacity was self–reported and the design was cross–sectional.	7	High	Methodologically solid for a cross–sectional ageing–cohort analysis, with main limitations related to temporality, male–only sampling, and attrition from the parent cohort.
IN–028	Yamamoto et al. (2017) [119]	4; Clearly defined cohort of community–dwelling adults aged ≥65 years and baseline depressive cases were excluded.	2; Fully adjusted model included major socioeconomic, behavioural, health, and baseline mood covariates.	2; Follow–up duration was adequate and outcome measurement used GDS, but oral exposures were self–reported and there was substantial missingness requiring multiple imputation.	8	High	Methodologically strong longitudinal evidence, with modest limitations related to self–reported oral exposure and incomplete follow–up/response data.
IN–029	Zhang et al. (2025) [120]	4; Clearly defined older–adult population (aged ≥60 years), large national CLHLS–based sample, transparent screening flow, and explicit oral–health variables.	2; Models adjusted for key demographic and social covariates relevant to depression and oral health.	2; Depression was measured with CESD–10 and exposures were consistently assessed, but oral indicators were self–reported and there was no longitudinal follow–up.	8	High	Methodologically strong for a cross–sectional national ageing survey, with main limitations related to temporality and self–reported oral measures.
IN–030	Zhang et al. (2021) [121]	3; Clearly defined older cohort (aged ≥65 years), large multicity	2; Fully adjusted model included a broad range of	2; Follow–up duration was adequate and the depressive symptom scale	7	High	Methodologically strong longitudinal evidence, with moderate limitations related mainly to self–report,

	Chinese sample, and explicit follow–up framework, although many baseline participants were lost before final analysis.	demographic, socioeconomic, behavioural, cognitive, functional, and social–engagement covariates.	showed acceptable internal consistency, but oral exposure was self–reported and the outcome instrument was not among the most commonly used geriatric depression scales.		attrition, and outcome–measure indirectness.

Notes: Study–level methodological quality was assessed using the Newcastle–Ottawa Scale (NOS). Cohort studies were evaluated using the standard NOS framework. For analytical cross–sectional studies, a prespecified adapted framework was used that preserved the original three–domain structure and the conventional 0–9 star scale. The Selection domain contributed up to 4 stars and considered study–population representativeness and definition, sampling or recruitment procedures, oral–exposure ascertainment, and relevant response or completeness characteristics. The Comparability domain contributed up to 2 stars according to adjustment for major demographic confounders and additional relevant socioeconomic, behavioural, or health–related covariates. The Outcome/Exposure domain contributed up to 3 stars and considered the validity and consistency of depressive–outcome and oral–exposure measurement; for longitudinal studies, adequacy of follow–up and completeness of outcome assessment were additionally considered. Total NOS scores were interpreted descriptively as high methodological quality (7–9 stars), moderate methodological quality (5–6 stars), or low methodological quality (≤4 stars). Panel A reports the score assigned within each domain together with a study–specific justification, the total NOS score, and the resulting descriptive quality category. These NOS categories represent study–level methodological quality and are not equivalent to GRADE certainty judgments.

Analytical cross–sectional adaptation: explicit scoring rules:

Domain	Score component	Operational rule
**Selection (0–4 stars)**	Target population and sample definition	1 star: source/target population and eligibility were clearly defined and the analytic sample was reasonably representative of the intended older–adult population. 0 stars: population definition was insufficient or representativeness was seriously limited or unclear.
**Selection (0–4 stars)**	Sampling/recruitment	1 star: sampling or recruitment procedures were clearly described and appropriate for the stated population. 0 stars: convenience, highly selected, or insufficiently described recruitment materially limited appraisal of selection.
**Selection (0–4 stars)**	Oral–exposure ascertainment	1 star: the oral exposure was clearly defined and assessed using clinical/objective examination or a standardized, structured, reproducible measure. 0 stars: exposure ascertainment was inadequately defined, ad hoc, or insufficiently described.

**Selection (0–4 stars)**	Participation/analytic completeness	1 star: participation, response, inclusion/exclusion flow, or analytic–sample completeness was adequately reported without major unexplained selection loss. 0 stars: substantial or insufficiently explained non–response, exclusion, or completeness concerns were present.
**Comparability (0–2 stars)**	Core confounding control	1 star: the analysis controlled for major demographic confounding, including age and sex when applicable or appropriate design–specific equivalents. 0 stars: core confounding was not meaningfully controlled or was insufficiently reported.
**Comparability (0–2 stars)**	Additional confounding control	1 additional star: adjustment incorporated additional relevant socioeconomic, behavioural, functional, nutritional, frailty–related, or clinical covariates pertinent to the oral–depression association. 0 additional stars: such adjustment was absent or insufficiently described.
**Outcome/Exposure (0–3 stars)**	Ascertainment validity	3 stars: exposure and depressive outcome were clearly defined and sufficiently robustly ascertained, measurement procedures were applied consistently, and outcome/analytic completeness was adequately reported.
**Outcome/Exposure (0–3 stars)**	One material limitation	2 stars: ascertainment and reporting were generally adequate, but one material limitation or uncertainty affected exposure measurement, depressive–outcome measurement, measurement consistency, or analytic completeness.
**Outcome/Exposure (0–3 stars)**	Multiple material limitations	1 star: multiple important ascertainment or reporting limitations were present, although sufficient information remained for methodological appraisal.
**Outcome/Exposure (0–3 stars)**	Inadequate ascertainment	0 stars: exposure or depressive–outcome ascertainment was inadequately defined or reported to support reliable appraisal.

For analytical cross–sectional studies, the total adapted NOS score was calculated as Selection (0–4) + Comparability (0–2) + Outcome/Exposure (0–3), yielding a maximum of 9 stars. Cross–sectional design itself did not result in an automatic fixed star deduction because absence of follow–up is intrinsic to the design; temporal ambiguity was instead documented in the study–specific interpretation and considered together with measurement and reporting limitations. When reporting was insufficient to verify that a criterion had been met, the more conservative score was assigned. Total scores of 7–9, 5–6, and ≤4 stars were classified descriptively as high, moderate, and low methodological quality, respectively. These operational criteria clarify the scoring framework used for the existing study–level assessments and do not represent a retrospective rescoring of the included studies.

Panel B. Body-level GRADE certainty judgments for the synthesized evidence streams.

Evidence Body/Synthesis Stream	Starting Level of Certainty	Downgrading Factors	Upgrading Factors	Final GRADE Certainty	Rationale Summary
Primary Odds ratio (OR)–only backbone	Low (observational evidence)	Serious inconsistency (substantial heterogeneity), possible publication bias/small–study effects, residual confounding inherent to observational design	None judged sufficient to rate up	Very low	The pooled OR–only stream showed an adverse average association, but certainty was very low because substantial heterogeneity and a prediction interval crossing the null indicated serious inconsistency, while funnel–plot asymmetry and trim–and–fill sensitivity raised concern about possible small–study effects; residual confounding and observational design further limited confidence in the magnitude and transportability of the association.
Secondary RR/PR evidence stream	Low (observational evidence)	Serious indirectness (mixed RR and PR metrics), imprecision (small number of studies), residual confounding	None judged sufficient to rate up	Very low	The direction of association was broadly concordant, but certainty was very low because the evidence stream contained few studies, mixed RR and PR estimands, limited quantitative comparability, indirectness, and residual confounding.
Secondary coefficient–based evidence stream	Low (observational evidence)	Serious inconsistency/indirectness (heterogeneous coefficient types and estimands), imprecision for some studies, residual confounding	None judged sufficient to rate up	Very low	The direction of association was broadly coherent, but the evidence body was highly heterogeneous in metric type, model structure, and interpretability, which substantially limited certainty.
Overall qualitative evidence base	Low (observational evidence)	Serious indirectness (mixed oral constructs and depressive endpoints), residual confounding, limited	Longitudinal support present, but not judged	Very low	The qualitative corpus showed broadly coherent adverse associations across oral domains, but certainty was

	temporality in much of the evidence base	sufficient for formal upgrading	very low because of heterogeneous oral constructs and depressive endpoints, substantial reliance on cross–sectional evidence, residual confounding, limited temporality, and restricted comparability across study designs and measures.

Notes: Panel B reports body–level certainty judgments using the GRADE framework. In accordance with GRADE principles, certainty was assessed at the level of the relevant synthesized evidence body rather than at the level of individual studies. Observational evidence starts at low certainty and may be downgraded for risk of bias, inconsistency, indirectness, imprecision, and publication bias, or upgraded when appropriate. Final ratings were classified as high, moderate, low, or very low. This restructuring aligns Table S5 with the methodological framework stated in Section 2.7 of the manuscript.

Table S6. Frozen master effect-selection dataset for the 30 studies retained in the final review corpus, including analytic-stream assignment, selected effect estimates, stream-eligibility judgments, and planned downstream use.

Record ID	Authors (Year)	Final Corpus Status	Assigned Analytic Stream	Depressive Outcome	Exposure Contrast	Effect Type	Estimate	Lower 95% CI	Upper 95% CI	Adjusted	Eligibility for Assigned Analytic Stream	Planned Downstream Use
IN–001	Hajek et al. (2023) [92]	Quantitative stream retained	Secondary beta/ coefficient stream	Depressive symptoms (Euro–D continuous score)	Presence of missing natural teeth vs absence	Adjusted beta coefficient	0.210	0.171	0.249	Yes	Yes	Retain for structured quantitative narrative synthesis within the secondary beta/coefficient stream; not pooled meta–analytically.
IN–002	Kaur et al. (2024) [93]	Quantitative stream retained	Secondary beta/ coefficient stream	Depression severity (PHQ–9 continuous score)	Missing teeth score (per 1–unit increase)	Adjusted beta coefficient	0.122	0.107	0.129	Yes	Yes, with caution	Retain for structured quantitative narrative synthesis within the secondary beta/coefficient stream; interpret cautiously because comparability with other coefficient–based estimates is limited.
IN–003	Kawamura et al. (2026) [94]	Quantitative stream retained	Primary Odds ratio (OR)–only backbone	Depressive moods (GDS–15 ≥5)	Oral frailty (OF–5 ≥2) vs no oral frailty	Adjusted OR	2.123	1.270	3.550	Yes	Yes	Retain for inverse–variance random–effects meta–analysis within the primary Odds ratio (OR)–only backbone.

IN–004	Koh et al. (2026) [95]	Quantitative stream retained	Primary Odds ratio (OR)–only backbone	Depressive symptoms (binary)	Fewer than 12 remaining natural teeth vs 12 or more, adults aged ≥65 years	Stratified multivariable OR	1.497	1.110	2.020	Yes	Yes	Retain for inverse–variance random–effects meta–analysis within the primary Odds ratio (OR)–only backbone, using the older–adult subgroup estimate only.
IN–005	Kunrath and Silva (2021) [96]	Quantitative stream retained	Secondary Risk ratio/prevalence ratio (RR/PR) stream	Depressive symptoms (GDS–15 ≥5)	Tooth loss between 2009 and 2015 vs same number of teeth	Adjusted PR	1.860	1.010	3.530	Yes	Yes	Retain for structured quantitative narrative synthesis within the secondary Risk ratio/prevalence ratio (RR/PR) stream; not pooled meta–analytically.
IN–006	Kusama et al. (2021) [97]	Quantitative stream retained	Primary Odds ratio (OR)–only backbone	Incident depressive symptoms (GDS–15 ≥5 at follow–up)	≤19 remaining teeth vs ≥20 remaining teeth	Adjusted OR	1.300	1.120	1.510	Yes	Yes	Retain for inverse–variance random–effects meta–analysis within the primary Odds ratio (OR)–only backbone.
IN–007	Lin et al. (2022) [98]	Quantitative stream retained	Primary Odds ratio (OR)–only backbone	Late–life depression (GDS–15 >5)	Oral frailty vs non–oral frailty	Adjusted OR	4.882	2.020	11.800	Yes	Yes	Retain for inverse–variance random–effects meta–analysis within the primary Odds ratio (OR)–only backbone.
IN–008	Lou et al. (2024) [99]	Quantitative stream retained	Secondary beta/coefficient stream	Depressive symptoms score (CESD–10	Denture use vs no denture use	Total effect beta coefficient	–0.341	–0.505	–0.178	Yes	Yes	Retain for structured quantitative narrative synthesis within the secondary

				continuous score)								beta/coefficient stream; preserve total–effect interpretation from the mediation model.
IN–009	Mariño et al. (2020) [100]	Qualitative–only retained	Qualitative–only retention	Depressive symptoms score	Concern about appearance of mouth/ teeth; dentate vs edentulous	Regression coefficient B	Not reported	Not report ed	Not report ed	Yes	No	Retain for qualitative synthesis only; not entered into the frozen quantitative workflow because exact 95% confidence intervals or standard errors were not reported for the selected depressive–symptoms coefficient.
IN–010	Matsuyama et al. (2021) [101]	Quantitative stream retained	Secondary beta/ coefficient stream	Depressive symptoms (PHQ–8 total score, continuous)	Per additional lost tooth, adults aged 60–70 years	2SLS coefficient	–0.055	–1.019	0.909	Yes	Yes, with caution	Retain for structured quantitative narrative synthesis within the secondary beta/coefficient stream; interpret cautiously because the estimate is instrument–based and subgroup–specific.
IN–011	Miyano et al. (2025) [102]	Quantitative stream retained	Primary Odds ratio (OR)–only backbone	Newly diagnosed depression (binary)	Poor chewing ability vs good	Adjusted OR	1.257	1.000	1.580	Yes	Yes	Retain for inverse–variance random–effects meta–analysis within the primary

					chewing ability						Odds ratio (OR)–only backbone.
IN–012	Muniz et al. (2021) [103]	Quantitative stream retained	Primary Odds ratio (OR)–only backbone	Depressive symptoms (GDS–15 >5)	Edentulism (yes vs no)	Adjusted OR	1.296	0.725	2.318	Yes	Yes	Retain for inverse–variance random–effects meta–analysis within the primary Odds ratio (OR)–only backbone; interpret mainly within structural–tooth–loss subgrouping and sensitivity analyses because the adjusted association was null/attenuated.
IN–013	Nakazawa et al. (2024) [104]	Quantitative stream retained	Secondary Risk ratio/prevalence ratio (RR/PR) stream	Incident depressive symptoms (GDS–15 >4 at follow–up)	0–9 remaining teeth without dental prostheses vs ≥20 teeth	Adjusted RR	1.270	1.040	1.560	Yes	Yes	Retain for structured quantitative narrative synthesis within the secondary Risk ratio/prevalence ratio (RR/PR) stream; not pooled meta–analytically.
IN–014	Ohi et al. (2022) [105]	Quantitative stream retained	Primary Odds ratio (OR)–only backbone	Development of depressive symptoms within 4 years (SDS ≥40 at follow–up)	Impaired OHRQoL at baseline vs no impaired OHRQoL	Adjusted OR	6.000	1.380	26.090	Yes	Yes, with caution	Retain for inverse–variance random–effects meta–analysis within the primary Odds ratio (OR)–only backbone; interpret cautiously because the estimate reflects a subjective

											OHRQoL exposure and wide confidence interval.
IN–015	Park et al. (2016) [106]	Quantitative stream retained	Primary Odds ratio (OR)–only backbone	Depressive symptoms (CES–D ≥16)	Chewing difficulty vs no chewing difficulty	Derived sex–combined adjusted OR	1.486	1.396	1.581	Yes	Yes	Retain for inverse–variance random–effects meta–analysis within the primary Odds ratio (OR)–only backbone.
IN–016	Park et al. (2023) [107]	Quantitative stream retained	Primary Odds ratio (OR)–only backbone	Depressive symptoms in 2020 (CES–D10 ≥4)	Worse oral physical function score (inverse–coded from the reported per 1–point increase, 2018–2020)	Adjusted OR	1.113	1.053	1.177	Yes	Yes, with caution	Retain for inverse–variance random–effects meta–analysis within the primary Odds ratio (OR)–only backbone; effect direction was inverse–coded for harmonized interpretation.
IN–017	Rouxel et al. (2018) [108]	Quantitative stream retained	Primary Odds ratio (OR)–only backbone	Incident depressive symptoms (CES–D, dichotomiz ed)	Incident oral impact vs no change in oral impact	Adjusted OR	1.606	1.200	2.150	Yes	Yes, with caution	Retain for inverse–variance random–effects meta–analysis within the primary Odds ratio (OR)–only backbone; interpret cautiously because the exposure reflects change in oral impacts rather than a structural oral indicator.

IN–018	Skośkiewicz–Malinowska et al. (2018) [109]	Quantitative stream retained	Secondary beta/ coefficient stream	Depression severity (PHQ–9 total score)	Number of extracted teeth (per additional tooth)	Adjusted beta coefficient	0.111	0.105	0.116	Yes	Yes, with caution	Retain for structured quantitative narrative synthesis within the secondary beta/coefficient stream; interpret cautiously because the estimate is not directly comparable with all other coefficient types.
IN–019	Sun et al. (2022) [110]	Quantitative stream retained	Secondary beta/ coefficient stream	Depressive symptoms score (CES–D–10 continuous score)	Self–rated oral health (per 1–category increase)	Total effect beta coefficient	–0.220	–0.440	–0.010	Yes	Yes	Retain for structured quantitative narrative synthesis within the secondary beta/coefficient stream; preserve total–effect interpretation from the reported mediation framework.
IN–020	Sun et al. (2025) [111]	Quantitative stream retained	Primary Odds ratio (OR)–only backbone	Depressive symptoms (PHQ–9, binary as modeled in logistic regression)	Oral frailty vs no oral frailty	Adjusted OR	3.136	2.430	4.046	Yes	Yes	Retain for inverse–variance random–effects meta–analysis within the primary Odds ratio (OR)–only backbone.
IN–021	Sung et al. (2025) [112]	Quantitative stream retained	Secondary beta/ coefficient stream	Depressive symptoms in 2020 (CES–D–10	Oral health in 2018 (GOHAI score;	Standardized beta coefficient	–0.040	–0.060	–0.030	Yes	Yes, with caution	Retain for structured quantitative narrative synthesis within the secondary

				continuous score)	higher = better oral health)							beta/coefficient stream; interpret cautiously because the estimate is standardized and derives from a panel model.
IN–022	Takiguchi et al. (2016) [113]	Quantitative stream retained	Primary Odds ratio (OR)–only backbone	High depressive–symptom burden (GHQ–30 ≥7)	Low unstimulated salivary flow rate (<0.2 mL/min) vs normal rate	Adjusted OR	2.163	1.200	3.900	Yes	Yes, with caution	Retain for inverse–variance random–effects meta–analysis within the primary Odds ratio (OR)–only backbone; interpret cautiously because the exposure represents oral dryness/salivary dysfunction rather than a dentition–based construct.
IN–023	Tanaka et al. (2026) [114]	Quantitative stream retained	Secondary beta/ coefficient stream	Trajectory of depressive symptoms (GDS–5 score)	Baseline oral frailty vs non–oral frailty	Adjusted beta coefficient	0.126	0.062	0.189	Yes	Yes, with caution	Retain for structured quantitative narrative synthesis within the secondary beta/coefficient stream; interpret cautiously because the estimate derives from a longitudinal mixed–effects model.
IN–024	Tay et al. (2025) [115]	Quantitative stream retained	Secondary Risk ratio/preval	Clinically significant depressive	Chewing disability vs no	Adjusted RR	1.480	1.150	1.820	Yes	Yes	Retain for structured quantitative narrative synthesis

			ence ratio (RR/PR) stream	symptoms over 6 years	chewing disability							within the secondary Risk ratio/prevalence ratio (RR/PR) stream; not pooled meta–analytically.
IN–025	Wang et al. (2026) [116]	Quantitative stream retained	Primary Odds ratio (OR)–only backbone	Depressive symptoms (CES–D–10 ≥10)	20–27 tooth loss vs 0–8 tooth loss	Adjusted OR	1.337	1.106	1.617	Yes	Yes	Retain for inverse–variance random–effects meta–analysis within the primary Odds ratio (OR)–only backbone.
IN–026	Wei et al. (2024) [117]	Quantitative stream retained	Primary Odds ratio (OR)–only backbone	Depression (PHQ–9 ≥10)	0–3 functional tooth units (FTUs) vs 10–12 functional tooth units (FTUs)	Adjusted OR	1.818	1.157	2.858	Yes	Yes	Retain for inverse–variance random–effects meta–analysis within the primary Odds ratio (OR)–only backbone.
IN–027	Wright et al. (2020) [118]	Quantitative stream retained	Secondary Risk ratio/prevalence ratio (RR/PR) stream	Depressive symptoms (GDS–15 ≥5)	Limited chewing capacity vs no chewing problems	Adjusted PR	1.930	1.340	2.790	Yes	Yes	Retain for structured quantitative narrative synthesis within the secondary Risk ratio/prevalence ratio (RR/PR) stream; not pooled meta–analytically.
IN–028	Yamamoto et al. (2017) [119]	Quantitative stream retained	Primary Odds ratio (OR)–only backbone	Incident depressive symptoms (GDS ≥5 at follow–up)	No teeth vs ≥20 teeth	Adjusted OR	1.284	1.030	1.600	Yes	Yes	Retain for inverse–variance random–effects meta–analysis within the primary Odds ratio (OR)–only backbone.

IN–029	Zhang et al. (2025) [120]	Quantitative stream retained	Secondary beta/ coefficient stream	Depression level (CESD–10 continuous score)	Remaining teeth (per unit increase)	Total effect beta coefficient	–0.018	–0.031	–0.005	Yes	Yes	Retain for structured quantitative narrative synthesis within the secondary beta/coefficient stream; preserve total–effect interpretation from the mediation model.
IN–030	Zhang et al. (2021) [121] †	Quantitative stream retained	Secondary beta/ coefficient stream	Depressive symptom score at 4–year follow–up	1–9 natural teeth vs ≥20 natural teeth	Adjusted beta coefficient	0.590	0.300	0.650	Yes	Yes	Retain for structured quantitative narrative synthesis within the secondary beta/coefficient stream; not pooled meta–analytically.

Notes: This table represents the frozen master effect–selection dataset for all 30 studies retained in the final review corpus. Each record was assigned to one prespecified downstream evidence stream according to metric comparability, outcome format, and inferential role. "Assigned Analytic Stream" identifies whether the selected estimate was retained for the primary Odds ratio (OR)–only backbone, the secondary Risk ratio/prevalence ratio (RR/PR) stream, the secondary beta/coefficient stream, or qualitative–only retention. "Eligibility for Assigned Analytic Stream" indicates whether the selected estimate was judged sufficiently comparable for inclusion in its designated downstream synthesis framework; this should not be interpreted as pooled–meta–analysis eligibility in all cases. "Planned Downstream Use" reflects the final analytical workflow of the review: only the Odds ratio (OR)–only backbone entered the primary pooled meta–analysis, whereas RR/PR and beta/coefficient studies were retained for structured quantitative narrative synthesis, and one study was retained for qualitative synthesis only. Directionality was harmonized at the interpretive level so that retained estimates consistently reflected greater oral impairment in relation to greater depressive burden. Standardized mathematical notation has been used throughout, for example, aged ≥65 years, ≤19 remaining teeth, and 0–3 functional tooth units (FTUs) vs 10–12 functional tooth units (FTUs).

† Zhang et al. (2021) [121] reported the coefficient for the 1–9 teeth category as 0.59 with a 95% CI of 0.30–0.65. Because the published confidence interval is not centered on the reported point estimate, the value was retained exactly as reported in the source study and interpreted narratively only; it was not used in pooled coefficient meta–analysis.

Table S7. Reconciliation of derived analytic streams with the frozen master effect-selection dataset (Table S6), including quantitative-stream coverage and qualitative-only retention.

Stream	Records in Stream (n)	Records Found in Frozen Master Dataset (n)	Records Missing from Frozen Master Dataset (n)
Primary Odds ratio (OR)–only backbone	15	15	0
Secondary Risk ratio/prevalence ratio (RR/PR) stream	4	4	0
Secondary beta/coefficient stream	10	10	0
Union of all quantitative streams	29	29	0
Qualitative–only retention	1	1	0
Total studies retained in the final review corpus	30	30	0

Notes: This reconciliation table documents the internal consistency between the frozen master effect–selection dataset (Table S6) and the derived analytic streams retained for the final evidence–synthesis workflow. The primary Odds ratio (OR)–only backbone, the secondary Risk ratio/prevalence ratio (RR/PR) stream, and the secondary beta/coefficient stream together accounted for 29 of the 30 studies retained in the final review corpus, with no missing records relative to the frozen master dataset. One study, IN–009 Mariño et al. (2020) [100], was retained in the final review corpus but contributed only to qualitative synthesis and therefore did not enter any quantitative analytic stream. Consequently, the union of all quantitative streams comprised 29 studies, whereas the total frozen review corpus remained 30 studies. The primary Odds ratio (OR)–only backbone supported the pooled meta–analysis, whereas the RR/PR and beta/coefficient streams were retained for structured quantitative narrative synthesis outside the primary pooled framework.

Table S8. Primary Odds ratio (OR)-only backbone dataset used in the final quantitative workflow.

Study (ID, Authors, Year)	Region (Country)	Design Class	Ascertainment Type	Older–Adult Frame	Exposure Family	Depressive Outcome	Exposure Contrast	Effect Metric and Estimate (95% CI)	Log Effect	SE Log Effect	Var Log Effect	NOS Total Score	NOS Methodological Quality Category
IN–003/ Kawamura et al. (2026) [94]	Asia (Japan)	Cross–sectional	Likely self–report or mixed	Strict older–adult cohort/ subsample	Oral frailty/ functional decline	Depressive moods (GDS–15 ≥5)	Oral frailty (OF–5 ≥2) vs no oral frailty	OR 2.123 (1.270–3.550)	0.7530	0.2622	0.0687	5	Moderate
IN–004/ Koh et al. (2026) [95]	Asia (South Korea)	Cross–sectional	Likely clinical/ objective or structured	Older–adult subgroup within broader–age sample	Tooth loss/ dentition	Depressive symptoms (binary)	Fewer than 12 remaining natural teeth vs 12 or more, adults aged ≥65 years	OR 1.497 (1.110–2.020)	0.4037	0.1527	0.0233	7	High
IN–006/ Kusama et al. (2021) [97]	Asia (Japan)	Longitudinal	Likely clinical/ objective or structured	Strict older–adult cohort/ subsample	Tooth loss/ dentition	Incident depressive symptoms (GDS–15 ≥5 at follow–up)	≤19 remaining teeth vs ≥20 remaining teeth	OR 1.300 (1.120–1.510)	0.2627	0.0762	0.0058	9	High

IN–007/ Lin et al. (2022) [98]	Asia (Taiwan)	Cross–sectional	Likely self–report or mixed	Strict older–adult cohort/ subsample	Oral frailty/ functional decline	Late–life depression (GDS–15 >5)	Oral frailty vs non–oral frailty	OR 4.882 (2.020–11.800)	1.5856	0.4503	0.2027	8	High
IN–011/ Miyano et al. (2025) [102]	Asia (Japan)	Longitudinal	Unclear	Strict older–adult cohort/ subsample	Chewing dysfunction	Newly diagnosed depression (binary)	Poor chewing ability vs good chewing ability	OR 1.257 (1.000–1.580)	0.2287	0.1167	0.0136	8	High
IN–012/ Muniz et al. (2021) [103]	South America (Brazil)	Cross–sectional	Unclear	Strict older–adult cohort/ subsample	Tooth loss/ dentition	Depressive symptoms (GDS–15 >5)	Edentulism (yes vs no)	OR 1.296 (0.725–2.318)	0.2593	0.2965	0.0879	6	Moderate_or_lower
IN–014/ Ohi et al. (2022) [105]	Asia (Japan)	Longitudinal	Likely clinical/ objective or structured	Strict older–adult cohort/ subsample	OHRQoL/ oral impacts	Development of depressive symptoms within 4 years (SDS ≥40 at follow–up)	Impaired OHRQoL at baseline vs no impaired OHRQoL	OR 6.000 (1.380–26.090)	1.7918	0.7499	0.5623	7	High
IN–015/ Park et al. (2016) [106]	Asia (South Korea)	Cross–sectional	Likely self–report or mixed	Strict older–adult cohort/ subsample	Chewing dysfunction	Depressive symptoms (CES–D ≥16)	Chewing difficulty vs no chewing difficulty	OR 1.486 (1.396–1.581)	0.3961	0.0317	0.0010	8	High

IN–016/ Park et al. (2023) [107]	Asia (South Korea)	Longitudinal	Likely self–report or mixed	Strict older–adult cohort/ subsample	Other oral impairment	Depressive symptoms in 2020 (CES–D10 ≥4)	Worse oral physical function score (inverse–coded from the reported per 1–point increase, 2018–2020)	OR 1.113 (1.053–1.177)	0.1071	0.0284	0.0008	7	High
IN–017/ Rouxel et al. (2018) [108]	Europe (United Kingdom)	Longitudinal	Unclear	Strict older–adult cohort/ subsample	OHRQoL/ oral impacts	Incident depressive symptoms (CES–D, dichotomized)	Incident oral impact vs no change in oral impact	OR 1.606 (1.200–2.150)	0.4737	0.1488	0.0221	7	High
IN–020/ Sun et al. (2025) [111]	Asia (China)	Cross–sectional	Likely self–report or mixed	Strict older–adult cohort/ subsample	Oral frailty/ functional decline	Depressive symptoms (PHQ–9, binary as modeled)	Oral frailty vs no oral frailty	OR 3.136 (2.430–4.046)	1.1429	0.1301	0.0169	7	High
IN–022/ Takiguchi et al. (2016) [113]	Asia (Japan)	Cross–sectional	Unclear	Strict older–adult cohort/ subsample	Xerostomia/ salivary dysfunction	High depressive–symptom burden (GHQ–30 ≥7)	Low unstimulated salivary flow rate (<0.2 mL/min)	OR 2.163 (1.200–3.900)	0.7716	0.3007	0.0904	7	High

							vs normal rate						
IN–025/ Wang et al. (2026) [116]	Asia (China)	Cross–sectional	Unclear	Strict older–adult cohort/ subsample	Tooth loss/ dentition	Depressive symptoms (CES–D–10 ≥10)	20–27 tooth loss vs 0–8 tooth loss	OR 1.337 (1.106–1.617)	0.2907	0.0969	0.0094	8	High
IN–026/ Wei et al. (2024) [117]	North America (USA)	Cross–sectional	Unclear	Strict older–adult cohort/ subsample	Other oral impairment	Depression (PHQ–9 ≥10)	0–3 functional tooth units (FTUs) vs 10–12 functional tooth units (FTUs)	OR 1.818 (1.157–2.858)	0.5980	0.2307	0.0532	8	High
IN–028/ Yamamoto et al. (2017) [119]	Asia (Japan)	Longitudinal	Unclear	Strict older–adult cohort/ subsample	Tooth loss/ dentition	Incident depressive symptoms (GDS ≥5 at follow–up)	No teeth vs ≥20 teeth	OR 1.284 (1.030–1.600)	0.2498	0.1124	0.0126	8	High

Notes: This table reports the finalized study–level dataset used in the primary OR–only quantitative workflow. All retained records contributed to the primary inverse–variance random–effects meta–analysis of binary depressive outcomes. “Older–Adult Frame” indicates whether the selected estimate was derived from a strictly older–adult cohort/subsample or from an eligible older–adult subgroup nested within a broader–

age sample. "Ascertainment Type" was classified as likely clinical/objective or structured, likely self–report or mixed, or unclear on the basis of the retained exposure–outcome estimate. "Estimate (95% CI)" reports the selected study–specific odds ratio and corresponding 95% confidence interval after directionality harmonization, such that higher values consistently reflect greater oral impairment in relation to greater depressive burden. "Log Effect", "SE Log Effect", and "Var Log Effect" denote the logarithmic transformation of the retained odds ratio, the corresponding standard error, and the within–study variance used in inverse–variance meta–analysis. NOS methodological quality categories are shown in the operational form used for downstream subgrouping and moderator analyses, where scores of 7–9 were classified as High and scores of 5–6 as Moderate. This study–level quality grouping is not equivalent to the body–level GRADE certainty framework reported separately in Table S5.

Table S9. Secondary RR/PR dataset retained for structured quantitative narrative synthesis.

Study (ID, Author, Year)	Region (Country)	Design Class	Ascertainment Type	Older–Adult Frame	Exposure Family	Depressive Outcome	Exposure Contrast	Effect Metric and Estimate (95% CI)	Log Effect	SE Log Effect	Var Log Effect	NOS Total Score	NOS Methodological Quality Category
IN–005/ Kunrath and Silva (2021) [96]	South America (Brazil)	Longitudinal	Unclear	Strict older–adult cohort/ subsample	Tooth loss/ dentition	Depressive moods (GDS–15 ≥5)	Tooth loss between 2009 and 2015 vs same number of teeth	PR 1.860 (1.010–3.530)	0.6206	0.3192	0.1019	7	High
IN–013/ Nakazawa et al. (2024) [104]	Asia (Japan)	Longitudinal	Likely clinical/ objective or structured	Strict older–adult cohort/ subsample	Denture/ prosthetic status	Incident depressive symptoms (GDS–15 >4 at follow–up)	0–9 remaining teeth without dental prostheses vs ≥20 teeth	RR 1.270 (1.040–1.560)	0.2390	0.1034	0.0107	8	High

IN–024/ Tay et al. (2025) [115]	Asia (Singapore)	Longitudinal	Likely self–report or mixed	Strict older–adult cohort/ subsample	Chewing dysfunction	Clinically significant depressive symptoms over 6 years	Chewing disability vs no chewing disability	RR 1.480 (1.150–1.820)	0.3920	0.1171	0.0137	7	High
IN–027/ Wright et al. (2020) [118]	Oceania (Australia)	Cross–sectional	Unclear	Strict older–adult cohort/ subsample	Chewing dysfunction	Depressive symptoms (GDS–15 ≥5)	Limited chewing capacity vs no chewing problems	PR 1.930 (1.340–2.790)	0.6575	0.1871	0.0350	7	High

Notes: This table summarizes the secondary RR/PR evidence stream retained for structured quantitative narrative synthesis rather than pooled meta–analysis. The included studies reported risk ratios (RRs) or prevalence ratios (PRs), which were preserved on their original ratio scale and interpreted in a directionally harmonized manner so that higher values consistently reflected greater oral impairment in relation to greater depressive burden. "Estimate (95% CI)" reports the selected study–specific point estimate and corresponding 95% confidence interval. "Log Effect", "SE Log Effect", and "Var Log Effect" are provided to preserve traceability within the frozen analytical workflow, but these quantities were not used to generate a pooled secondary meta–analysis because the stream combined prevalence–based and risk–based measures and remained too small and conceptually mixed for robust formal pooling. NOS methodological quality category is a study–level classification derived from the total Newcastle–Ottawa Scale score and is not equivalent to the GRADE certainty–of–evidence judgment reported separately in Table S5.

Table S10. Secondary beta/coefficient dataset retained for structured quantitative narrative synthesis.

Study (ID, Authors, Year)	Region (Country)	Design Class	Ascertainment Type	Older–Adult Frame	Exposure Family	Depressive Outcome	Exposure Contrast	Effect Metric and Estimate (95% CI)	SE Coefficient	Var Coefficient	NOS Total Score	NOS Methodological Quality Category
IN–001/ Hajek et al. (2023) [92]	Europe/ Middle East (Multinational/ Israel)	Cross–sectional	Likely clinical/ objective or structured	Broader–age ageing cohort with eligible later–life analytic frame	Tooth loss/ dentition	Depressive symptoms (Euro–D continuous score)	Presence of missing natural teeth vs absence	Adjusted beta coefficient 0.210 (0.171; 0.249)	0.0199	0.00039601	8	High
IN–002/ Kaur et al. (2024) [93]	Asia (India)	Cross–sectional	Unclear	Strict older–adult cohort/subsample	Tooth loss/ dentition	Depression severity (PHQ–9 continuous score)	Missing teeth score (per 1–unit increase)	Adjusted beta coefficient 0.122 (0.107; 0.129)	0.0056	0.00003136	6	Moderate
IN–008/ Lou et al. (2024) [99]	Asia (China)	Cross–sectional	Likely clinical/ objective or structured	Strict older–adult cohort/subsample	Denture/ prosthetic status	Depressive symptoms score (CESD–10 continuous score)	Denture use vs no denture use	Total effect beta coefficient −0.341 (−0.505; −0.178)	0.0834	0.00695863	9	High
IN–010/ Matsuyama et al.	North America (USA)	Cross–sectional	Unclear	Older–adult subgroup within	Tooth loss/ dentition	Depressive symptoms (PHQ–8 total	Per additional lost tooth, adults	2SLS coefficient −0.055	0.4918	0.24186724	7	High

(2021) [101]				broader–age sample		score, continuous)	aged 60–70 years	(−1.019; 0.909)				
IN–018/ Skośkiewicz–Malinowska et al. (2018) [109]	Europe (Poland)	Cross–sectional	Unclear	Strict older–adult cohort/subsample	Tooth loss/ dentition	Depression severity (PHQ–9 total score)	Number of extracted teeth (per additional tooth)	Adjusted beta coefficient 0.111 (0.105; 0.116)	0.0028	0.00000784	6	Moderate
IN–019/ Sun et al. (2022) [110]	Asia (China)	Cross–sectional	Unclear	Strict older–adult cohort/subsample	Other oral impairment	Depressive symptoms score (CES–D–10 continuous score)	Self–rated oral health (per 1–category increase)	Total effect beta coefficient −0.220 (−0.440; −0.010)	0.1097	0.01203409	8	High
IN–021/ Sung et al. (2025) [112]	Asia (South Korea)	Longitudinal	Unclear	Strict older–adult cohort/subsample	Other oral impairment	Depressive symptoms in 2020 (CES–D–10 continuous score)	Oral health in 2018 (GOHAI score; higher = better oral health)	Standardized beta coefficient −0.040 (−0.060; −0.030)	0.0077	0.00005929	7	High
IN–023/ Tanaka et al. (2026) [114]	Asia (Japan)	Longitudinal	Likely self–report or mixed	Strict older–adult cohort/subsample	Oral frailty/ functional decline	Trajectory of depressive symptoms (GDS–5 score)	Baseline oral frailty vs non–oral frailty	Adjusted beta coefficient 0.126 (0.062; 0.189)	0.0324	0.00104976	8	High
IN–029/ Zhang et al.	Asia (China)	Cross–sectional	Likely clinical/ objective or structured	Strict older–adult	Tooth loss/ dentition	Depression level (CESD–10	Remaining teeth	Total effect beta coefficient	0.0066	0.00004356	8	High

(2025) [120]				cohort/subsample		continuous score)	(per unit increase)	−0.018 (−0.031; −0.005)				
IN–030/ Zhang et al. (2021) [121] †	Asia (China)	Longitudinal	Likely clinical/ objective or structured	Strict older–adult cohort/subsample	Tooth loss/ dentition	Depressive symptom score at 4–year follow–up	1–9 natural teeth vs ≥20 natural teeth	Adjusted beta coefficient 0.590 (0.300; 0.650)	0.0893	0.00797449	7	High

Notes: This table summarizes the secondary continuous–effect evidence stream retained for structured quantitative narrative synthesis rather than pooled meta–analysis. The included studies reported heterogeneous coefficient types, including adjusted beta coefficients, standardized beta coefficients, total–effect beta coefficients, and two–stage least squares (2SLS) coefficients, which differ in scale, interpretation, and underlying modeling assumptions. Because these estimates were not directly interchangeable and did not support a sufficiently comparable common metric for robust pooled synthesis, they were not meta–analyzed as a single coefficient stream. “Estimate (95% CI)” reports the selected study–specific continuous–effect estimate together with its 95% confidence interval. “SE Coefficient” denotes the standard error of the selected coefficient estimate, and “Var Coefficient” denotes the corresponding within–study variance, calculated as SE^2. These quantities are retained to preserve traceability within the frozen analytical workflow and should not be interpreted as implying formal pooled meta–analysis across the full beta/coefficient stream. The NOS methodological quality category is a study–level classification derived from the total Newcastle–Ottawa Scale score and is not equivalent to the GRADE certainty–of–evidence judgment reported separately in Table S5. Directional interpretation should consider the original coding of each exposure and outcome, including whether higher values reflect poorer or better oral status or function. Adjusted beta coefficients represent regression coefficients estimated from multivariable models that account for measured covariates included by the original study investigators. Standardized beta coefficients express associations in standard deviation units and may facilitate comparison across differently scaled variables, but they are not directly interchangeable with unstandardized coefficients. Total–effect beta coefficients refer to coefficients intended to quantify the overall association between exposure and outcome without decomposition into direct and indirect pathways; depending on the analytic framework, such estimates may arise from mediation–oriented or structural models and should therefore be interpreted in relation to the original modeling assumptions. 2SLS coefficients are derived from two–stage least squares instrumental–variable models and are not equivalent to conventional multivariable adjusted beta coefficients; instead, they estimate the exposure–outcome association using instrument–based variation under additional identifying assumptions, including instrument relevance and exclusion restriction. Because these coefficient types differ in scale, interpretation, and statistical assumptions, comparability across studies was considered limited and pooling was not undertaken across the full beta/coefficient stream.

† Zhang et al. (2021) [121] reported the coefficient for the 1–9 teeth category as 0.59 with a 95% CI of 0.30–0.65. Because the published confidence interval is not centered on the reported point estimate, the value was retained exactly as reported in the source study and interpreted narratively only; it was not used in pooled coefficient meta–analysis.

Table S11. Leave-one-out diagnostics for the primary OR-only random-effects meta-analysis.

Excluded Study	Excluded Record ID	Studies Retained (k)	Pooled OR	Lower 95% CI	Upper 95% CI	HKSJ t Statistic	HKSJ p–Value	Cochran's Q	Q–Test p–Value	I^2 (%)	τ^2 (REML)	Lower 95% Prediction Limit	Upper 95% Prediction Limit
Kawamura et al. (2026) [94]	IN–003	14	1.590	1.276	1.981	4.557	<0.001	114.037	<0.001	88.600	0.083	0.873	2.895
Koh et al. (2026) [95]	IN–004	14	1.633	1.300	2.052	4.639	<0.001	116.657	<0.001	88.856	0.093	0.867	3.075
Kusama et al. (2021) [97]	IN–006	14	1.655	1.321	2.075	4.822	<0.001	117.355	<0.001	88.923	0.090	0.887	3.087
Lin et al. (2022) [98]	IN–007	14	1.554	1.290	1.873	5.114	<0.001	108.892	<0.001	88.062	0.068	0.908	2.660
Miyano et al. (2025) [102]	IN–011	14	1.656	1.324	2.071	4.872	<0.001	117.222	<0.001	88.910	0.089	0.893	3.070
Muniz et al. (2021) [103]	IN–012	14	1.637	1.311	2.044	4.789	<0.001	117.379	<0.001	88.925	0.088	0.884	3.031
Ohi et al. (2022) [105]	IN–014	14	1.584	1.298	1.933	4.989	<0.001	113.286	<0.001	88.525	0.077	0.893	2.811
Park et al. (2016) [106]	IN–015	14	1.639	1.301	2.065	4.623	<0.001	95.077	<0.001	86.327	0.095	0.864	3.110
Park et al. (2023) [107]	IN–016	14	1.671	1.350	2.069	5.199	<0.001	55.993	<0.001	76.783	0.075	0.944	2.960
Rouxel et al. (2018) [108]	IN–017	14	1.624	1.292	2.042	4.575	<0.001	115.561	<0.001	88.751	0.093	0.863	3.057

Sun et al. (2025) [111]	IN–020	14	1.421	1.231	1.640	5.280	<0.001	71.928	<0.001	81.926	0.018	1.057	1.910
Takiguchi et al. (2016) [113]	IN–022	14	1.592	1.278	1.982	4.583	<0.001	114.641	<0.001	88.660	0.083	0.875	2.895
Wang et al. (2026) [116]	IN–025	14	1.651	1.316	2.071	4.774	<0.001	117.353	<0.001	88.922	0.092	0.881	3.092
Wei et al. (2024) [117]	IN–026	14	1.607	1.283	2.013	4.556	<0.001	115.405	<0.001	88.735	0.088	0.867	2.979
Yamamoto et al. (2017) [119]	IN–028	14	1.654	1.321	2.071	4.837	<0.001	117.331	<0.001	88.920	0.090	0.889	3.078

Notes: Leave–one–out diagnostics were calculated by iteratively excluding one study at a time from the primary OR–only random–effects meta–analysis and refitting the model to the remaining k = 14 studies. All calculations were derived exclusively from the primary Odds ratio (OR)–only backbone data reported in Table S8. Pooled odds ratios (ORs) and 95% confidence intervals (CIs) were estimated on the logarithmic scale and back–transformed for presentation. Between–study variance was estimated using restricted maximum likelihood (REML), and inference for the refitted pooled effect was based on the Hartung–Knapp–Sidik–Jonkman (HKSJ) adjustment. Cochran's Q denotes the heterogeneity statistic for the refitted model after exclusion of the indicated study. I^2 (%) represents the proportion of total variability attributable to between–study heterogeneity. The lower and upper prediction limits are reported on the OR scale and correspond to the 95% prediction interval for a new comparable study after exclusion of the specified record. P values below 0.001 are reported as <0.001.

Table S12. Exploratory subgroup random-effects analyses within the primary Odds ratio (OR)-only backbone.

Grouping Variable	Group Level	k	Pooled OR	Lower 95% CI	Upper 95% CI	HKSJ t Statistic	HKSJ p–Value	Cochran's Q	Q–Test p–Value	I^2 (%)	τ^2 (REML)	Lower 95% Prediction Limit	Upper 95% Prediction Limit
Design Class	Cross–sectional	9	1.862	1.385	2.502	4.849	0.001	43.488	<0.001	81.604	0.094	0.969	3.575
Design Class	Longitudinal	6	1.266	1.047	1.531	3.190	0.024	15.260	0.009	67.235	0.010	0.994	1.613
Exposure Family	Chewing dysfunction	2	1.419	0.550	3.659	4.695	0.134	1.909	0.167	47.627	0.007	1.143	1.762
Exposure Family	OHRQoL/ oral impacts	2	2.529	0.001	7,225.535	1.482	0.378	2.972	0.085	66.348	0.576	0.368	17.404
Exposure Family	Oral frailty/ functional decline	3	2.967	1.459	6.037	6.590	0.022	3.022	0.221	33.827	0.016	1.974	4.460
Exposure Family	Other oral impairment	2	1.349	0.064	28.347	1.248	0.430	4.464	0.035	77.598	0.094	0.630	2.889
Exposure Family	Tooth loss/dentition	5	1.326	1.247	1.409	12.842	<0.001	0.795	0.939	0.000	0.000	1.270	1.384
Region	Asia	12	1.651	1.257	2.168	4.046	0.002	113.545	<0.001	90.312	0.113	0.819	3.329
Region	Non–Asia	3	1.604	1.170	2.200	6.437	0.023	0.812	0.666	0.000	0.000	1.389	1.852
Ascertainment Type	Likely clinical/ objective or structured	3	1.335	1.129	1.580	7.394	0.018	0.693	0.707	0.000	0.000	1.237	1.442
Ascertainment Type	Likely self–report or mixed	5	2.588	1.350	4.961	4.058	0.015	42.177	<0.001	90.516	0.192	0.978	6.849
Ascertainment Type	Unclear	7	1.336	1.122	1.592	4.051	0.007	18.420	0.005	67.427	0.017	0.998	1.789
NOS Methodological Quality Category	High	13	1.614	1.273	2.046	4.395	<0.001	114.036	<0.001	89.477	0.091	0.862	3.023
NOS Methodological Quality Category	Moderate	2	1.692	0.074	38.498	2.138	0.279	1.554	0.213	35.648	0.043	0.899	3.182

Older–Adult Frame	Strict older–adult cohort/subsample	14	1.633	1.300	2.052	4.639	<0.001	116.657	<0.001	88.856	0.093	0.867	3.075

Notes: Exploratory subgroup analyses were conducted within the primary OR–only random–effects meta–analytic backbone using inverse–variance weighting, restricted maximum likelihood (REML) estimation of between–study variance, and Hartung–Knapp–Sidik–Jonkman (HKSJ) adjustment for inference. Pooled odds ratios (ORs) and 95% confidence intervals (CIs) were estimated on the logarithmic scale and back–transformed for presentation. Cochran's Q denotes within–subgroup heterogeneity, and I^2 (%) represents the proportion of total variability attributable to between–study heterogeneity within each subgroup. The lower and upper prediction limits are reported on the OR scale and correspond to the 95% prediction interval for a new comparable study within the respective subgroup. These subgroup analyses were exploratory and should be interpreted cautiously, particularly for strata with small numbers of studies. No formal between–subgroup interaction test is presented in this table; therefore, differences in pooled estimates across subgroup levels should not be interpreted as definitive evidence of effect modification. P values below 0.001 are reported as <0.001.

Table S13. Exploratory univariate mixed-effects meta-regression summary for the primary Odds ratio (OR)-only backbone.

Moderator	Moderator Type	Reference Category	Levels Included	Studies Included (k)	Residual τ^2 (REML)	Residual Heterogeneity (QE)	QE df	Moderator Test (QM)	QM df	QM p–Value
Design Class	Categorical	Cross–sectional	Cross–sectional; Longitudinal	15	0.0552	16.5216	13	3.4177	1	0.0645
Exposure Family	Categorical	Chewing dysfunction	Chewing dysfunction; OHRQoL/oral impacts; Oral frailty/functional decline; Other oral impairment; Tooth loss/dentition	14	0.0003	12.9485	9	60.8724	4	<0.0001
Region	Categorical	Asia	Asia; Non–Asia	15	0.0916	15.6526	13	0.0134	1	0.9079
Ascertainment Type	Categorical	Likely clinical/ objective or structured	Likely clinical/objective or structured; Likely self–report or mixed; Unclear	15	0.0542	12.0056	12	9.0584	2	0.0108
NOS Methodological Quality Category	Categorical	High	High; Moderate	15	0.0878	16.0419	13	0.0198	1	0.8882
NOS Total Score	Continuous	Not applicable	Not applicable	15	0.0771	16.2069	13	0.8834	1	0.3473

Notes: Meta–regression analyses were conducted on the primary OR–only random–effects meta–analytic backbone and were prespecified as exploratory assessments of between–study heterogeneity. Each model included only one moderator at a time to preserve parsimony given the limited number of studies. Categorical moderators were parameterized relative to the stated reference category. QM denotes the omnibus test of the moderator, whereas QE denotes residual heterogeneity after accounting for the moderator. Between–study variance was estimated using restricted maximum likelihood (REML). Because the number of studies was modest and some moderator levels were sparsely populated, these results should be interpreted cautiously and should not be regarded as definitive evidence of effect modification. For continuous moderators, a reference category is not applicable. Values smaller than 0.0001 are reported as <0.0001. Statistically significant QM results should be interpreted as exploratory heterogeneity signals rather than as evidence of true moderator–driven effect modification.

Table S14. Coefficient-level results from exploratory univariate mixed-effects meta-regression models for the primary Odds ratio (OR)-only backbone.

Coefficient Term	Estimate (Log Scale)	HKSJ SE	HKSJ t Statistic	HKSJ p–Value	Lower 95% CI (Log Scale)	Upper 95% CI (Log Scale)	Moderator	Moderator Type	Reference Category	Back–Transformed Estimate (Ratio Scale)	Lower 95% CI (Ratio Scale)	Upper 95% CI (Ratio Scale)
Intercept	0.6022	0.1157	5.2045	0.0002	0.3522	0.8522	Design Class	Categorical	Cross–sectional	1.8261	1.4222	2.3448
Longitudinal	–0.3184	0.1722	–1.8487	0.0874	–0.6905	0.0537	Design Class	Categorical	Cross–sectional	0.7273	0.5013	1.0551
Intercept	0.3817	0.0418	9.1333	<0.0001	0.2872	0.4763	Exposure Family	Categorical	Chewing dysfunction	1.4648	1.3327	1.6100
OHRQoL/ oral impacts	0.1390	0.1811	0.7674	0.4625	–0.2707	0.5488	Exposure Family	Categorical	Chewing dysfunction	1.1491	0.7628	1.7311
Oral frailty/ functional decline	0.7172	0.1426	5.0295	0.0007	0.3946	1.0397	Exposure Family	Categorical	Chewing dysfunction	2.0486	1.4838	2.8284
Other oral impairment	–0.2662	0.0577	–4.6094	0.0013	–0.3968	–0.1356	Exposure Family	Categorical	Chewing dysfunction	0.7663	0.6724	0.8732
Tooth loss/ dentition	–0.1006	0.0733	–1.3733	0.2029	–0.2664	0.0651	Exposure Family	Categorical	Chewing dysfunction	0.9043	0.7661	1.0673
Intercept	0.4869	0.1123	4.3348	0.0008	0.2442	0.7296	Region	Categorical	Asia	1.6273	1.2767	2.0742
Non–Asia	–0.0304	0.2631	–0.1157	0.9097	–0.5989	0.5380	Region	Categorical	Asia	0.9700	0.5494	1.7126
Intercept	0.3098	0.1653	1.8740	0.0855	–0.0504	0.6701	Ascertainment Type	Categorical	Likely clinical/ objective or structured	1.3632	0.9508	1.9544
Likely self–report or mixed	0.5299	0.2218	2.3897	0.0342	0.0468	1.0131	Ascertainment Type	Categorical	Likely clinical/ objective or structured	1.6988	1.0479	2.7541

Unclear	0.0177	0.1952	0.0907	0.9292	–0.4076	0.4430	Ascertainment Type	Categorical	Likely clinical/ objective or structured	1.0179	0.6652	1.5574
Intercept	0.4747	0.1066	4.4549	0.0006	0.2445	0.7049	NOS Methodological Quality Category	Categorical	High	1.6075	1.2770	2.0236
Moderate	0.0474	0.3369	0.1406	0.8904	–0.6805	0.7752	NOS Methodological Quality Category	Categorical	High	1.0485	0.5064	2.1710
Intercept	1.2183	0.7982	1.5263	0.1509	–0.5061	2.9426	NOS Total Score	Continuous	Not applicable	3.3813	0.6028	18.9659
NOS Total Score	–0.0998	0.1061	–0.9399	0.3644	–0.3291	0.1295	NOS Total Score	Continuous	Not applicable	0.9050	0.7196	1.1383

Notes: This table reports coefficient–level results from the exploratory univariate mixed–effects meta–regression models fitted to the primary Odds ratio (OR)–only backbone. Each model included a single moderator at a time. For categorical moderators, the intercept corresponds to the stated reference category, and the additional rows represent contrasts relative to that reference category. For the continuous moderator (NOS Total Score), the intercept is mathematically defined as the model–predicted value when the moderator equals zero; this quantity is included for model completeness but is not substantively interpretable for the observed review corpus. Coefficients are presented on the logarithmic scale together with Hartung–Knapp–Sidik–Jonkman (HKSJ) standard errors, t statistics, p values, and 95% confidence intervals (CIs). Back–transformed estimates on the ratio scale are shown to facilitate interpretation. Because these meta–regression models were based on a modest number of studies and some moderator levels were sparsely populated, the results should be interpreted cautiously and should not be regarded as definitive evidence of effect modification. Values smaller than 0.0001 are reported as <0.0001.

Table S15. Study-level influence diagnostics for the primary Odds ratio (OR)-only random-effects model.

Study (ID, Authors, Year)	Study–Specific OR	Lower 95% CI	Upper 95% CI	Leverage (Hat Value)	Standardized Residual	Cook's Distance	Baujat Q Contribution	Baujat Absolute Shift in Pooled OR	Leave–One–Out I^2 (%)	Leave–One–Out τ^2 (REML)	Flag: ∣Standardized Residual∣ > 1.96	Flag: Cook's Distance > 4/k
IN–003/ Kawamura et al. (2026) [94]	2.123	1.270	3.550	0.047	0.709	0.029	0.568	0.023	88.600	0.083	No	No
IN–004/ Koh et al. (2026) [95]	1.497	1.110	2.020	0.067	–0.230	0.005	0.938	0.019	88.856	0.093	No	No
IN–006/ Kusama et al. (2021) [97]	1.300	1.120	1.510	0.080	–0.728	0.057	8.102	0.042	88.923	0.090	No	No
IN–007/ Lin et al. (2022) [98]	4.882	2.020	11.800	0.025	2.076	0.125	8.928	0.059	88.062	0.068	Yes	No
IN–011/ Miyano et al. (2025) [102]	1.257	1.000	1.580	0.073	–0.808	0.064	4.926	0.043	88.910	0.089	No	No
IN–012/ Muniz et al. (2021) [103]	1.296	0.725	2.318	0.041	–0.531	0.014	0.703	0.023	88.925	0.088	No	No
IN–014/ Ohi et al. (2022) [105]	6.000	1.380	26.090	0.011	1.637	0.033	3.553	0.029	88.525	0.077	No	No
IN–015/ Park et al. (2016) [106]	1.486	1.396	1.581	0.085	–0.286	0.009	48.654	0.026	86.327	0.095	No	No
IN–016/ Park et al. (2023) [107]	1.113	1.053	1.177	0.086	–1.292	0.193	29.126	0.058	76.783	0.075	No	No

IN–017/ Rouxel et al. (2018) [108]	1.606	1.200	2.150	0.068	–0.013	0.000	0.001	0.011	88.751	0.093	No	No
IN–020/ Sun et al. (2025) [111]	3.136	2.430	4.046	0.072	2.116	0.419	8.473	0.192	81.926	0.018	Yes	Yes
IN–022/ Takiguchi et al. (2016) [113]	2.163	1.200	3.900	0.041	0.707	0.025	0.999	0.024	88.660	0.083	No	No
IN–025/ Wang et al. (2026) [116]	1.337	1.106	1.617	0.077	–0.621	0.040	8.910	0.038	88.922	0.092	No	No
IN–026/ Wei et al. (2024) [117]	1.818	1.157	2.858	0.053	0.326	0.007	1.496	0.011	88.735	0.088	No	No
IN–028/ Yamamoto et al. (2017) [119]	1.284	1.030	1.600	0.074	–0.744	0.055	6.436	0.041	88.920	0.090	No	No

Notes: Influence diagnostics were calculated for the primary Odds ratio (OR)–only backbone using the original unrounded analytical files corresponding to the study–level data summarized in Table S8. The underlying model was an intercept–only random–effects meta–analysis with inverse–variance weighting and restricted maximum likelihood (REML) estimation of between–study variance. Leverage (hat value) reflects the relative influence of each study in the weighted intercept–only model. Standardized residual denotes the residual on the logarithmic effect scale standardized by the study–specific residual standard deviation under the random–effects model. Cook's distance summarizes the joint influence of each study on model fit and the pooled estimate. Baujat Q Contribution represents the study–specific contribution to the overall heterogeneity statistic (Q), and Baujat Absolute Shift in Pooled OR denotes the absolute change in the pooled odds ratio after exclusion of the indicated study. Leave–One–Out I^2 (%) and Leave–One–Out τ^2 (REML) report the heterogeneity estimates after refitting the model with the specified study omitted. The flag |Standardized Residual| > 1.96 identifies studies with comparatively large residuals, whereas Cook's Distance > 4/k identifies studies exceeding a conventional influence threshold, with k = 15. Values displayed in the table are rounded for presentation only.

Table S16. Study-level Baujat diagnostic data for the primary Odds ratio (OR)-only backbone.

Record ID	Study	Baujat Q Contribution	Baujat Absolute Shift in Pooled OR	Combined Influence Rank Score
IN–007	Lin et al. (2022) [98]	8.928	0.059	5.0
IN–016	Park et al. (2023) [107]	29.126	0.058	5.0
IN–020	Sun et al. (2025) [111]	8.473	0.192	6.0
IN–015	Park et al. (2016) [106]	48.654	0.026	10.0
IN–006	Kusama et al. (2021) [97]	8.102	0.042	11.0
IN–025	Wang et al. (2026) [116]	8.910	0.038	11.0
IN–011	Miyano et al. (2025) [102]	4.926	0.043	12.0
IN–028	Yamamoto et al. (2017) [119]	6.436	0.041	13.0
IN–014	Ohi et al. (2022) [105]	3.553	0.029	17.0
IN–022	Takiguchi et al. (2016) [113]	0.999	0.024	21.0
IN–012	Muniz et al. (2021) [103]	0.703	0.023	24.5
IN–026	Wei et al. (2024) [117]	1.496	0.011	24.5
IN–004	Koh et al. (2026) [95]	0.938	0.019	25.0
IN–003	Kawamura et al. (2026) [94]	0.568	0.023	25.5
IN–017	Rouxel et al. (2018) [108]	0.001	0.011	29.5

Notes: Baujat diagnostic quantities were calculated for the primary Odds ratio (OR)–only backbone using the original unrounded analytical files corresponding to the study–level data summarized in Table S8. Baujat Q Contribution denotes the study–specific contribution to the total heterogeneity statistic in the intercept–only random–effects model. Baujat Absolute Shift in Pooled OR represents the absolute change in the pooled odds ratio after omitting the indicated study and refitting the model. Combined Influence Rank Score was derived by summing the descending ranks of (i) the study–specific Baujat Q Contribution and (ii) the Baujat Absolute Shift in Pooled OR; tied values were assigned average ranks. Lower combined scores indicate greater overall influence in the joint Baujat framework. The table is intended to complement the influence diagnostics and leave–one–out analyses by identifying studies that contribute substantially to heterogeneity and/or materially affect the pooled estimate. Values displayed in the table are rounded for presentation only.
