# Supplementary material for: Oral Structural and Functional Impairment in Relation to Depressive Outcomes in Older Adults: A Systematic Review and Meta-Analysis of Observational Studies
Source: Diseases. 2026 Aug 26;14(9):309. doi: 10.3390/diseases14090309 (PMC13605695; doi:10.3390/diseases14090309)
Supplement: Supplementary file 1 [file diseases-14-00309-s001.zip › diseases-4470095_PRISMA_2020_checklist.pdf]

PRISMA 2020 Checklist

Section and Topic	Item #	Checklist item	Location where item is reported
**TITLE**			
Title	1	Identify the report as a systematic review.<br>Response: The title explicitly identifies the article as a systematic review and meta-analysis: “Oral Structural and Functional Impairment in Relation to Depressive Outcomes in Older Adults: A Systematic Review and Meta-analysis of Observational Studies”.	Manuscript: Title (front page).
**ABSTRACT**			
Abstract	2	See the PRISMA 2020 for Abstracts checklist.<br>Response: The structured abstract reports the review objective, six databases and search dates, eligibility, Newcastle–Ottawa Scale risk-of-bias assessment, GRADE certainty assessment, the primary random-effects OR meta-analysis, substantial heterogeneity, the trim-and-fill sensitivity result, and the very-low-certainty interpretation.	Manuscript: Abstract.
**INTRODUCTION**			
Rationale	3	Describe the rationale for the review in the context of existing knowledge.<br>Response: The rationale for the review is described in the context of existing knowledge, including the clinical/public-health relevance of late-life depression and oral impairment, and the biological, functional, and psychosocial pathways linking oral impairment to depressive outcomes.	Manuscript: Introduction, Sections 1.1–1.3.
Objectives	4	Provide an explicit statement of the objective(s) or question(s) the review addresses.<br>Response: The review objective and focused review question are explicitly stated, together with the primary and secondary objectives for the synthesis	Manuscript: Introduction, Section 1.4.
**METHODS**			
Eligibility criteria	5	Specify the inclusion and exclusion criteria for the review and how studies were grouped for the syntheses.<br>Response: Inclusion and exclusion criteria were prespecified for observational studies in older adults or separately extractable older-adult subgroups. Oral structural/functional constructs, broader proxy indicators, depressive-outcome classes, directionality, and analytic-stream assignment are explicitly defined, with construct-specific groupings retained for synthesis.	Manuscript: Methods 2.2 and 2.6–2.8; Supplementary Materials: Tables S2–S10 and S12.
Information sources	6	Specify all databases, registers, websites, organisations, reference lists and other sources searched or consulted to identify studies. Specify the date when each source was last searched or consulted.<br>Response: All information sources are listed: MEDLINE/PubMed, Scopus, Web of Science Core Collection, Embase, APA PsycInfo, and CINAHL. Original searches were executed on 5 January 2026 and an updated search was rerun on 19 April 2026.	Manuscript: Abstract; Methods 2.3; Results 3.1 and Figure 1 caption; Supplementary Materials: Table S1 and Table S1 Notes.
Search strategy	7	Present the full search strategies for all databases, registers and websites, including any filters and limits used.<br>Response: Full database-specific strategies are provided for all six databases, including controlled-vocabulary and free-text terms, Boolean structure, field tags, search dates, date limits, language restrictions, and platform-specific syntax. No restrictive observational study-design filters were applied at the database-search stage.	Supplementary Materials: Table S1 and Table S1 Notes.
Selection process	8	Specify the methods used to decide whether a study met the inclusion criteria of the review, including how many reviewers screened each record and each report retrieved, whether they worked independently, and if applicable, details of automation tools used in the process.<br>Response: Title/abstract screening and full-text assessment were performed independently by two reviewers. Any record considered potentially eligible by either reviewer advanced to full-text review. Disagreements were resolved by discussion and, when needed, by adjudication from a third reviewer. No automation tools were reported.	Manuscript: Methods 2.4.

Section and Topic	Item #	Checklist item	Location where item is reported
Data collection process	9	Specify the methods used to collect data from reports, including how many reviewers collected data from each report, whether they worked independently, any processes for obtaining or confirming data from study investigators, and if applicable, details of automation tools used in the process.<br>Response: Data were extracted using a structured and piloted form. Primary extraction was conducted by one reviewer and all extracted fields were independently verified by a second reviewer against the full text; discrepancies were resolved by direct source re-checking and, if necessary, third-reviewer adjudication. No automation tools or study-investigator contact procedures were reported.	Manuscript: Methods 2.5.
Data items	10a	List and define all outcomes for which data were sought. Specify whether all results that were compatible with each outcome domain in each study were sought (e.g. for all measures, time points, analyses), and if not, the methods used to decide which results to collect.<br>Response: Outcomes sought were depression-related endpoints, including diagnosis or newly diagnosed depression, binary depressive symptoms, incident depressive outcomes, and continuous depressive symptom burden/severity. Original study instruments and cutoffs were retained, continuous scores were not dichotomized post hoc, and one primary estimate per study per analytic stream was retained when multiple eligible results were available.	Manuscript: Methods 2.2 and 2.6; Supplementary Materials: Tables S4 and S6–S10.
	10b	List and define all other variables for which data were sought (e.g. participant and intervention characteristics, funding sources). Describe any assumptions made about any missing or unclear information.<br>Response: Other variables sought included bibliographic information, country/region, study design, age framing, exposure definition and family, outcome definition, adjustment status, effect metric, effect estimate, confidence interval, and planned analytic-stream assignment. Assumptions and prioritization rules for multiple eligible estimates and older-adult subgroup extractability are described.	Manuscript: Methods 2.5–2.6; Supplementary Materials: Tables S4 and S6.
Study risk of bias assessment	11	Specify the methods used to assess risk of bias in the included studies, including details of the tool(s) used, how many reviewers assessed each study and whether they worked independently, and if applicable, details of automation tools used in the process.<br>Response: Risk of bias was assessed independently by two reviewers, with disagreements resolved through discussion and, when necessary, third-reviewer adjudication. Cohort studies used the standard Newcastle–Ottawa Scale (NOS); analytical cross-sectional studies used a prespecified adapted 0–9-star NOS framework with explicit operational rules for Selection (0–4), Comparability (0–2), and Outcome/Exposure (0–3).	Manuscript: Methods 2.7; Supplementary Materials: Table S5, Panel A and “Analytical cross-sectional adaptation: explicit scoring rules.”
Effect measures	12	Specify for each outcome the effect measure(s) (e.g. risk ratio, mean difference) used in the synthesis or presentation of results.<br>Response: Effect measures were specified by analytic stream: odds ratios for the primary pooled binary-outcome synthesis; risk ratios and prevalence ratios for the secondary ratio-based narrative stream; and adjusted beta coefficients, standardized beta coefficients, total effect coefficients, and two-stage least squares coefficients for the secondary continuous-effect narrative stream. Odds ratios were harmonized on the log scale with corresponding standard errors and variances for synthesis.	Manuscript: Methods 2.8–2.9; Supplementary Materials: Tables S6 and S8–S10.

Section and Topic	Item #	Checklist item	Location where item is reported
Synthesis methods	13a	Describe the processes used to decide which studies were eligible for each synthesis (e.g. tabulating the study intervention characteristics and comparing against the planned groups for each synthesis (item #5)).<br>Response: Studies were assigned to prespecified analytic streams after full extraction, exposure/outcome classification, and metric comparability assessment. Eligibility for each synthesis was determined using the frozen master effect-selection dataset and the stream-assignment/reconciliation framework.	Manuscript: Methods 2.5 and 2.8; Results 3.1–3.2; Supplementary Materials: Tables S6–S10.
	13b	Describe any methods required to prepare the data for presentation or synthesis, such as handling of missing summary statistics, or data conversions.<br>Response: Data preparation methods included effect-selection prioritization, directionality harmonization, conversion of retained odds ratios to the log scale, derivation of standard errors from 95% confidence intervals when needed, and variance calculation for inverse-variance synthesis.	Manuscript: Methods 2.8; Supplementary Materials: Tables S6 and S8–S10.
	13c	Describe any methods used to tabulate or visually display results of individual studies and syntheses.<br>Response: Results are displayed using a PRISMA flow diagram, forest plot, Baujat plot, funnel plot, trim-and-fill plot, structured narrative results, main-text summary tables, and detailed supplementary tables. Table 1 presents both the overall cross-domain OR synthesis (Panel A) and exposure-family-stratified estimates (Panel B).	Manuscript: Figure 1; Figures 2–5; Tables 1–2; Results 3.1–3.11; Supplementary Materials: Tables S2–S16.
	13d	Describe any methods used to synthesize results and provide a rationale for the choice(s). If meta-analysis was performed, describe the model(s), method(s) to identify the presence and extent of statistical heterogeneity, and software package(s) used.<br>Response: The primary quantitative synthesis used inverse-variance random-effects meta-analysis of log odds ratios with restricted maximum likelihood estimation and Hartung–Knapp–Sidik–Jonkman inference. Heterogeneity was quantified with Cochran's Q, I^2, τ^2, τ, Higgins' H, and 95% prediction intervals. Secondary ratio-based and coefficient-based streams were synthesized narratively because of metric and interpretive non-equivalence. Analyses were conducted in Python 3.12.13 using reproducible scripted workflows.	Manuscript: Methods 2.5 and 2.9.1–2.9.7.
	13e	Describe any methods used to explore possible causes of heterogeneity among study results (e.g. subgroup analysis, meta-regression).<br>Response: Possible causes of heterogeneity were explored through prespecified subgroup analyses by design class, oral-exposure family, geographic region, ascertainment type, methodological-quality category, and older-adult population framing, together with exploratory univariate mixed-effects meta-regression within the primary OR-only backbone.	Manuscript: Methods 2.9.2–2.9.3; Results 3.6–3.7; Supplementary Materials: Tables S12–S14.

PRISMA 2020 Checklist

Section and Topic	Item #	Checklist item	Location where item is reported
	13f	Describe any sensitivity analyses conducted to assess robustness of the synthesized results.<br>Response: Sensitivity analyses included leave-one-out refitting, study-level influence diagnostics, Baujat assessment, and trim-and-fill sensitivity analysis for possible small-study effects.	Manuscript: Methods 2.9.4–2.9.5; Results 3.8–3.9; Table 2; Figures 3–5; Supplementary Materials: Tables S11, S15, and S16.
Reporting bias assessment	14	Describe any methods used to assess risk of bias due to missing results in a synthesis (arising from reporting biases).<br>Response: Possible small-study effects were assessed by visual inspection of the funnel plot and by a regression-based asymmetry test when the number of pooled studies was sufficient. Trim-and-fill was used as a sensitivity analysis rather than as a corrected causal estimate.	Manuscript: Methods 2.9.5.
Certainty assessment	15	Describe any methods used to assess certainty (or confidence) in the body of evidence for an outcome.<br>Response: Certainty of evidence was assessed using GRADE at the level of each synthesized body of evidence. Observational evidence started at low certainty and was evaluated across risk of bias, inconsistency, indirectness, imprecision, and publication bias, with upgrading factors considered when appropriate.	Manuscript: Methods 2.7; Supplementary Materials: Table S5, Panel B.
**RESULTS**			
Study selection	16a	Describe the results of the search and selection process, from the number of records identified in the search to the number of studies included in the review, ideally using a flow diagram.<br>Response: The search and selection process is reported numerically from records identified through screening and full-text assessment to the final included review corpus, and the process is displayed in the PRISMA 2020 flow diagram.	Manuscript: Results 3.1; Figure 1.
	16b	Cite studies that might appear to meet the inclusion criteria, but which were excluded, and explain why they were excluded.<br>Response: Excluded full-text reports are cited in the exclusion log, and each exclusion is accompanied by a report-level reason for non-inclusion.	Manuscript: Results 3.1; Supplementary Materials: Table S3.
Study characteristics	17	Cite each included study and present its characteristics.<br>Response: Characteristics of the included studies are summarized in the manuscript and presented in detail in the supplementary study-level synopsis and consolidated characteristics table.	Manuscript: Results 3.2 and 3.4; Supplementary Materials: Tables S2 and S4.
Risk of bias in studies	18	Present assessments of risk of bias for each included study.<br>Response: Study-level methodological quality assessments are presented for all included studies using the Newcastle–Ottawa Scale, and body-level certainty judgments are reported separately using GRADE.	Manuscript: Results 3.3; Supplementary Materials: Table S5, Panels A and B.

Section and Topic	Item #	Checklist item	Location where item is reported
Results of individual studies	19	For all outcomes, present, for each study: (a) summary statistics for each group (where appropriate) and (b) an effect estimate and its precision (e.g. confidence/credible interval), ideally using structured tables or plots.<br>Response: For individual studies, effect estimates and their precision are presented in structured study-level tables for the primary OR-only backbone and the secondary RR/PR and coefficient streams; individual-study patterns are also described narratively by exposure family in the Results.	Manuscript: Results 3.4, 3.10, and 3.11; Supplementary Materials: Tables S4 and S8–S10.
Results of syntheses	20a	For each synthesis, briefly summarise the characteristics and risk of bias among contributing studies.<br>Response: For each synthesis, the contributing study designs, exposure domains, and methodological-quality context are summarized before or alongside the synthesis-specific results.	Manuscript: Results 3.2–3.5, 3.10, and 3.11; Supplementary Materials: Tables S5 and S8–S10.
	20b	Present results of all statistical syntheses conducted. If meta-analysis was done, present for each the summary estimate and its precision (e.g. confidence/credible interval) and measures of statistical heterogeneity. If comparing groups, describe the direction of the effect.<br>Response: All statistical syntheses are reported. Table 1 presents the overall primary OR-only estimate and heterogeneity statistics in Panel A and exposure-family-stratified pooled estimates in Panel B; Table 2 presents the trim-and-fill sensitivity model. Secondary RR/PR and coefficient-based streams are reported in structured quantitative narrative form because their metrics are not exchangeable.	Manuscript: Results 3.5 and 3.9–3.11; Tables 1–2; Figures 2, 4, and 5; Supplementary Materials: Tables S8–S10 and S12.
	20c	Present results of all investigations of possible causes of heterogeneity among study results.<br>Response: Results of subgroup analyses and exploratory meta-regression are reported for study design, oral-exposure family, ascertainment type, region, methodological quality, and other prespecified study-level characteristics, with explicit caution for sparse strata and limited moderator power.	Manuscript: Results 3.6–3.7; Supplementary Materials: Tables S12–S14.
	20d	Present results of all sensitivity analyses conducted to assess the robustness of the synthesized results.<br>Response: Results of leave-one-out analyses, influence diagnostics, Baujat assessment, and trim-and-fill sensitivity analysis are presented to assess the robustness of the primary synthesis.	Manuscript: Results 3.8–3.9; Table 2; Figures 3–5; Supplementary Materials: Tables S11, S15, and S16.
Reporting biases	21	Present assessments of risk of bias due to missing results (arising from reporting biases) for each synthesis assessed.<br>Response: Assessments of possible small-study effects and trim-and-fill sensitivity analysis are presented for the primary pooled synthesis.	Manuscript: Results 3.9; Table 2; Figures 4–5.

PRISMA 2020 Checklist

Section and Topic	Item #	Checklist item	Location where item is reported
Certainty of evidence	22	Present assessments of certainty (or confidence) in the body of evidence for each outcome assessed.<br>Response: Body-level GRADE assessments are reported for the primary OR-only backbone, the secondary RR/PR stream, the secondary coefficient-based stream, and the overall qualitative evidence base. All four evidence bodies are rated very low certainty, with the reasons for downgrading stated explicitly.	Manuscript: Results 3.3; Supplementary Materials: Table S5, Panel B.
**DISCUSSION**			
Discussion	23a	Provide a general interpretation of the results in the context of other evidence.<br>Response: The discussion provides an integrated interpretation of the findings in relation to the prior literature and the proposed biological, functional, and psychosocial pathways linking oral impairment with depressive outcomes.	Manuscript: Discussion 4.1–4.4; Conclusions 5.
	23b	Discuss any limitations of the evidence included in the review.<br>Response: Limitations of the included evidence are discussed, including observational design, reverse causation and residual confounding, substantial heterogeneity, sparse subgroup strata, possible small-study effects, heterogeneous oral/depression definitions, geographic concentration and limited transportability, limitations of the NOS, sparse non-OR streams, and the 2016 publication-date restriction.	Manuscript: Discussion 4.5.
	23c	Discuss any limitations of the review processes used.<br>Response: Limitations of the review process are discussed, including the absence of prospective public registration and independent time-stamping, the 2016 date-window restriction applied at database retrieval, the resulting inability to quantify unscreened pre-2016 records without a de novo search, and interpretive constraints arising from metric non-equivalence and sparse exploratory moderator strata.	Manuscript: Methods 2.1 and 2.3; Discussion 4.5; Supplementary Materials: Tables S6–S7.
	23d	Discuss implications of the results for practice, policy, and future research.<br>Response: Implications for future research, multidisciplinary geriatric/gerontopsychiatric assessment, clinical practice, and policy are discussed, with explicit caution against causal, prognostic, or treatment interpretation beyond the observational evidence.	Manuscript: Discussion 4.4 and 4.6; Conclusions 5.
**OTHER INFORMATION**			
Registration and protocol	24a	Provide registration information for the review, including register name and registration number, or state that the review was not registered.<br>Response: The review was not prospectively registered. Specifically, no prospective registration was obtained in PROSPERO, the Open Science Framework (OSF) Registries, or another public registry.	Manuscript: Methods 2.1.
	24b	Indicate where the review protocol can be accessed, or state that a protocol was not prepared.<br>Response: A separate prospectively registered protocol was not prepared for public access. No retrospective registration was undertaken because the review and quantitative synthesis had already progressed beyond the stage at which registration could serve its intended prospective function.	Manuscript: Methods 2.1.
	24c	Describe and explain any amendments to information provided at registration or in the protocol.<br>Response: Not applicable because no registered or separately accessible prospective protocol was available. The frozen effect-selection dataset and analytic-stream reconciliation are reported as auditability safeguards, but the manuscript explicitly states that they cannot provide independent prospective verification of when eligibility, exposure, effect-selection, or analytic decisions were fixed.	Manuscript: Methods 2.1; Supplementary Materials: Tables S6–S7.
Support	25	Describe sources of financial or non-financial support for the review, and the role of the funders or sponsors in the review.<br>Response: Sources of support are reported. The review received no external funding, and article processing charges were funded by the University “Dunărea de Jos” of Galați, Romania.	Manuscript: Funding.
Competing interests	26	Declare any competing interests of review authors.<br>Response: Competing interests are declared.	Manuscript: Conflicts of Interest.

PRISMA 2020 Checklist

Section and Topic	Item #	Checklist item	Location where item is reported
Availability of data, code and other materials	27	Report which of the following are publicly available and where they can be found: template data collection forms; data extracted from included studies; data used for all analyses; analytic code; any other materials used in the review.<br>Response: No individual participant data were generated. Data were extracted from published articles, and the derived study-level extraction/effect-selection datasets and analytic tables are provided in the Supplementary Materials, including search strategies, the exclusion log, study characteristics, NOS/GRADE assessments, frozen analytic-stream datasets, subgroup/meta-regression outputs, and sensitivity/influence diagnostics. Analytic code is not reported as publicly available.	Manuscript: Data Availability Statement; Methods 2.5; Supplementary Materials: Tables S1–S16.

From: Page MJ, McKenzie JE, Bossuyt PM, Boutron I, Hoffmann TC, Mulrow CD, et al. The PRISMA 2020 statement: an updated guideline for reporting systematic reviews. BMJ 2021;372:n71. doi: 10.1136/bmj.n71. This work is licensed under CC BY 4.0. To view a copy of this license, visit https://creativecommons.org/licenses/by/4.0/
